# Life history traits and cancer prevalence in birds

**DOI:** 10.1093/emph/eoae011

**Published:** 2024-06-27

**Authors:** Stefania E Kapsetaki, Zachary T Compton, Jordyn Dolan, Valerie Κ Harris, Walker Mellon, Shawn M Rupp, Elizabeth G Duke, Tara M Harrison, Selin Aksoy, Mathieu Giraudeau, Orsolya Vincze, Kevin J McGraw, Athena Aktipis, Marc Tollis, Amy Μ Boddy, Carlo C Maley

**Affiliations:** Arizona Cancer Evolution Center, Arizona State University, Tempe, AZ, USA; Tufts University, School of Arts and Sciences, Department of Biology, 200 Boston Avenue, Suite 4600, Medford, MA, USA; Center for Biocomputing, Security and Society, Biodesign Institute, Arizona State University, Tempe, AZ, USA; Arizona Cancer Evolution Center, Arizona State University, Tempe, AZ, USA; University of Arizona Cancer Center, Tucson, AZ, USA; University of Arizona College of Medicine, Tucson, AZ, USA; Arizona Cancer Evolution Center, Arizona State University, Tempe, AZ, USA; Center for Biocomputing, Security and Society, Biodesign Institute, Arizona State University, Tempe, AZ, USA; Arizona Cancer Evolution Center, Arizona State University, Tempe, AZ, USA; Center for Biocomputing, Security and Society, Biodesign Institute, Arizona State University, Tempe, AZ, USA; Arizona Cancer Evolution Center, Arizona State University, Tempe, AZ, USA; Arizona Cancer Evolution Center, Arizona State University, Tempe, AZ, USA; Center for Biocomputing, Security and Society, Biodesign Institute, Arizona State University, Tempe, AZ, USA; Arizona Cancer Evolution Center, Arizona State University, Tempe, AZ, USA; Department of Clinical Sciences, North Carolina State University, Raleigh, NC, 27607, USA; Exotic Species Cancer Research Alliance, North Carolina State University, Raleigh, NC, 27607, USA; Arizona Cancer Evolution Center, Arizona State University, Tempe, AZ, USA; Department of Clinical Sciences, North Carolina State University, Raleigh, NC, 27607, USA; Exotic Species Cancer Research Alliance, North Carolina State University, Raleigh, NC, 27607, USA; Arizona Cancer Evolution Center, Arizona State University, Tempe, AZ, USA; School of Life Sciences, Arizona State University, Tempe, AZ, USA; Littoral Environnement Et Sociétés (LIENSs), UMR7266, CNRS Université de La Rochelle, 2 rue Olympe de Gouges, 17042, La Rochelle Cedex, France; Evolutionary Ecology Group, Hungarian Department of Biology and Ecology, Babeș-Bolyai University, Cluj-Napoca, Romania; Institute of Aquatic Ecology, Centre for Ecological Research, Debrecen, Hungary; School of Life Sciences, Arizona State University, Tempe, AZ, USA; Arizona Cancer Evolution Center, Arizona State University, Tempe, AZ, USA; Department of Psychology, Arizona State University, Tempe, AZ, USA; Arizona Cancer Evolution Center, Arizona State University, Tempe, AZ, USA; School of Informatics, Computing, and Cyber Systems, Northern Arizona University, PO Box 5693, Flagstaff, AZ 8601, USA; Arizona Cancer Evolution Center, Arizona State University, Tempe, AZ, USA; Exotic Species Cancer Research Alliance, North Carolina State University, Raleigh, NC, 27607, USA; Department of Anthropology, University of California Santa Barbara, CA, USA; Arizona Cancer Evolution Center, Arizona State University, Tempe, AZ, USA; Center for Biocomputing, Security and Society, Biodesign Institute, Arizona State University, Tempe, AZ, USA; School of Life Sciences, Arizona State University, Tempe, AZ, USA

**Keywords:** aves, cancer, neoplasia, life history evolution, malignancy, tumors

## Abstract

**Background and objectives:**

Cancer is a disease that affects nearly all multicellular life, including the broad and diverse taxa of Aves. While little is known about the factors that contribute to cancer risk across Aves, life history trade-offs may explain some of this variability in cancer prevalence. We predict birds with high investment in reproduction may have a higher likelihood of developing cancer. In this study, we tested whether life history traits are associated with cancer prevalence in 108 species of birds.

**Methodology:**

We obtained life history data from published databases and cancer data from 5,729 necropsies from 108 species of birds across 24 taxonomic orders from 25 different zoological facilities. We performed phylogenetically controlled regression analyses between adult body mass, lifespan, incubation length, clutch size, sexually dimorphic traits, and both neoplasia and malignancy prevalence. We also compared the neoplasia and malignancy prevalence of female and male birds.

**Results:**

Providing support for a life history trade-off between somatic maintenance and reproduction, we found a positive relationship between clutch size and cancer prevalence across Aves. There was no significant association with body mass, lifespan, incubation length, sexual dimorphism, and cancer.

**Conclusions and implications:**

Life history theory presents an important framework for understanding differences in cancer defenses across various species. These results suggest a trade-off between reproduction and somatic maintenance, where Aves with small clutch sizes get less cancer.

## INTRODUCTION

Nearly all multicellular organisms are susceptible to neoplastic disease [1, 2]. Neoplasia is a disease of uncontrolled cell division and growth, resulting ultimately in the formation of a tumor, as well as invasion or metastasis in the case of malignant neoplasia (a.k.a. cancer) [3]. Over the past few decades, cancer research has focused on identifying different molecular pathways, hallmarks, and control mechanisms of cancer—all with the ultimate aim of improving cancer treatment [4, 5]. Understanding why organisms differ in their ability to suppress cancer, as well as how they respond to neoplastic expansion, is a central question in comparative cancer research.

In general, life history trade-offs govern how organisms allocate time and resources to fitness components such as growth, self (or somatic)-maintenance, and reproduction [6, 7]. Somatic maintenance can include tumor suppression mechanisms such as cell cycle control and DNA damage repair. These trade-offs may help explain the variation in cancer prevalence across species. Larger and longer-lived species, due to their higher number of cells and longer time over which they may acquire mutations, are predicted to have much higher chances of developing cancer. However, paradoxically, this prediction is not supported by observations. Larger and longer-lived mammalian species do not have dramatically higher cancer prevalence or cancer risk than smaller and shorter-lived mammalian species, an observation that has come to be known as Peto’s Paradox [8–12]. This may be because large and long-lived species that invest in somatic maintenance over reproduction likely evolved enhanced mechanisms to suppress or evade cancer [13]. Utilizing this life history tradeoff approach can both give us insight into the basic biology and origins of cancer and also provide opportunities to discover either universal or novel mechanisms of cancer suppression that could have clinical applications to humans. For example, previous results across vertebrates have shown that life history traits, such as gestation length [14] and trophic levels [15, 16], but not longevity, litter size, or body mass [14], are significantly correlated with cancer prevalence across vertebrates. Within mammals, litter size [12, 17] and diet [11, 16], are significantly correlated with cancer prevalence or cancer risk, but litter size, gestation length, body mass, life expectancy, and lactation length are not significantly correlated with cancer risk in univariate analyses [18].

Birds (taxonomic class *Aves*) represent a diverse vertebrate taxon with considerable variation in life-history characteristics. This diversity in life history traits and the particular ecologies of birds suggests that they may differ from other vertebrates in the factors that explain why some birds are more or less susceptible to cancer.

Understanding cancer susceptibility among birds is currently an active area of research. For instance, Møller et al. surveyed free-living Eurasian birds post-mortem and found that, when analyzing at least 20 individuals per species, larger body size was correlated with tumor prevalence [19], while neither incubation nor nestling time was correlated with tumor prevalence [19]. These results suggest Peto’s observation that bigger species do not get more cancer, is not true in the Aves. Recently, Bulls et al. found that body mass and lifespan were not correlated with neoplasia prevalence in birds, with sample sizes ranging from 5 or 10 bird necropsies [20]. Separate studies have reported neoplasms (benign and malignant tumors combined) in bird species, either free-living or in human care [2, 21–26], but the prevalence of malignancy itself using larger sample sizes has not been measured before across bird species.

Beyond body mass and lifespan, there may also be a trade-off between reproductive investments and somatic maintenance [27], and therefore, cancer defenses. Birds can invest in reproduction in various formats, such as exaggerated morphological traits (e.g. sexually dimorphic or dichromatic color) [28–31], clutch size, and incubation length. Sexually dimorphic or dichromatic species with extreme phenotypes, such as large and colorful ornaments or weapons, may have an increased risk of cancer [27]. However, there has not been a study investigating the relationship between reproductive or sexually selected traits and cancer prevalence in birds.

To investigate the relationship between life history and cancer risk in birds, we expanded on previous life history studies in birds by including a wider range of life history traits from trait-rich life-history databases and compared these traits to cancer prevalence data from veterinary records of 108 bird species under managed care. This represents the second-largest study of cancer prevalence across bird species [19]. We hypothesized that the incredible diversity of life-history strategies observed across the class Aves can explain taxonomic differences in cancer risk in birds, due to the evolutionary trade-offs between growth, reproduction, and somatic maintenance. Specifically, we tested whether malignancy prevalence or neoplasia prevalence is correlated with other avian traits such as incubation length, clutch size, and degree of sexual dimorphism and dichromatism.

Lastly, species with the homogametic sex (e.g. XX females in mammals and ZZ males in birds) tend to live longer [32] and it has been proposed that the existence of two X chromosomes may offer some cancer protection to humans [33]. Therefore, we also tested whether male birds (ZZ sex chromosomes) have lower cancer prevalence than female birds (ZW sex chromosomes).

## METHODS

### Cancer and life-history data

We obtained 5729 individual adult necropsy records for 108 bird species (representing 24 orders) under human care from 25 different institutions over 25 years. Necropsies are typically performed by a veterinarian or veterinary pathologist who reviews each organ system. Through this process representative samples of each organ or representative samples of any abnormality found are submitted for histopathology. Through this process of necropsy and histopathology, the majority of neoplasms would be found. The necropsy records in our dataset were from Association of Zoos and Aquariums (AZA) institutions [34], where animals that die are required to be necropsied. We extracted the following data from these records: age at death (1287 individuals, 51 species, 17 orders), malignancies and neoplasias reported (5729 individuals, 108 species, 24 orders), and sex [34]. We measured malignancy (i.e. cancer) prevalence and neoplasia prevalence (benign and malignant tumor) for each species by dividing the total number of necropsies reporting malignancies (or neoplasms) by the total number of necropsies available for that species [35]; a measurement also used in previous studies [12, 16].

We assembled life-history variables from multiple published resources, including AnAge [36] and the Amniote Life History Database [37]. The collected life-history variables included species averages of adult body mass (g), lifespan (months), incubation length (months), clutch size (number of offspring per brood) [36, 37], presence and degree of sexual plumage dichromatism (plumage brightness and plumage hue) [38], and sexual size dimorphism (mass and tail size) [39].

### Data filtering

We only included bird species for which we had at least 20 necropsies in our analysis. For analyses comparing female and male malignancy prevalence or neoplasia prevalence, as well as sex bias regressions, we used species with at least 10 necropsy records per sex. We present the neoplasia and malignancy prevalence of 108 bird species [35]. We excluded chickens (*Gallus gallus*) from the analyses because as a largely domesticated agricultural species, they have been selected for egg-laying and frequently develop ovarian cancer [40]. We only included chickens (*Gallus gallus*) in Supplementary Table S1 and in the Supplementary Fig. 10 illustration of normalized frequency of the species’ age at death as a percentage of the species lifespan.

We excluded all infant data from our dataset because (i) the low prevalence of age-related diseases, such as cancer, in infants would likely bias the neoplasia prevalence data towards lower values and (ii) cancers in infants are medically different than adult cancers [41]. We defined infancy as a record’s age that is smaller or equal to that species’ age of infancy (or the average of male and female maturity). In cases of no records of infancy age, the record was considered an infant if it contained any of the following words: infant, juvenile, immature, adolescent, hatchling, subadult, neonate, newborn, offspring, and fledgling. We performed correlations between clutch size and neoplasia or cancer prevalence with and without removing domesticated and semi-domesticated species [42–51] (dataset [35]:). When comparing female and male malignancy prevalence and neoplasia prevalence, we removed all cases of reproductive cancer in order to minimize any effects of controlled reproduction in managed environments on our results.

### Ancestral state reconstruction phylogenies

Bird neoplasia and cancer prevalence may be driven by various different evolutionary forces. In order to find which evolutionary force is predominant in shaping these traits, we reconstructed ancestral state phylogenies. Utilizing TimeTree.org, and inputting the species of our dataset, we built a phylogeny that details the lineages of our bird species. With this, we used the Geiger and Phytools packages in R to create two ancestral state reconstruction phylogenies. These reconstructions provided estimates of neoplasia and malignancy prevalence across our bird species as well as their ancestors. In our reconstructions, we utilized a Markov Chain Monte Carlo (MCMC) to fit our data and estimate the best model of evolution. In order to determine the model of drift/selection that best fits our data, we compared Ornstein–Uhlenbeck (OU) to Brownian motion, to Early Burst models of evolution using Akaike information criterion comparisons to determine the best fit.

### Statistical analyses

We performed all statistical analyses in R version 4.0.5 [52]. We prepared figures using the data visualization software ggplot2 [53] and generated summary statistics in dplyr [54]. We tested whether the *P*-values passed the false discovery rate (FDR) correction in each of these 25 analyses (Supplementary Table S2A). To evaluate the robustness of our results, we carried out a subsampling analysis (Supplementary material: Supplementary subsampling results).

#### Life-history analyses

In order to test if a life history trait is statistically significantly associated with neoplasia or malignancy prevalence, we used phylogenetically controlled regressions. We performed all phylogenetic analyses using the R packages ape, Phytools, Geiger, Tidyverse, and Caper [55–59] using phylogenetic generalized least squares (PGLS) regressions to take into account the phylogenetic non-independence among species [60] and weighting analyses by 1/(square root of the number of necropsies per species) following Revell [57]. We uploaded our list of species to TimeTree (Timetree.org) and used the resulting phylogenetic tree for our phylogenetic regressions. Given that all life history trait data were not available for all species in the dataset, each phylogenetic regression had a different number of species. Thus, to perform each phylogenetic regression we pruned the above tree using the setdiff and keep.tip/drop.tip functions in R. We performed Grubbs’ and Rosner’s tests to identify and remove significant outliers in the PGLS analyses. We performed univariate and multivariate PGLS analyses excluding the significant outliers, and separate univariate PGLS analyses including the significant outliers. We ran the former to test if our results were mainly driven by species with extreme values. We also tested all analyses for the presence of any significant heteroscedasticity (Fligner–Killeen test), and if present, we mention this in our results. Because the malignancy and neoplasia prevalence data in our analysis is proportional, we transformed the neoplasia and malignancy prevalence by using an arcsine-square-root prior to running phylogenetic regressions and paired samples statistical tests. We used the ‘rr2’ package to obtain the *R*² values of the phylogenetic regressions. For visualization purposes, however, we display the data and regression lines in the figures using the non-arcsine-square-root-transformed data.

#### Sex difference analyses

There may be a trade-off between sexual selection and cancer defenses. To test for this, we quantified the degree of sexual dimorphism, as an indirect measure of sexual selection, in seven biometric variables [plumage brightness, plumage hue, mass (g), and tail size (g)] as the natural log of the male biometric variable divided by the natural log of the female biometric variable. We then used PGLS to test for associations of those sex differences with cancer prevalence. We also compared male malignancy prevalence or neoplasia prevalence versus female malignancy prevalence or neoplasia prevalence. The denominators in the case of the male malignancy prevalence or neoplasia prevalence are the total number of necropsied males, whereas the denominators in the case of the female malignancy prevalence or neoplasia prevalence are the total number of necropsied females. The distribution of the sex differences in cancer (i.e.‘female malignancy prevalence minus male malignancy prevalence’, ‘female neoplasia prevalence minus male neoplasia prevalence’) had significant outliers. Therefore, we compared malignancy prevalence and neoplasia prevalence between males and females using the non-parametric paired-samples sign test.

## RESULTS

**Figure 1.**
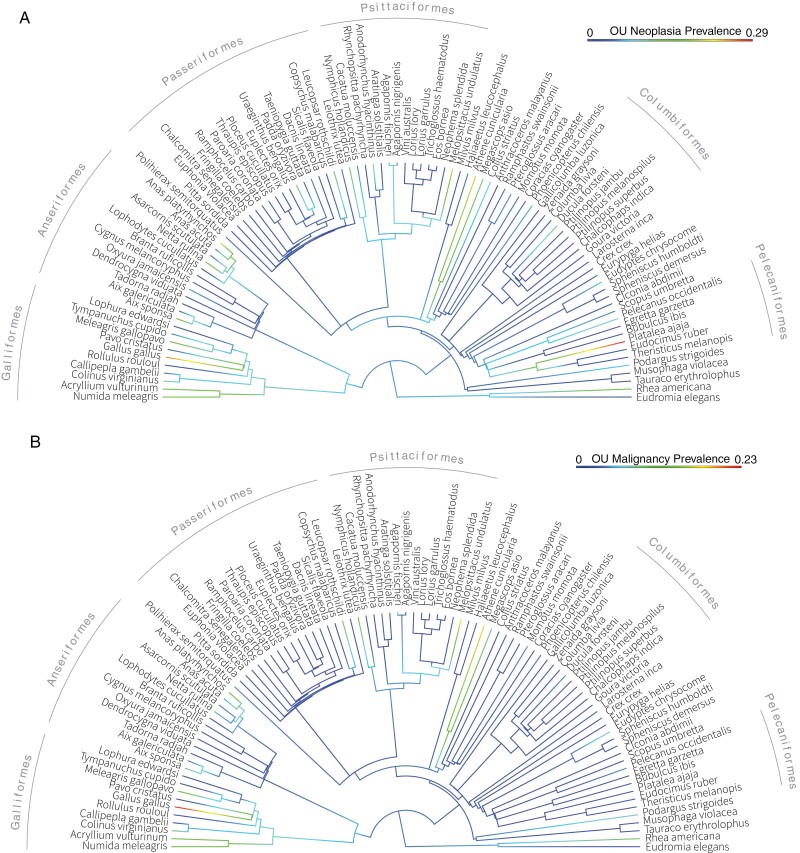
Neoplasia prevalence (**A**) and malignancy prevalence (**B**) across bird species in our dataset. These ancestral state reconstruction phylogenies are presented with the Ornstein-Uhlenbeck (OU) model; the model that best fits our data and phylogeny. The outer labels show the orders that the species belong to. We used a minimum of five species per order to avoid overlap in the labels. The color of the branches indicates the relative neoplasia prevalence (A) and malignancy prevalence (B) in each branch

To assess whether selection, rather than other evolutionary forces such as random genetic drift, can explain neoplasia and cancer prevalence across bird species, we reconstructed ancestral state phylogenies using the OU, Brownian, and Early Burst models of phenotype evolution. We found that the OU model best fit our cancer and neoplasia prevalence data and phylogeny (Fig. 1). These results suggest that stabilizing selection has played an important role in the evolution of neoplasia and cancer prevalence in birds (Fig. 1).

**Figure 2.**
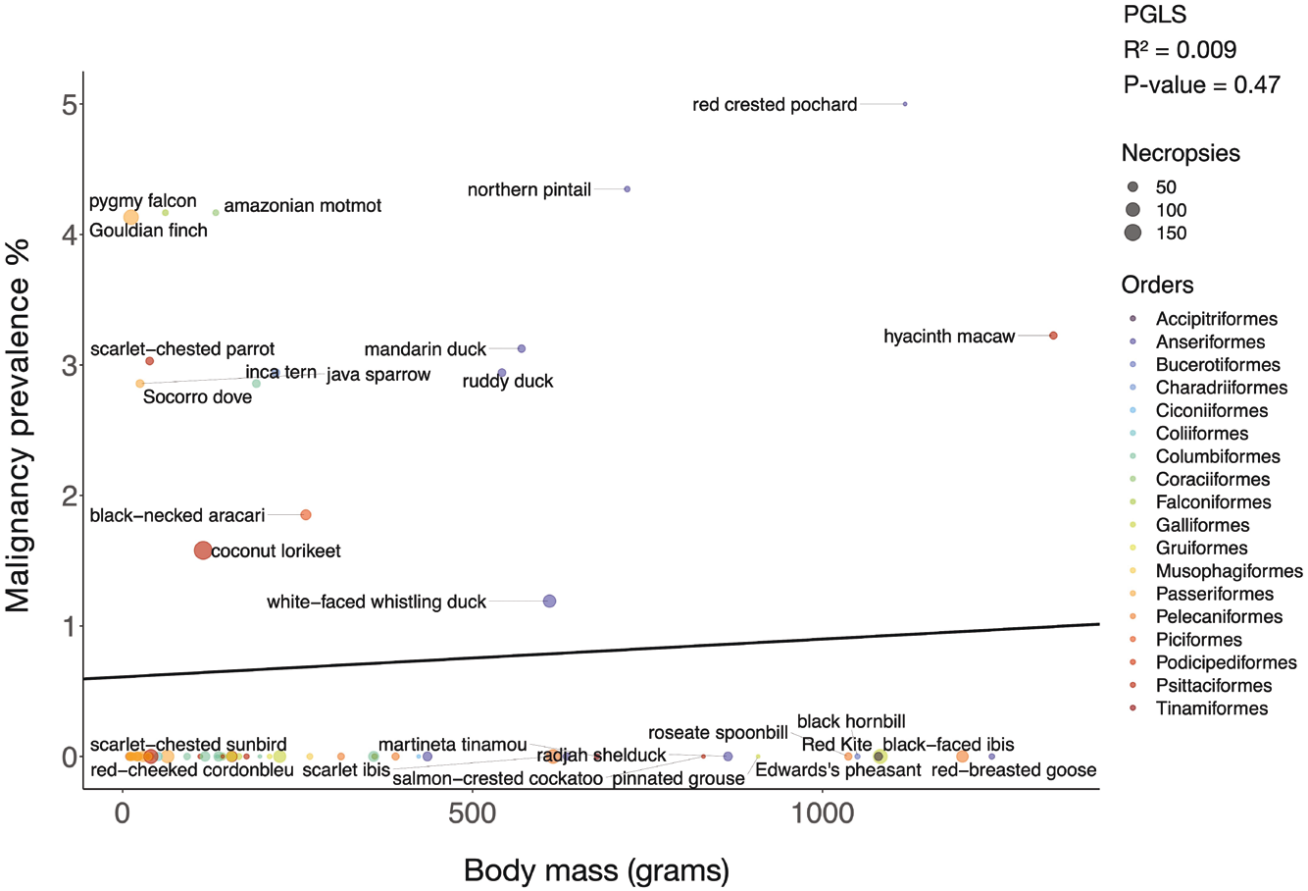
Larger body mass is not correlated with malignancy prevalence across 67 bird species. Dot size indicates the number of necropsies per species. Colors show the taxonomic order of each species, and the black line shows the phylogenetically controlled linear regression of body mass versus malignancy prevalence.

**Figure 3.**
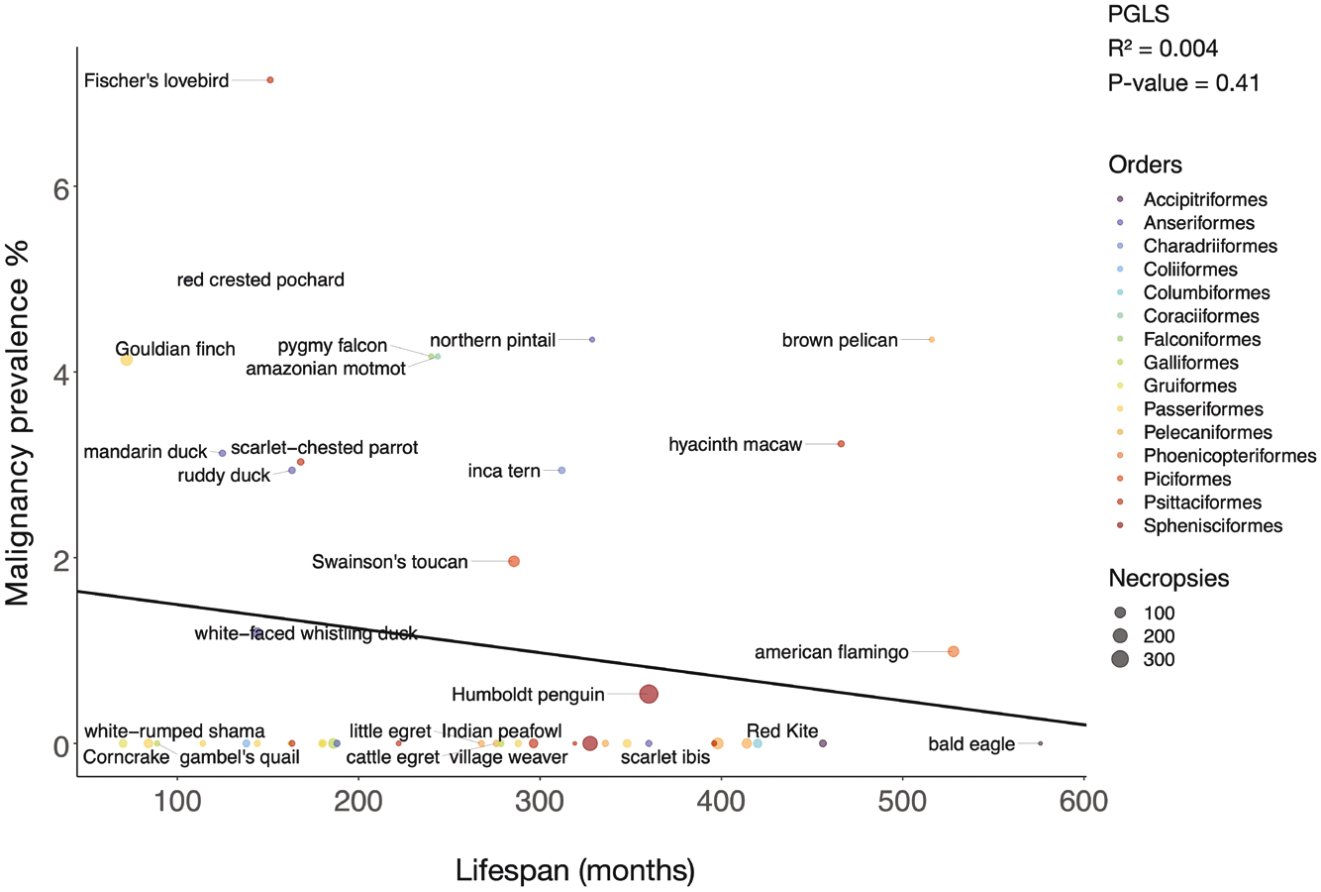
Longer lifespan is not correlated with malignancy prevalence across 45 bird species. Dot size indicates the number of necropsies per species. Colors show the taxonomic order of each species. The black line shows the phylogenetically controlled linear regression of lifespan versus malignancy prevalence

**Figure 4.**
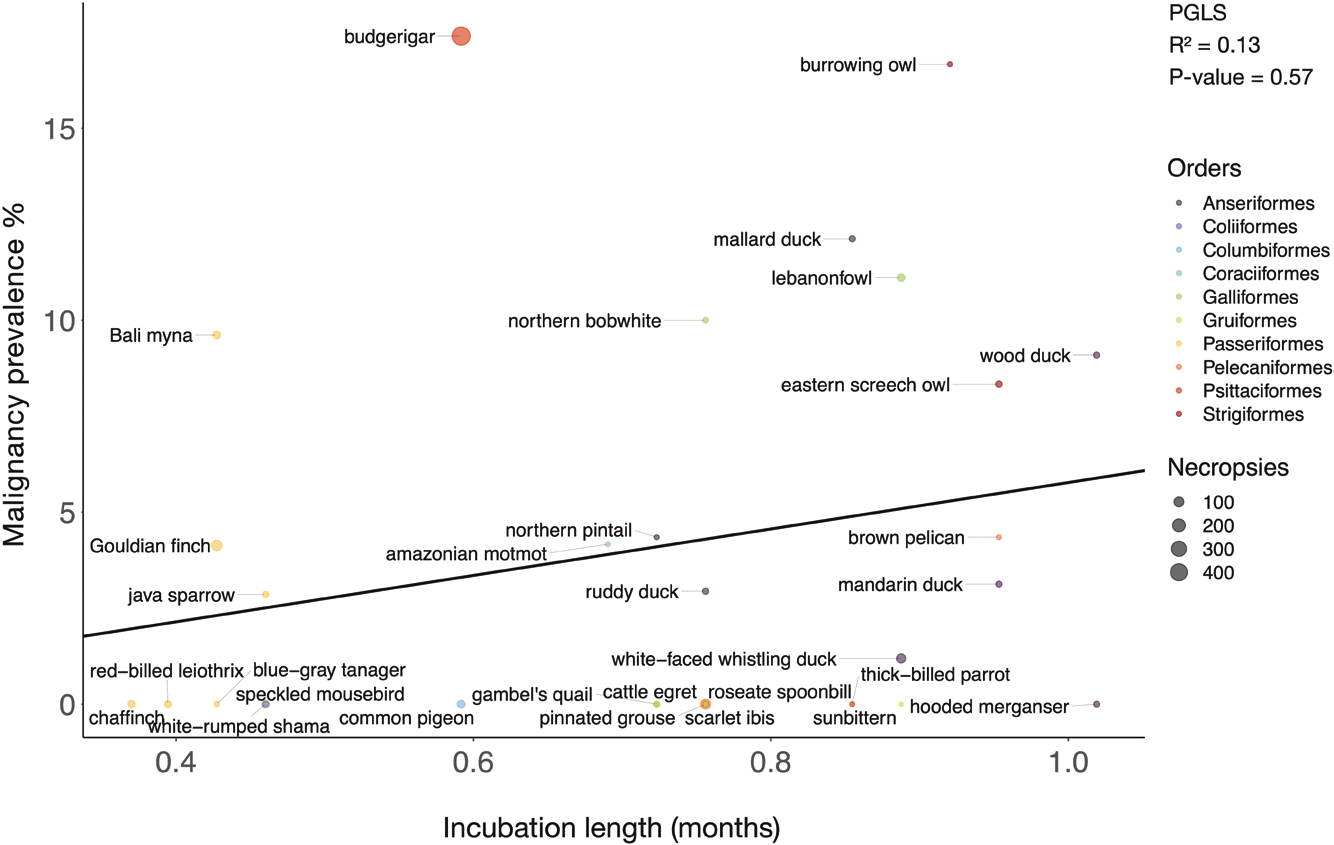
Incubation length is not correlated with malignancy prevalence when controlling for body mass and litter size across 30 species. Different colors indicate the order in which each species belongs and the size of the dot indicates the number of necropsies per species. The black line shows the phylogenetically controlled linear regression of incubation length versus malignancy prevalence

To test for trade-offs between life history traits and cancer defenses, we performed correlations between life history traits, such as body mass, lifespan and incubation length, versus neoplasia and cancer prevalence. Because many life history traits are correlated (Supplementary Fig. S11), we tested a series of multivariable regression models to control for those correlations. We performed a PGLS univariate analysis where body mass times lifespan was the independent variable, a PGLS bivariate analysis where both incubation length and body mass were the independent variables, and a PGLS multivariate analysis where incubation length, body mass, and clutch size were the independent variables. We found no significant association between body mass and neoplasia prevalence (75 species; 3124 necropsies) or malignancy prevalence (67 species; an analysis which had a different number of significant outlier species that we removed; 2786 necropsies) (Fig. 2; Supplementary Fig. S1; Supp lementary Table S2A). Second, there was no significant association between lifespan and neoplasia prevalence (51 species; 2665 necropsies) or malignancy prevalence (45 species; 2383 necropsies) (Fig. 3; Supp lementary Fig. 2; Supp lementary Table 2A). Third, there was no significant correlation between body mass times lifespan (an estimate of the total number of cell divisions in an animal) and neoplasia prevalence (36 species; 1829 necropsies) or malignancy prevalence (34 species; 1328 necropsies) (Supp lementary Fig. 3A; Supp lementary Fig. 3B: Supp lementary Table 2A). Fourth, there was no significant association between incubation length, when controlling for species body mass, and malignancy prevalence (Supp lementary Table 2A; 31 species and 1699 necropsies). Fifth, there was no significant association between incubation length, when controlling for both body mass and clutch size, and malignancy prevalence (Fig. 4; Supp lementary Table S2A; 30 species and 1665 necropsies). The above PGLS analyses do not have significant outliers. However, even when we included significant outliers in the univariate PGLS analyses, incubation length, body mass, lifespan, and body mass times lifespan were still not significantly correlated with neoplasia or malignancy prevalence (Supp lementary Fig. S12–14, 17–18).

**Figure 5.**
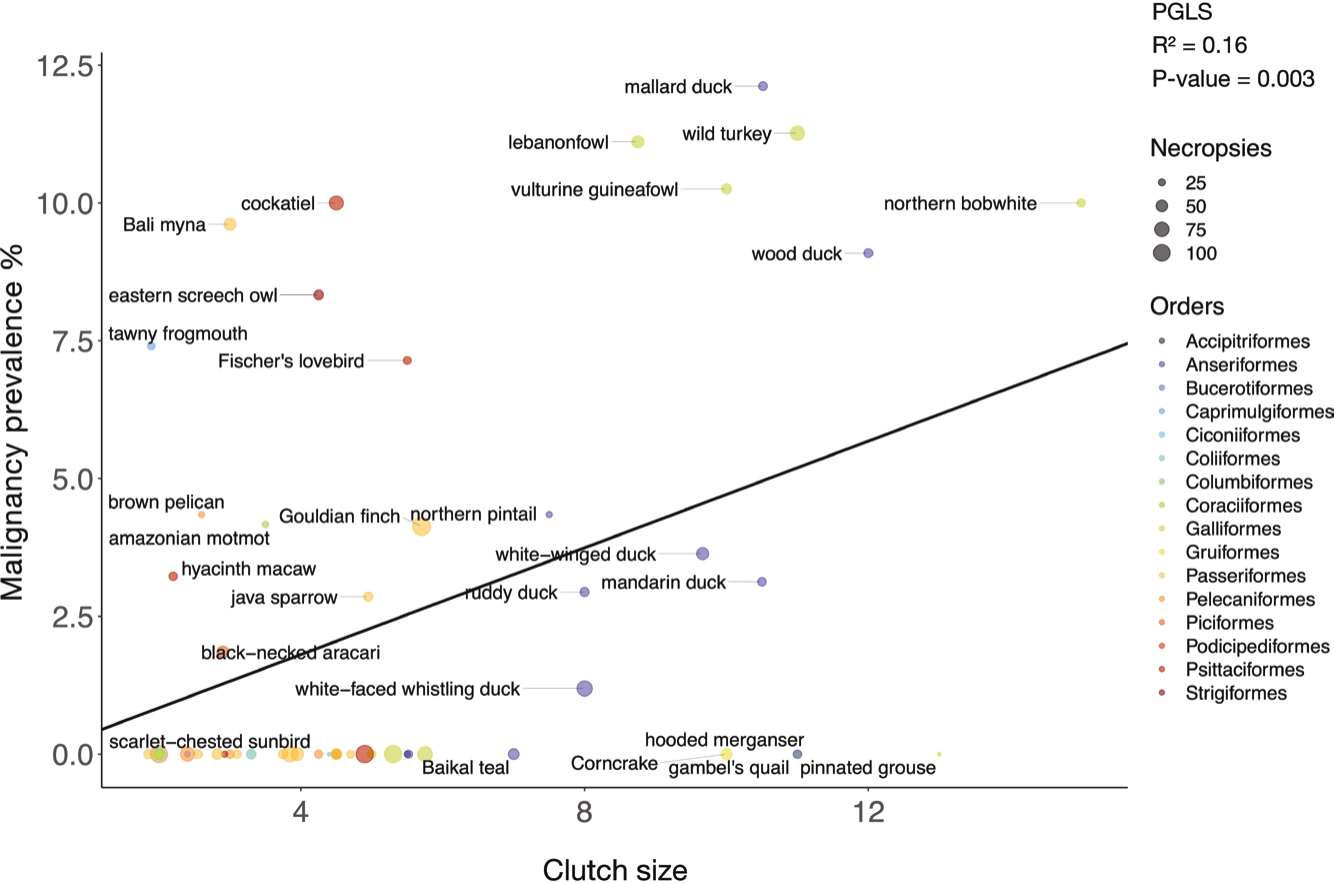
Clutch size is positively correlated with malignancy prevalence across 56 bird species. Dot size indicates the number of necropsies per species. Colors show the taxonomic order of each species. The black line shows the phylogenetically controlled linear regression of clutch size versus malignancy prevalence.

Species with larger clutch sizes had significantly higher malignancy prevalence (PGLS: *P*-value = 0.003; *R*² = 0.16, Fig. 5; 56 species and 2454 necropsies). This correlation was not significant after applying FDR corrections for multiple testing (FDR-corrected *P*-value = 0.08), nor after controlling for species body mass (FDR-corrected *P*-value = 0.11) (Supp lementary Table S2A). The correlation between clutch size and malignancy prevalence was not significant after removing domesticated and semi-domesticated species (PGLS: *P*-value = 0.38; 41 species and 1765 necropsies; Supp lementary Table S2A; Supp lementary Fig. S6B). Clutch size was not significantly correlated with neoplasia prevalence Supp lementary Table S2A; Supp lementary Fig. S5; Supp lementary Fig. S6A; 47–58 species, 1979–2955 necropsies). The above clutch size PGLS analyses did not include significant outliers. When including significant outliers in the analyses (Supp lementary Fig. S12–18), larger clutch size is still correlated with malignancy prevalence (Supp lementary Fig. S16B). This is true when including domesticated and semi-domesticated species (Supp lementary Fig. S16B; PGLS, *P*-value = 0.001) as well as when excluding domesticated and semi-domesticated species (Supp lementary Fig. S17B; PGLS *P*-value = 0.004). When including significant outliers in the analyses, neoplasia prevalence positively correlated with clutch size (Supp lementary Fig. S16A; PGLS *P*-value = 0.03).

To test whether older animals had more cancer than younger animals, we compared the age of animals that had or did not have cancer when they died. We found that animals with a diagnosis of cancer at death were not older on average than animals with a diagnosis of no cancer at death (in 1287 individuals from 51 species; for which we had age data) (Supp lementary Fig. S10).

To test for trade-offs between sexual dimorphism or dichromatism and cancer defenses, we looked for correlations between sexually dimorphic and dichromatic traits versus neoplasia and malignancy prevalence. Sexually dimorphic or dichromatic species with extreme phenotypes, such as large and colorful ornaments or weapons, may have an increased risk of cancer We found no significant associations between neoplasia or malignancy prevalence and several sexually dimorphic and dichromatic traits (Supp lementary Fig. S7; Supp lementary Table 2A). Also, in the 31 species for which we had at least 10 female and 10 male necropsies, there was no significant difference in neoplasia or malignancy prevalence between females and males (Supp lementary Fig. S8; Supp lementary Fig. S9; Supp lementary Table S2A).

## DISCUSSION

We hypothesized that the variation in species trade-offs between investment in reproduction versus somatic maintenance can explain some of the variation in cancer prevalence across bird species. We found that species with larger clutch sizes had more cancer in our dataset. The discovery adds to a growing evidence that links reproductive strategies to disease susceptibility in animals. However, other life-history traits that we tested, such as body mass, incubation length, lifespan, sexual size dimorphism, or sexual dichromatism, were not correlated with avian cancer prevalence, nor was there a significant difference in cancer or neoplasia prevalence between male and female birds.

We also found that cancer susceptibility in birds appears to have evolved under stabilizing selection with occasional shifts to different stable values. These results suggest that cancer prevalence and clutch size are correlated due to similar underlying selective pressures that are relatively stable, though they may shift with sudden changes in the ecologies of the birds. What those pressures were remains an open question for future research.

### Significant relationship between clutch size and cancer prevalence

Our results are consistent with previous findings in mammals that larger litter size is associated with cancer prevalence [12, 17]. Larger clutch size is correlated with malignancy prevalence across 56 bird species; a result that persisted in the majority (≥86%) of repetitions of these analyses using 40 randomly chosen bird species from our dataset (Supplementary material: Supplementary subsampling results). Many of the life-history traits described in this article, such as body mass, number of offspring produced per brood, incubation time, and longevity, are tightly linked with each other [61–65] (Supplementary Fig. S11). We found that clutch size explained a statistically significant portion (14–17%) of the variation in cancer prevalence when significant outliers were included in the analyses regardless of whether domesticated or semi-domesticated species were excluded. When significant outliers were excluded, clutch size also explained a statistically significant portion (16%) of the variation in cancer prevalence, though when excluding domesticated species from the analysis malignancy prevalence was not correlated with clutch size.

### Incubation length is not associated with cancer in birds

Incubation length is not correlated with cancer prevalence in birds even after controlling for body mass and clutch size. However, gestation or incubation length is negatively correlated with malignancy prevalence when controlling for variation in body mass across vertebrates [14].

### Evidence for Peto’s paradox in birds

Our findings show no significant correlation between neoplasia or malignancy prevalence and body mass and lifespan in birds, supporting Peto’s paradox [66]; the lack of relationship between body mass and neoplasia prevalence is in contrast to the observation of a positive correlation between body size and tumor prevalence in free-living birds [19]. Bulls et al. [20] found no significant correlation between neoplasia prevalence and body mass or lifespan in birds but found a significant negative correlation between cancer prevalence and lifespan in birds when using a threshold of ≥10 necropsies per species. The discrepancies between our study, that of Bulls et al. [20] and Møller et al. [19], may be due to the different number of individuals sampled per species (≥20 necropsies per species in our study, ≥5 and ≥10 necropsies in the correlations between life history traits and neoplasia or cancer prevalence in Bulls et al. [20] versus ≥3 records per species in Møller et al. [19]), the different species of birds analyzed (108 managed bird species from multiple institutions, 204 species [20] versus 238 free-living bird species in Denmark [19]), or body mass collected from the literature [20] (this study) versus mostly measured with a precision balance [19]. Unfortunately, only six species of birds are common in Møller et al’.s [19] and this study’s dataset, limiting our ability to compare cancer prevalence in wild versus managed birds. In general, patterns of tumor incidence or neoplasia prevalence were consistent between these free-living birds and populations managed under human care (Supplementary Table S3).

By analyzing the distribution of the age at which birds died, we found that birds with a diagnosis of cancer at death were not older than birds with no cancer found at death. This was also found in a larger taxonomic group (the sauropsids) that included birds [14]. This may be explained by the observation that long-lived birds have coevolved pathways that increase longevity in part through decreasing cancer rates [67, 68]. The fact that erythrocyte telomeres of long-lived birds shorten at a slower pace than erythrocyte telomeres of shorter-lived birds [69] may provide an additional mechanistic explanation for the lower than expected cancer prevalence in long-lived birds.

### No relationship between sexual dimorphism or dichromatism and cancer prevalence

We found no significant difference in cancer or neoplasia prevalence in relation to sexual dimorphism and dichromatism. This means that sexually dimorphic birds who spend time and energy in creating colorful plumage or larger body parts do not seem to pay a cost in terms of cancer susceptibility. It is possible that the birds in our study did not experience such trade-offs because under human care they may have high energy budgets that allow them to invest both in sexually selected traits as well as in somatic maintenance in the form of cancer suppression.

### Do female birds have higher cancer prevalence than male birds?

Cancer rates in most other species, including humans, are biased toward males [33]. Current theory hypothesizes that males, with a hemizygous sex chromosome, may be vulnerable to recessive cancer risk alleles on the X chromosome and that the double X chromosome found in females may offer some cancer protection against those recessive alleles [33]. In alignment with the two-X chromosome theory of cancer protection, previous work has shown that female birds (ZW) have more neoplasms than male birds (ZZ), but this was not validated statistically with sex-specific neoplasia prevalence [2]. We found that females do not have significantly different neoplasia prevalence or malignancy prevalence than male birds. This suggests that the sex chromosome hemizygosity of female birds does not increase their cancer risk. It is possible that female birds may get more cancer than males, but the effect size of hemizygosity is too small to be detected by a study of our size. So the lack of a statistically significant difference in our study may not be conclusive. It just puts in doubt the hemizygosity hypothesis for sex bias in cancer risk.

### Future directions

Future work investigating both the ultimate and proximate causes of cancer in birds would help us both understand cancer better and protect birds. What are the links between the number of oncogenes and tumor-suppressors across bird species and the Ornstein–Uhlenbeck model of stabilizing selection and random changes in bird cancer prevalence? Why are large clutch sizes a risk factor for cancer in birds? Several ecological factors may also be driving many of the cancers in birds in our dataset. Future studies would also benefit from knowledge of the relationships between distinct cancer types and life history in birds. Hormonal variation, the mechanisms that protect birds from radiation-induced DNA damage [70], as well as the molecular associations between unpredictable environments and fast life history strategies (e.g. production of more offspring) explaining cancer susceptibility across species would need to be found.

## Supplementary Material

eoae011_suppl_Supplementary_Data

## References

[1] Aktipis CA , Boddy AM, Jansen G et al. Cancer across the tree of life: cooperation and cheating in multicellularity. Philos Trans R Soc Lond B Biol Sci 2015;370:20140219–20140219. 26056363 10.1098/rstb.2014.0219 PMC4581024

[2] Effron M , Griner L, Benirschke K. Nature and rate of neoplasia found in captive wild mammals, birds, and reptiles at necropsy. J Natl Cancer Inst 1977;59:185–98. 577508 10.1093/jnci/59.1.185

[3] Aktipis CA , Nesse RM. Evolutionary foundations for cancer biology. Evol Appl 2013;6:144–59. 23396885 10.1111/eva.12034 PMC3567479

[4] Varmus H. The new era in cancer research. Science 2006;312:1162–5. 16728627 10.1126/science.1126758

[5] Bernards R , Jaffee E, Joyce JA et al. A roadmap for the next decade in cancer research. Nature Cancer 2020;1:12–7. 35121845 10.1038/s43018-019-0015-9

[6] Charnov EL. Life History Invariants. UK: Oxford University Press. 2003.

[7] Ghalambor CK , Martin TE. Fecundity-survival trade-offs and parental risk-taking in birds. Science 2001;292:494–7. 11313493 10.1126/science.1059379

[8] Caulin AF , Maley CC. Peto’s Paradox: evolution’s prescription for cancer prevention. Trends Ecol Evol 2011;26:175–82. 21296451 10.1016/j.tree.2011.01.002 PMC3060950

[9] Tollis M , Boddy AM, Maley CC. Peto’s Paradox: how has evolution solved the problem of cancer prevention? BMC Biol 2017;15:60. 28705195 10.1186/s12915-017-0401-7 PMC5513346

[10] Roche B , Sprouffske K, Hbid H et al. Peto’s paradox revisited: theoretical evolutionary dynamics of cancer in wild populations. Evol Appl 2013;6:109–16. 23396800 10.1111/eva.12025 PMC3567476

[11] Vincze O , Colchero F, Lemaître J-F et al. Cancer risk across mammals. Nature 2021;601:263–7. DOI: 10.1038/s41586-021-04224-5 34937938 PMC8755536

[12] Boddy AM , Abegglen LM, Pessier AP et al. Lifetime cancer prevalence and life history traits in mammals. Evol. Med. Public Health 2020;2020:187–95. DOI: 10.1093/emph/eoaa015/5843791 33209304 PMC7652303

[13] Harris VK , Schiffman JD, Boddy AM. Chapter 7–evolution of cancer defense mechanisms across species. In Ujvari B, Roche B, Thomas F (eds.). Ecology and Evolution of Cancer. Netherlands: Academic Press, 2017, 99–110.

[14] Compton ZT , Harris V, Mellon W et al. Cancer prevalence across vertebrates. bioRxiv 2023. DOI: 10.1101/2023.02.15.527881

[15] Kapsetaki SE , Basile AJ, Compton ZT et al. The relationship between diet, plasma glucose, and cancer prevalence across vertebrates. bioRxiv 2023:2023.07.31.551378. DOI: 10.1101/2023.07.31.551378 PMC11904020 40074744

[16] Kapsetaki SE , Compton Z, Rupp SM et al. The ecology of cancer prevalence across species: cancer prevalence is highest in desert species and high trophic levels. bioRxiv 2022:2022.08.23.504890. DOI: 10.1101/2022.08.23.504890

[17] Abegglen LM , Caulin AF, Chan A et al. Potential mechanisms for cancer resistance in elephants and comparative cellular response to DNA damage in humans. JAMA 2015;314:1850–60. 26447779 10.1001/jama.2015.13134 PMC4858328

[18] Dujon AM , Vincze O, Lemaitre J-F et al. The effect of placentation type, litter size, lactation and gestation length on cancer risk in mammals. Proc Biol Sci 2023;290:20230940. 37357861 10.1098/rspb.2023.0940 PMC10291710

[19] Møller AP , Erritzøe J, Soler JJ. Life history, immunity, Peto’s paradox and tumours in birds. J Evol Biol 2017;30:960–7. 28252229 10.1111/jeb.13060

[20] Bulls S , Platner L, Ayub W et al. Cancer prevalence is remarkably low in turtles and is correlated with life history traits in birds, mammals, and squamates. bioRxiv 2022:2022:2022.07.12.499088. DOI: 10.1101/2022.07.12.499088

[21] Reece RL. Observations on naturally occurring neoplasms in birds in the state of Victoria, Australia. Avian Pathol 1992;21:3–32. 18670912 10.1080/03079459208418815

[22] Langohr IM , Garner MM, Kiupel M. Somatotroph pituitary tumors in budgerigars (*Melopsittacus undulatus*). Vet Pathol 2012;49:503–7. 21900544 10.1177/0300985811419530

[23] Speer B. Current Therapy in Avian Medicine and Surgery. USA: Elsevier Health Sciences, 2015.

[24] Malka S , Keirstead ND, Gancz AY et al. Ingluvial squamous cell carcinoma in a geriatric cockatiel (*Nymphicus hollandicus*). avms 2005;19:234–9.

[25] Stewart HL. Pulmonary cancer and adenomatosis in captive wild mammals and birds from the Philadelphia zoo. J Natl Cancer Inst 1966;36:117–38. 4285273

[26] Snyder RL , Ratcliffe HL. Primary lung cancers in birds and mammals of the Philadelphia zoo. Cancer Res 1966;26:514–8. 5948820

[27] Boddy AM , Kokko H, Breden F et al. Cancer susceptibility and reproductive trade-offs: a model of the evolution of cancer defences. Philos Trans R Soc Lond B Biol Sci 2015;370:20140220. DOI: 10.1098/rstb.2014.0220 26056364 PMC4581025

[28] Klaassen M. Moult and basal metabolic costs in males of two subspecies of stonechats: the European *Saxicola torquata* rubicula and the East African *S. t. axillaris*. Oecologia 1995;104:424–32. 28307657 10.1007/BF00341339

[29] Moreno J , Sanz J, Merino S et al. Daily energy expenditure and cell-mediated immunity in pied flycatchers while feeding nestlings: interaction with moult. Oecologia 2001;129:492–7. 24577688 10.1007/s004420100767

[30] Vézina F , Gustowska A, Jalvingh KM et al. Hormonal correlates and thermoregulatory consequences of molting on metabolic rate in a northerly wintering shorebird. Physiol Biochem Zool 2009;82:129–42. 19199554 10.1086/596512

[31] Cherel Y , Charrassin JB, Challet E. Energy and protein requirements for molt in the king penguin *Aptenodytes patagonicus*. Am J Physiol 1994;266:R1182–8. 8184961 10.1152/ajpregu.1994.266.4.R1182

[32] Xirocostas ZA , Everingham SE, Moles AT. The sex with the reduced sex chromosome dies earlier: a comparison across the tree of life. Biol Lett 2020;16:20190867. 32126186 10.1098/rsbl.2019.0867 PMC7115182

[33] Dorak MT , Karpuzoglu E. Gender differences in cancer susceptibility: an inadequately addressed issue. Front Genet 2012;3:268. 23226157 10.3389/fgene.2012.00268 PMC3508426

[34] World Association of Zoos and Aquariums. Towards sustainable population management, Vol. 12, Czech Republic, 2011.

[35] Kapsetaki S , Compton Z, Dolan J et al. Dataset of “Life history traits and cancer prevalence in birds” article. Zenodo. 2024. DOI: 10.5281/zenodo.11311870 PMC11297545 39099847

[36] de Magalhães JP , Costa J. A database of vertebrate longevity records and their relation to other life-history traits. J Evol Biol 2009;22:1770–4. 19522730 10.1111/j.1420-9101.2009.01783.x

[37] Myhrvold NP , Baldridge E, Chan B et al. An amniote life-history database to perform comparative analyses with birds, mammals, and reptiles. Ecology 2015;96:3109–000.

[38] Dunn PO , Armenta JK, Whittingham LA. Natural and sexual selection act on different axes of variation in avian plumage color. Sci Adv 2015;1:e1400155. 26601146 10.1126/sciadv.1400155 PMC4643820

[39] Lislevand T , Figuerola J, Székely T. Avian body sizes in relation to fecundity, mating system, display behavior, and resource sharing. Ecology 2007;88:1605–1605.

[40] Pal P , Starkweather KN, Hales KH et al. A review of principal studies on the development and treatment of epithelial ovarian cancer in the laying hen *Gallus gallus*. Comp Med 2021;71:271–84. 34325771 10.30802/AALAS-CM-20-000116 PMC8383999

[41] Kattner P , Strobel H, Khoshnevis N et al. Compare and contrast: pediatric cancer versus adult malignancies. Cancer Metastasis Rev 2019;38:673–82. 31832830 10.1007/s10555-019-09836-y

[42] Orlik YA. sparrow in hand is better the pigeon in the sky” About Birds of Colombia and South America. J Sci Educ Technol 2018;19:15–41.

[43] Ramírez Ayala EG. Simulación de un sistema productivo para suplir el mercado de mascotas del psitácido Aratinga Weddellii (lorito de cabeza gris) en la cuenca amazónica del Eduador. *Bachelor’s Thesis*. Universidad San Francisco de Quito, Quito, Ecuador 2007.

[44] Williams RB. Avian malaria: clinical and chemical pathology of *Plasmodium gallinaceum* in the domesticated fowl *Gallus gallus*. Avian Pathol 2005;34:29–47. 15763737 10.1080/03079450400025430

[45] Gillings S , Balmer DE, Caffrey BJ et al. Breeding and wintering bird distributions in Britain and Ireland from citizen science bird atlases. Glob Ecol Biogeogr 2019;28:866–74.

[46] Padilla-Jacobo G , Cano-Camacho H, López-Zavala R et al. Evolutionary history of Mexican domesticated and wild *Meleagris gallopavo*. Genet Sel Evol 2018;50:19. 29665772 10.1186/s12711-018-0388-8 PMC5905111

[47] Zann R , Runciman D. Primary sex ratios in zebra finches: no evidence for adaptive manipulation in wild and semi-domesticated populations. Behav Ecol Sociobiol 2003;54:294–302.

[48] Leli U. The gouldian finch: aviculture and reproduction. Watchbird 1992;19:31–35 + 48-49.

[49] Shen Q-K , Peng M-S, Adeola AC et al. Genomic analyses unveil helmeted guinea fowl (*Numida meleagris*) domestication in West Africa. Genome Biol Evol 2021;13:evab090. 34009300 10.1093/gbe/evab090 PMC8214406

[50] Forshaw JM. Parrots in profile the scarletchested parrot. watchbird 2001;28:4–5 + 7.

[51] Svanberg I. Towards a cultural history of the Bengalese Finch (*Lonchura domestica*). Zool Gart 2008;77:334–44.

[52] R Core Team. R: A language and environment for statistical computing. Vienna, Austria: R Foundation for Statistical Computing. 2015.

[53] Wickham H. GGPLOT2: Elegant Graphics for Data Analysis 2016. NY: Springer-Verlag, 2016.

[54] Wickham H , François R, Henry L et al. dplyr: a grammar of data manipulation. 2017. R package version 0 7 2018;8:4.

[55] Paradis E , Schliep K. ape 5.0: an environment for modern phylogenetics and evolutionary analyses in R. Bioinformatics 2019;35:526–8. 30016406 10.1093/bioinformatics/bty633

[56] Orme D , Freckleton R, Thomas G et al. Comparative analyses of phylogenetics and evolution in R. R package version 0 5 2013;2:1.

[57] Revell LJ. phytools: an R package for phylogenetic comparative biology (and other things). Methods Ecol Evol 2012;3:217–23. DOI: 10.1111/j.2041-210X.2011.00169.x

[58] Pennell MW , Eastman JM, Slater GJ et al. geiger v2.0: an expanded suite of methods for fitting macroevolutionary models to phylogenetic trees. Bioinformatics 2014;30:2216–8. 24728855 10.1093/bioinformatics/btu181

[59] Wickham H , Averick M, Bryan J et al. Welcome to the tidyverse. J Open Source Softw 2019;4:1686.

[60] Felsenstein J. Phylogenies and the comparative method. Am Nat 1985;125:1–15.

[61] Stearns SC. The Evolution of Life Histories. UK: OUP Oxford, 1992.

[62] Charnov EL. Life History Invariants: Some Explorations of Symmetry in Evolutionary Ecology. USA: Oxford University Press, 1993.

[63] Kihlström JE. Period of gestation and body weight in some placental mammals. Comp Biochem Physiol A Comp Physiol 1972;43:673–9. 4144141 10.1016/0300-9629(72)90254-x

[64] Clutton-Brock TH. The Evolution of Parental Care. Princeton, NJ: Princeton Univ. Press, 1991.

[65] West GB , Brown JH, Enquist BJ. Scaling in biology: patterns and processes, causes and consequences. Scaling in Biology 2000;87:112.

[66] Peto R , Roe FJ, Lee PN et al. Cancer and ageing in mice and men. Br J Cancer 1975;32:411–26. 1212409 10.1038/bjc.1975.242 PMC2024769

[67] Wirthlin M , Lima NCB, Guedes RLM et al. Parrot genomes and the evolution of heightened longevity and cognition. Curr Biol 2018;28:4001–8.e7. 30528582 10.1016/j.cub.2018.10.050 PMC6393663

[68] Roche B , Hochberg ME, Caulin AF et al. Natural resistance to cancers: a Darwinian hypothesis to explain Peto’s paradox. BMC Cancer 2012;12:387. DOI: 10.1186/1471-2407-12-387 22943484 PMC3488527

[69] Haussmann MF , Winkler DW, O’Reilly KM et al. Telomeres shorten more slowly in long-lived birds and mammals than in short-lived ones. Proc Biol Sci 2003;270:1387–92. 12965030 10.1098/rspb.2003.2385 PMC1691385

[70] Galván I , Bonisoli-Alquati A, Jenkinson S et al. Chronic exposure to low-dose radiation at Chernobyl favours adaptation to oxidative stress in birds. Funct Ecol 2014;28:1387–403.

