# Supplementary material for: Life history traits and cancer prevalence in birds
Source: Evol Med Public Health. 2024 Jun 27;12(1):105–16. doi: 10.1093/emph/eoae011 (PMC11297545; doi:10.1093/emph/eoae011)
Supplement: eoae011_suppl_Supplementary_Data [file eoae011_suppl_supplementary_data.zip › Supplementary Material.pdf]

Supplementary material

Supplementary subsampling results

Clutch size remained positively correlated with malignancy prevalence (PGLS: $P < 0.05$) in 8 out of 50 repetitions (= 16%) of the analysis using 5 randomly chosen species, in 16 out of 50 repetitions (= 32%) of the analysis using 10 randomly chosen species, in 25 out of 50 repetitions (= 50%) of the analysis using 20 randomly chosen species, and in 43 out of 50 repetitions (= 86%) of the analysis using 40 randomly chosen species from our dataset. Clutch size was positively correlated with neoplasia prevalence (PGLS: $P < 0.05$) in 4 out of 50 repetitions (= 8%) of the analysis using 5 randomly chosen species, in 7 out of 50 repetitions (= 14%) of the analysis using 10 randomly chosen species, in 4 out of 50 repetitions (= 8%) of the analysis using 20 randomly chosen species, and in 10 out of 50 repetitions (= 20%) of the analysis using 40 randomly chosen species from our dataset.

Supplementary figure legends

Supplementary Figure 1. Larger body mass is not correlated with neoplasia prevalence across 75 bird species. Dot size indicates the number of necropsies per species. Colors show the taxonomic order of each species, and black lines show the phylogenetically-controlled linear regression of body mass versus neoplasia prevalence.

Supplementary Figure 2. Longer lifespan is not correlated with neoplasia prevalence across 51 bird species. Dot size indicates the number of necropsies per species. Colors show the taxonomic order of each species. Black lines show the phylogenetically-controlled linear regression of species lifespan versus neoplasia prevalence.

Supplementary Figure 3. Body mass times lifespan is not correlated with neoplasia prevalence (36 species) (A) or malignancy prevalence (34 species) (B). The black lines show the phylogenetically-controlled linear regression of body mass times lifespan versus malignancy prevalence or neoplasia prevalence. Body mass is measured in grams, whereas lifespan is measured in months. Different colors show the different order each species belongs to.

Supplementary Figure 4. Incubation length is not correlated with neoplasia prevalence across 35 species. Different colors indicate the order in which each species belongs and the size of the dot indicates the number of necropsies per species. The black line shows the phylogenetically-controlled linear regression of incubation length versus neoplasia prevalence.

Supplementary Figure 5. Larger clutch size is not correlated with neoplasia prevalence across 58 bird species. Dot size indicates the number of necropsies per species. Colors show the taxonomic order of each species. The black line shows the phylogenetically-controlled linear regression of clutch size versus neoplasia prevalence.

Supplementary Figure 6. Larger clutch size is not correlated with neoplasia prevalence (47 species) (A) or malignancy prevalence (41 species) (B) after removing domesticated and semi-domesticated species from the analyses and performing multiple

testing corrections. After controlling for species body mass and multiple testing corrections, clutch size does not correlate with malignancy prevalence or neoplasia prevalence (Supp. Table 2). Dot size shows the number of necropsies per species. Colors show the taxonomic order of each species. Black lines indicate the phylogenetically-controlled linear regression of clutch size versus malignancy prevalence or neoplasia prevalence.

Supplementary Figure 7. Sexual dimorphic traits are not correlated with neoplasia or malignancy prevalence in birds. The degree of dimorphism in brightness is not correlated with neoplasia prevalence (A: 16 bird species) or malignancy prevalence (B: 15 bird species) when controlling for clutch size. The degree of dimorphism in hue is not correlated with neoplasia prevalence (C: 24 bird species) or malignancy prevalence (D: 23 bird species). The degree of dimorphism in mass is not correlated with neoplasia prevalence when controlling for clutch size across 29 species of birds (E) or malignancy prevalence across 40 species of birds (F). The degree of dimorphism in tail size is not correlated with neoplasia prevalence (G: 32 bird species) or malignancy prevalence (H: 24 bird species). A positive score on the x-axis indicates that the species has a relatively higher score in that trait in males than females, whereas a negative score on the x-axis shows that the species has a relatively higher score in that trait in females than males. Black lines show the phylogenetically-controlled linear regression of degree of dimorphism in the trait versus neoplasia prevalence or malignancy prevalence. Different colors indicate the order in which each species belongs and the size of the dot indicates the total number of necropsies per species.

Supplementary Figure 8. Neoplasia (A) and malignancy prevalence (B) are not significantly different between females and males across 31 bird species. Horizontal bars show the median neoplasia (A) or malignancy prevalence (B). We added minimal jitter for better visualization of individual data points.

Supplementary Figure 9. No significant sex bias in neoplasia (A) or malignancy prevalence (B) across 31 bird species. Each dot in plot A shows the male neoplasia prevalence and female neoplasia prevalence of a species. Whereas each dot in plot B shows the male malignancy prevalence and female malignancy prevalence of a species.

Supplementary Figure 10. Cancer deaths are not skewed towards old age. Normalized frequency of a species' age at death as a percentage of the species lifespan. Each density plot shows the necropsied individuals that had tumors (blue) and the necropsied individuals that did not have tumors (red). There are 1287 individuals in this distribution from which we have lifespan data.

Supplementary Figure 11. Pearson's correlation matrix with four life history variables shared by 33 species in our dataset (body mass, lifespan, incubation length, and clutch size). The X on the–0.09 coefficient value shows that this correlation is not statistically significant. The remaining correlations are statistically significant.

Supplementary Figure 12. When including significant outliers in the analyses, body mass is not correlated with neoplasia prevalence (A) or malignancy prevalence (B) (90 species). The black lines show the phylogenetically-controlled linear regression of body mass versus malignancy prevalence or neoplasia prevalence. Different colors show the different order each species belongs to.

Supplementary Figure 13. When including significant outlier in the analyses, lifespan is not correlated with neoplasia prevalence (A) or malignancy prevalence (B) (56 species). The black lines show the phylogenetically-controlled linear regression of lifespan versus malignancy prevalence or neoplasia prevalence. Different colors show the different order each species belongs to.

Supplementary Figure 14. When including significant outlier in the analyses, body mass times lifespan is not correlated with neoplasia prevalence (A) or malignancy prevalence (B) (53 species). The black lines show the phylogenetically-controlled linear regression of body mass times lifespan versus malignancy prevalence or neoplasia prevalence. Body mass is measured in grams, whereas lifespan is measured in months. Different colors show the different order each species belongs to.

Supplementary Figure 15. When including significant outliers in the analyses, incubation length is not correlated with malignancy prevalence across 35 species. Different colors indicate the order in which each species belongs and the size of the dot indicates the number of necropsies per species. The black line shows the phylogenetically-controlled linear regression of incubation length versus malignancy prevalence.

Supplementary Figure 16. When including significant outliers in the analyses, larger clutch size is correlated with malignancy prevalence (B) but not neoplasia prevalence (A) (59 species). Dot size shows the number of necropsies per species. Colors show the taxonomic order of each species. Black lines indicate the phylogenetically-controlled linear regression of clutch size versus malignancy prevalence or neoplasia prevalence.

Supplementary Figure 17. When including significant outliers and excluding domesticated and semi-domesticated species from the analyses, larger clutch size is correlated with malignancy prevalence (B) but not neoplasia prevalence (A) (50 species). Dot size shows the number of necropsies per species. Colors show the taxonomic order of each species. Black lines indicate the phylogenetically-controlled linear regression of clutch size versus malignancy prevalence or neoplasia prevalence.

Supplementary Figure 18. When including significant outliers in the analyses, sexual dimorphic traits are not correlated with neoplasia or malignancy prevalence. The degree of dimorphism in brightness or hue is not correlated with neoplasia prevalence (A, C) or malignancy prevalence (B, D) (25 species). The degree of dimorphism in mass is not correlated with neoplasia prevalence (E) or malignancy prevalence (F) (41 species). The degree of dimorphism in tail size is not correlated with neoplasia prevalence (G) or malignancy prevalence (H) (33 species). A positive score on the x-axis indicates that the species has a relatively higher score in that trait in males than females, whereas a negative score on the x-axis shows that the species has a relatively higher score in that trait in females than males. Black lines show the phylogenetically-controlled linear regression of degree of dimorphism in the trait versus neoplasia prevalence or malignancy prevalence. Different colors indicate the order in which each species belongs and the size of the dot indicates the total number of necropsies per species.

Supplementary Table 1. Species (A, B) with the highest and lowest malignancy prevalence and neoplasia prevalence.

A. Species with the highest neoplasia prevalence and lowest malignancy prevalence. This table includes 10 species with the highest neoplasia prevalence and lowest malignancy prevalence in our dataset (Supplementary data). Another 54 species in our dataset have 0% malignancy prevalence (Supplementary data).

**Species (common name)**	**↑ Neoplasia prevalence (necropsies)**	**Species (common name)**	**↓ Malignancy prevalence (necropsies)**
Platalea ajaja (roseate spoonbill)	29.03% (31)	*Spheniscus demersus* (African penguin)	0% (210)
Gallus gallus (chicken)	25.74% (272)	*Lophura edwardsi* (Edwards's pheasant)	0% (110)
Anas platyrhynchos (mallard duck)	21.21% (33)	*Agapornis nigrigenis* (black-cheeked lovebird)	0% (108)
Athene cunicularia (burrowing owl)	20.83% (24)	*Eudocimus ruber* (scarlet ibis)	0% (105)
Melopsittacus undulatus (budgerigar)	20.75% (477)	*Pitta sordida* (hooded pitta)	0% (89)
Numida meleagris (lebanonfowl)	16.67% (54)	*Rollulus rouloul* (crested partridge)	0% (80)
Aix sponsa (wood duck)	15.15% (33)	*Trichoglossus moluccanus* (rainbow lorikeet)	0% (80)
Netta rufina (red crested pochard)	15% (20)	*Theristicus melanopis* (black-faced ibis)	0% (72)
Rhea americana (greater rhea)	14.29% (21)	*Eos bornea* (red lory)	0% (60)
Agapornis fischeri (Fischer's lovebird)	14.29% (28)	*Copsychus malabaricus* (white-rumped shama)	0% (59)

B. Species with the highest malignancy prevalence and lowest neoplasia prevalence. This table includes 10 species with the highest malignancy prevalence and lowest neoplasia prevalence in our dataset (Supplementary data). Another 34 species in our dataset have 0% neoplasia prevalence (Supplementary data).

**Species (common name)**	**↑ Malignancy prevalence (necropsies)**	**Species (common name)**	**↓ Neoplasia prevalence (necropsies)**
Gallus gallus (chicken)	22.79% (272)	*Spheniscus demersus* (African penguin)	0% (210)
Melopsittacus undulatus (budgerigar)	17.40% (477)	*Lophura edwardsi* (Edwards's pheasant)	0% (110)
Athene cunicularia (burrowing owl)	16.67% (24)	*Pitta sordida* (hooded pitta)	0% (89)
Anas platyrhynchos (mallard duck)	12.12% (33)	*Rollulus rouloul* (crested partridge)	0% (80)
Meleagris gallopavo (wild turkey)	11.27% (71)	*Trichoglossus moluccanus* (rainbow lorikeet)	0% (80)
Numida meleagris (lebanonfowl)	11.11% (54)	*Theristicus melanopus* (black-faced ibis)	0% (72)
Acryllium vulturinum (vulturine guineafowl)	10.26% (39)	*Copsychus malabaricus* (white-rumped shama)	0% (59)
Nymphicus hollandicus (cockatiel)	10% (70)	*Chalcophaps indica* (common emerald dove)	0% (48)
Colinus virginianus (northern bobwhite)	10% (30)	*Ptilinopus superbus* (superb fruit dove)	0% (48)

Leucopsar rothschildi (Bali myna)	9.62% (52)	*Crex crex* (corncrake)	0% (47)

Supplementary Table 2. Summary statistics. We present the summary statistics of phylogenetic regressions (PGLS) between neoplasia and malignancy prevalence and life history variables, except for the comparison of neoplasia and malignancy prevalence in females and males for which we present the summary statistics of paired-samples sign tests. The number of species analyzed is different in the majority of analyses. This is due to the fact that not all life history variables are available for every species in the literature (**A** and **B**) and each analysis has a different number of significant outlier species (**A**). In the 1st P-value column we report the *P*-value of the first variable (i.e., variable A in the multivariate analysis) and its correlation coefficient, in the 2nd *P*-value column we report the *P*-value of variable B and its correlation coefficient, and in the 3rd *P*-value column we report the *P*-value of variable C and its correlation coefficient. In table **A**, we highlight the *P*-values that passed the False Discovery Rate (FDR) correction with an asterisk (*). In the F-statistics column we report the F-statistics of variable A, and in the "Type of Association" column we report the positive (+) or negative (–) correlation between the variable A and the prevalence of neoplasia or malignancy. High lambda values show that the associations are mainly explained by common ancestry.

A)

**Independent variable(s)**	**Figure**	**Dependent variable**	**R²**	**F-statistic and degrees of freedom (DF)**	**Lambda**	**Type of association**	***P*-value of variable A [correlation coefficient]**	***P*-value of variable B [correlation coefficient]**	***P*-value of variable C [correlation coefficient]**
body mass	Supp. Fig. 1	asin(sqrt(Neoplasia prevalence))	0.01	1.01 on 1 and 73 DF	0.00007	+	0.32 [0.00004]	NA	NA
	2	asin(sqrt(Malignancy prevalence))	0.009	0.53 on 1 and 65 DF	0.00007	+	0.47 [0.00002]	NA	NA
lifespan	Supp. Fig. 2	asin(sqrt(Neoplasia prevalence))	–0.0002	0.006 on 1 and 49 DF	0.00007	–	0.94 [–0.00001]	NA	NA
	3	asin(sqrt(Malignancy prevalence))	0.004	0.70 on 1 and 43 DF	0.00007	–	0.41 [–0.00008]	NA	NA
body mass ▪ lifespan	Supp. Fig. 3A	asin(sqrt(Neoplasia prevalence))	0.03	1.03 on 1 and 34 DF	0.00007	–	0.32 [–0.0000005]	NA	NA

	Supp. Fig. 3B	asin(sqrt(Malignancy prevalence))	0.007	0.21 on 1 and 32 DF	0.00007	–	0.65 [–0.0000002]	NA	NA	
incubation length	Supp. Fig. 4	asin(sqrt(Neoplasia prevalence))	0.02	0.82 on 1 and 33 DF	0.00007	+	0.37 [0.11]	NA	NA	
incubation length + body mass		asin(sqrt(Malignancy prevalence))	0.07	2.20 on 1 and 28 DF	0.00007	+	0.17 [0.21]	0.83 [–0.00001]	NA	
incubation length + body mass + clutch size	4	asin(sqrt(Malignancy prevalence))	0.13	2.15 on 1 and 26 DF	0.00007	+	0.57 [0.10]	0.98 [0.000002]	0.19 [0.01]	
clutch size	Supp. Fig. 5	asin(sqrt(Neoplasia prevalence))	0.06	3.61 on 1 and 56 DF	0.00007	+	0.06 [0.01]	NA	NA	
	5	asin(sqrt(Malignancy prevalence))	0.16	10.10 on 1 and 54 DF	0.00007	+	0.003* [0.02]	NA	NA	
clutch size + body mass		asin(sqrt(Neoplasia prevalence)	0.06	3.16 on 1 and 49 DF	0.00007	+	0.11 [0.01]	0.65 [0.00002]	NA	

		)							
		asin(sqrt(Malignancy prevalence))	0.14	8.28 on 1 and 48 DF	0.00007	+	0.009 [0.02]	0.69 [0.00001]	NA
clutch size (having excluded domesticated and semi-domesticated species)	Supp. Fig. 6A	asin(sqrt(Neoplasia prevalence))	0.04	2.01 on 1 and 45 DF	0.00007	+	0.16 [0.008]	NA	NA
	Supp. Fig. 6B	asin(sqrt(Malignancy prevalence))	0.08	0.79 on 1 and 39 DF	1.00	+	0.38 [0.004]	NA	NA
clutch size + body mass (having excluded domesticated and semi-domesticated species)		asin(sqrt(Neoplasia prevalence))	0.06	2.61 on 1 and 40 DF	0.0000661	+	0.12 [0.01]	0.91 [–0.000006]	NA
		asin(sqrt(Malignancy prevalence))	0.11	0.31 on 1 and 34 DF	1.00	+	0.51 [0.003]	0.33 [0.00003]	NA
degree of dimorphism in brightness + clutch size	Supp. Fig. 7A	asin(sqrt(Neoplasia prevalence))	0.1	0.57 on 1 and 13 DF	0.55	+	0.86 [0.02]	0.40 [0.02]	NA

	Supp. Fig. 7B	asin(sqrt(Malignancy prevalence))	0.21	0.98 on 1 and 12 DF	0.00007	–	0.95 [–0.006]	0.17 [0.02]	NA
degree of dimorphism in hue	Supp. Fig. 7C	asin(sqrt(Neoplasia prevalence))	0.03	0.33 on 1 and 22 DF	0.19	–	0.57 [–0.02]	NA	NA
	Supp. Fig. 7D	asin(sqrt(Malignancy prevalence))	0.07	0.65 on 1 and 21 DF	0.51	–	0.43 [–0.02]	NA	NA
degree of dimorphism in mass + clutch size	Supp. Fig. 7E	asin(sqrt(Neoplasia prevalence))	0.15	0.77 on 1 and 26 DF	0.00007	–	0.30 [–1.37]	0.06 [0.01]	NA
degree of dimorphism in mass	Supp. Fig. 7F	asin(sqrt(Malignancy prevalence))	0.10	0.01 on 1 and 38 DF	0.30	+	0.92 [0.11]	NA	NA
degree of dimorphism in tail size	Supp. Fig. 7G	asin(sqrt(Neoplasia prevalence))	0.27	0.29 on 1 and 30 DF	0.62	–	0.59 [–1.33]	NA	NA
	Supp. Fig. 7H	asin(sqrt(Malignancy prevalence))	0.15	1.32 on 1 and 22 DF	0.92	–	0.26 [–1.49]	NA	NA

sex	Supp. Fig. 8A	asin(sqrt(Neoplasia prevalence))	95.9% CI = -0.09 – 0.03%	0.46	NA	NA
	Supp. Fig. 8B	asin(sqrt(Malignancy prevalence))	95.9% CI = 0 - 0.04%	0.31	NA	NA

B)

body mass	Supp. Fig. 12A	asin(sqrt(Neoplasia prevalence))	0.04	3.28 on 1 and 88 DF	0.00007	+	0.07 [0.00001]	NA	NA
	Supp. Fig. 12B	asin(sqrt(Malignancy prevalence))	0.06	3.37 on 1 and 88 DF	0.10	+	0.07 [0.00001]	NA	NA
lifespan	Supp. Fig. 13A	asin(sqrt(Neoplasia prevalence))	0.0005	0.03 on 1 and 54 DF	0.00007	+	0.87 [0.00002]	NA	NA
	Supp. Fig. 13B	asin(sqrt(Malignancy prevalence))	0.005	0.27 on 1 and 54 DF	0.00007	–	0.60 [–0.00006]	NA	NA
body mass ▪ lifespan	Supp. Fig. 14A	asin(sqrt(Neoplasia prevalence))	0.02	1.26 on 1 and 51 DF	0.00007	+	0.27 [0.00000001]	NA	NA
	Supp. Fig. 14B	asin(sqrt(Malignancy prevalence))	0.03	1.35 on 1 and 51 DF	0.00007	–	0.25 [0.00000001]	NA	NA
incubation length	Supp. Fig. 15	asin(sqrt(Malignancy prevalence)	0.06	1.90 on 1 and 33 DF	0.00007	+	0.18 [0.16]	NA	NA

		)							
clutch size	Supp. Fig. 16A	asin(sqrt(Neoplasia prevalence))	0.09	5.29 on 1 and 57 DF	0.00007	+	0.03* [0.01]	NA	NA
	Supp. Fig. 16B	asin(sqrt(Malignancy prevalence))	0.17	11.67 on 1 and 57 DF	0.00007	+	0.001* [0.01]	NA	NA
clutch size (having excluded domesticated and semi-domesticated species)	Supp. Fig. 17A	asin(sqrt(Neoplasia prevalence))	0.05	2.36 on 1 and 48 DF	0.00007	+	0.13 [0.01]	NA	NA
	Supp. Fig. 17B	asin(sqrt(Malignancy prevalence))	0.14	9.19 on 1 and 48 DF	0.00007	+	0.004* [0.02]	NA	NA
degree of dimorphism in brightness	Supp. Fig. 18A	asin(sqrt(Neoplasia prevalence))	0.05	0.04 on 1 and 23 DF	0.33	–	0.84 [–0.0004]	NA	NA
	Supp. Fig. 18B	asin(sqrt(Malignancy prevalence))	0.18	2.85 on 1 and 23 DF	0.68	–	0.11 [–0.003]	NA	NA

degree of dimorphism in hue	Supp. Fig. 18C	asin(sqrt(Neoplasia prevalence))	0.07	0.61 on 1 and 23 DF	0.27	+	0.44 [0.008]	NA	NA
	Supp. Fig. 18D	asin(sqrt(Malignancy prevalence))	0.17	2.20 on 1 and 23 DF	0.56	+	0.15 [0.01]	NA	NA
degree of dimorphism in mass	Supp. Fig. 18E	asin(sqrt(Neoplasia prevalence))	0.04	0.18 on 1 and 39 DF	0.13	–	0.67 [–0.50]	NA	NA
	Supp. Fig. 18F	asin(sqrt(Malignancy prevalence))	0.08	0.001 on 1 and 39 DF	0.24	–	0.97 [–0.04]	NA	NA
degree of dimorphism in tail size	Supp. Fig. 18G	asin(sqrt(Neoplasia prevalence))	0.3	1.26 on 1 and 31 DF	0.70	–	0.27 [–1.09]	NA	NA
	Supp. Fig. 18H	asin(sqrt(Malignancy prevalence))	0.3	0.03 on 1 and 31 DF	0.71	–	0.86 [–0.15]	NA	NA

Supplementary Table 3. Common species analyzed in Møller et al. (2017) and our study. Here we present species' tumor incidence and number of records in Møller et al.[19] versus neoplasia prevalence and number of necropsies in our study. We also present the *P*-values of Fisher's exact test.

**Species (common name)**	**Tumor incidence in Møller et al.'s study (# records)**	**Neoplasia prevalence in this study (# necropsies)**	***P*-value**
Columba livia (rock pigeon)	0% (3)	1.82% (55)	1
Anas acuta (northern pintail)	0% (3)	4.35% (23)	1
Milvus milvus (red kite)	0% (3)	0% (36)	1
Crex crex (corncrake)	0% (4)	0% (47)	1
Fringilla coelebs (chaffinch)	0% (213)	0% (45)	1
Anas platyrhynchos (mallard duck)	4.7% (21)	21.21% (33)	0.13
