# Supplementary material for: Life history traits and cancer prevalence in birds
Source: Evol Med Public Health. 2024 Jun 27;12(1):105–16. doi: 10.1093/emph/eoae011 (PMC11297545; doi:10.1093/emph/eoae011)
Supplement: eoae011_suppl_Supplementary_Data [file Supplementary Figures.pdf › Supplementary Figures.pdf]

Supp. Fig. 1

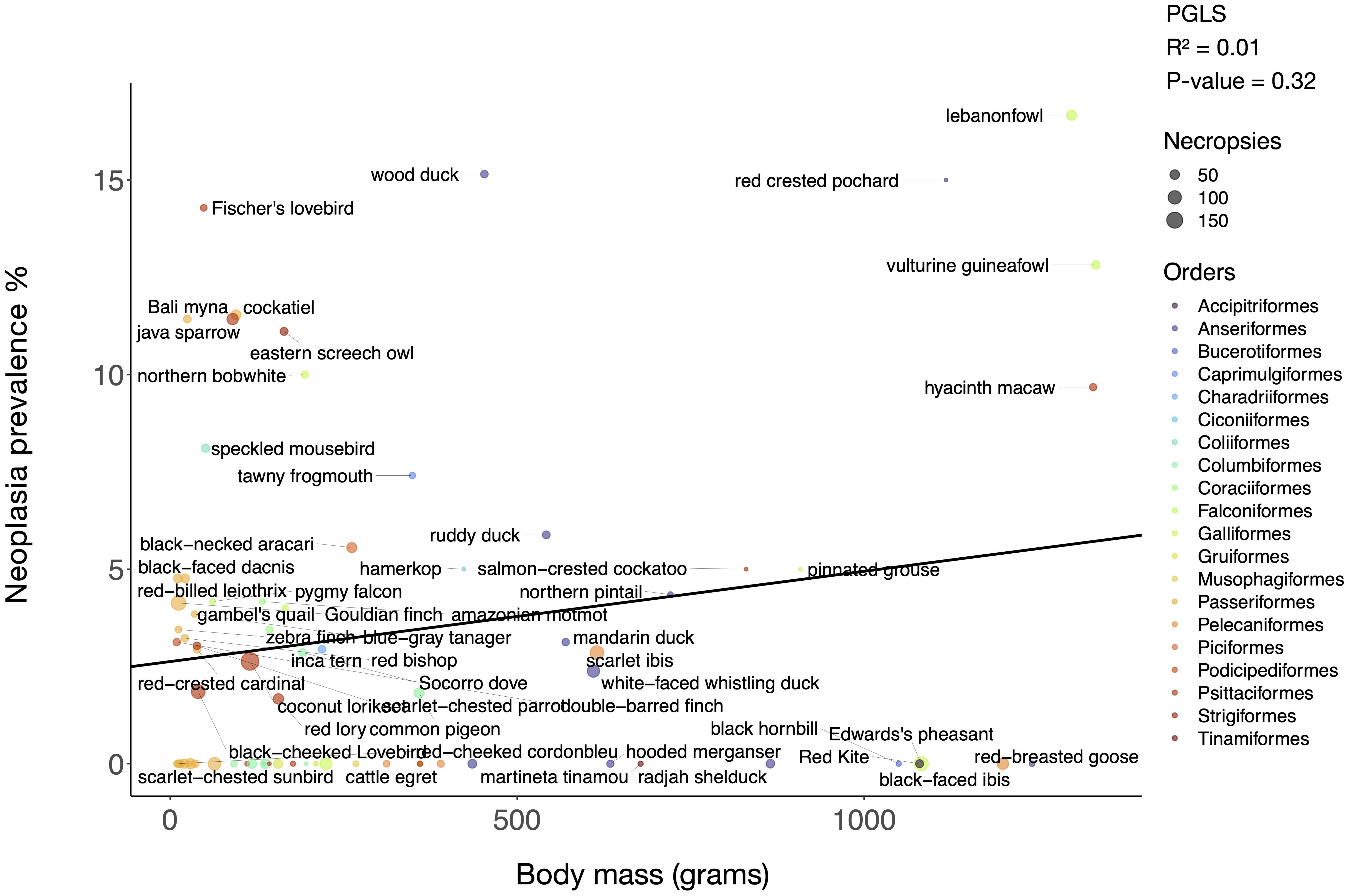
PGLS
R² = 0.01
P-value = 0.32
Necropsies
50
100
150
Orders
Accipitriformes
Anseriformes
Bucerotiformes
Caprimulgiformes
Charadriiformes
Ciconiiformes
Coliiformes
Columbiformes
Coraciiformes
Falconiformes
Galliformes
Gruiformes
Musophagiformes
Passeriformes
Pelecaniformes
Piciformes
Podicipediformes
Psittaciformes
Strigiformes
Tinamiformes
Neoplasia prevalence %
Body mass (grams)
0
5
10
15
0
500
1000
lebanonfowl
wood duck
red crested pochard
Fischer's lovebird
vulturine guineafowl
Bali myna
cockatiel
java sparrow
eastern screech owl
northern bobwhite
hyacinth macaw
speckled mousebird
tawny frogmouth
ruddy duck
black−necked aracari
black−faced dacnis
hamerkop
salmon−crested cockatoo
pinnated grouse
red−billed leiothrix
pygmy falcon
northern pintail
gambel's quail
Gouldian finch
amazonian motmot
zebra finch
blue−gray tanager
mandarin duck
inca tern
red bishop
scarlet ibis
red−crested cardinal
Socorro dove
white−faced whistling duck
coconut lorikeet
scarlet−chested parrot
double−barred finch
red lory
common pigeon
black hornbill
Edwards's pheasant
black−cheeked Lovebird
red−cheeked cordonbleu
hooded merganser
Red Kite
red−breasted goose
scarlet−chested sunbird
cattle egret
martineta tinamou
radjah shelduck
black−faced ibis

Supp. Fig. 2

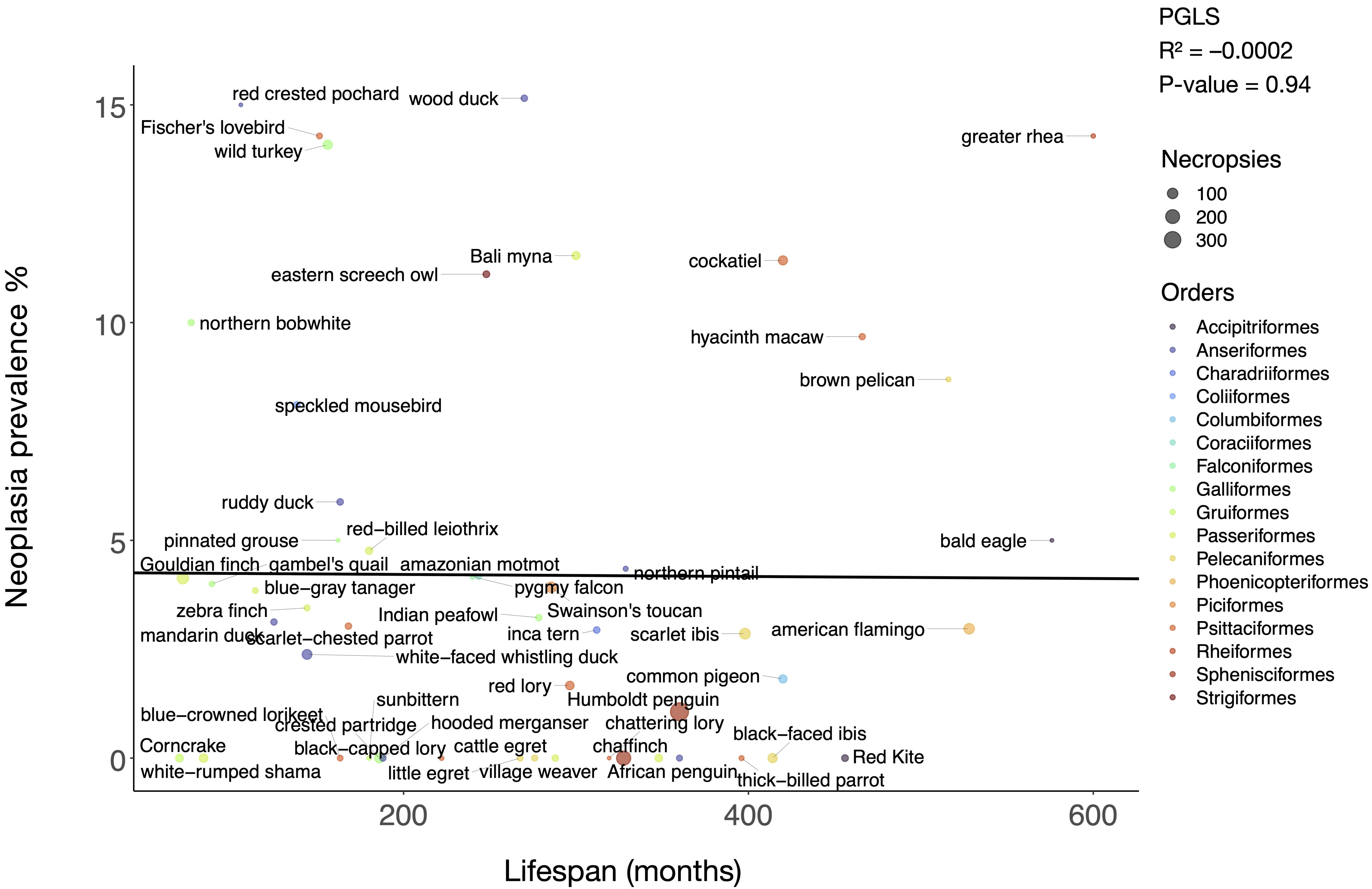
PGLS
R² = −0.0002
P-value = 0.94
Necropsies
100
200
300
Orders
Accipitriformes
Anseriformes
Charadriiformes
Coliiformes
Columbiformes
Coraciiformes
Falconiformes
Galliformes
Gruiformes
Passeriformes
Pelecaniformes
Phoenicopteriformes
Piciformes
Psittaciformes
Rheiformes
Sphenisciformes
Strigiformes
Neoplasia prevalence %
Lifespan (months)
0
5
10
15
200
400
600
red crested pochard
wood duck
Fischer's lovebird
wild turkey
greater rhea
Bali myna
eastern screech owl
cockatiel
northern bobwhite
hyacinth macaw
brown pelican
speckled mousebird
ruddy duck
red−billed leiothrix
pinnated grouse
bald eagle
Gouldian finch
gambel's quail
amazonian motmot
northern pintail
blue−gray tanager
pygmy falcon
zebra finch
Swainson's toucan
Indian peafowl
mandarin duck
scarlet−chested parrot
inca tern
scarlet ibis
american flamingo
white−faced whistling duck
common pigeon
red lory
Humboldt penguin
sunbittern
blue−crowned lorikeet
crested partridge
hooded merganser
chattering lory
black−faced ibis
Corncrake
black−capped lory
cattle egret
chaffinch
Red Kite
white−rumped shama
little egret
village weaver
African penguin
thick−billed parrot

Supp. Fig. 3

A

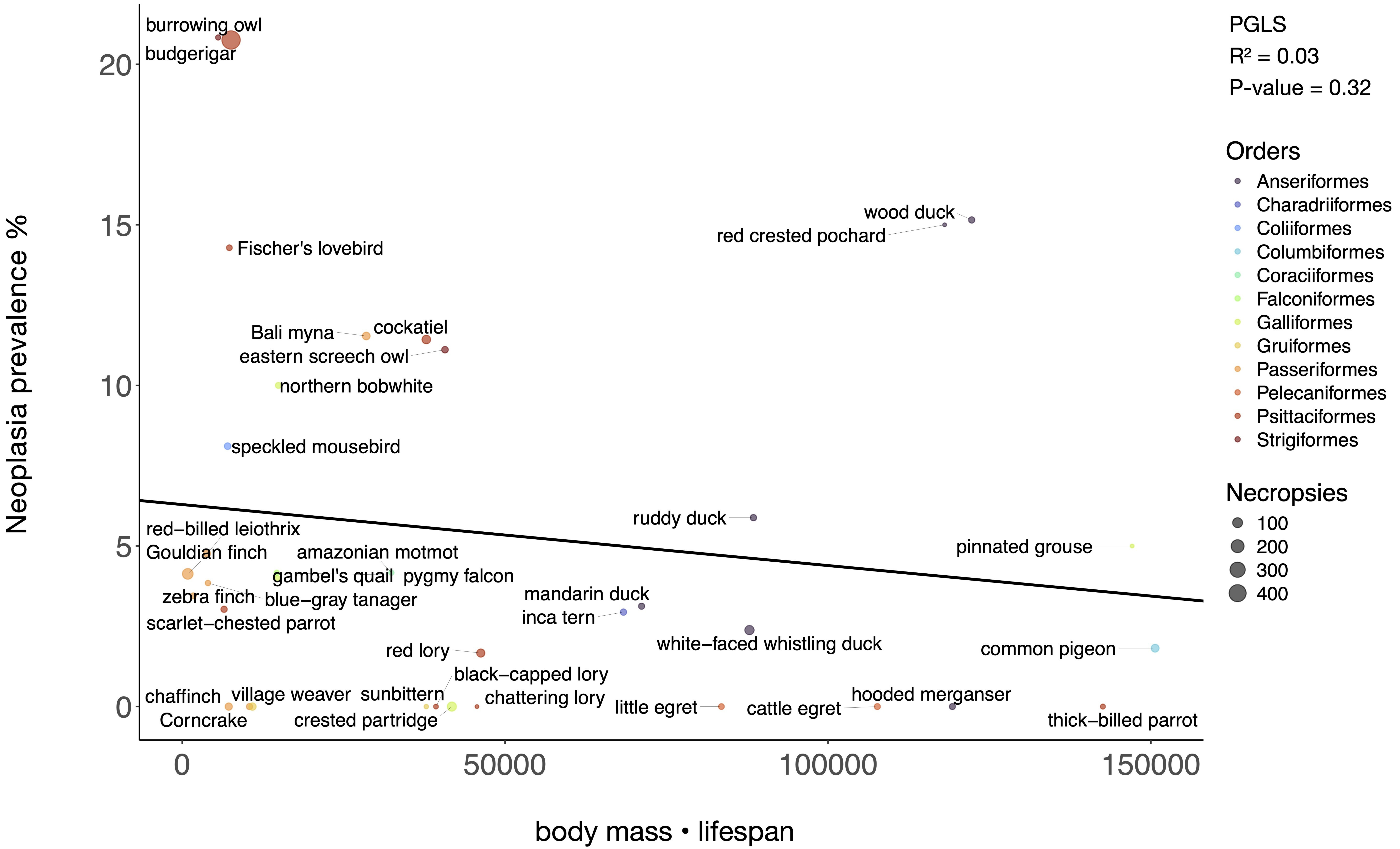

PGLS
R² = 0.03
P-value = 0.32
Orders
Anseriformes
Charadriiformes
Coliiformes
Columbiformes
Coraciiformes
Falconiformes
Galliformes
Gruiformes
Passeriformes
Pelecaniformes
Psittaciformes
Strigiformes
Necropsies
100
200
300
400
Neoplasia prevalence %
body mass • lifespan
0
50000
100000
150000
0
5
10
15
20
burrowing owl
budgerigar
Fischer's lovebird
Bali myna
cockatiel
eastern screech owl
northern bobwhite
speckled mousebird
wood duck
red crested pochard
ruddy duck
pinnated grouse
red–billed leiothrix
Gouldian finch
amazonian motmot
gambel's quail
pygmy falcon
zebra finch
blue–gray tanager
scarlet–chested parrot
mandarin duck
inca tern
white–faced whistling duck
common pigeon
red lory
black–capped lory
chaffinch
village weaver
sunbittern
chattering lory
little egret
cattle egret
hooded merganser
Corncrake
crested partridge
thick–billed parrot

Supp. Fig. 3

B

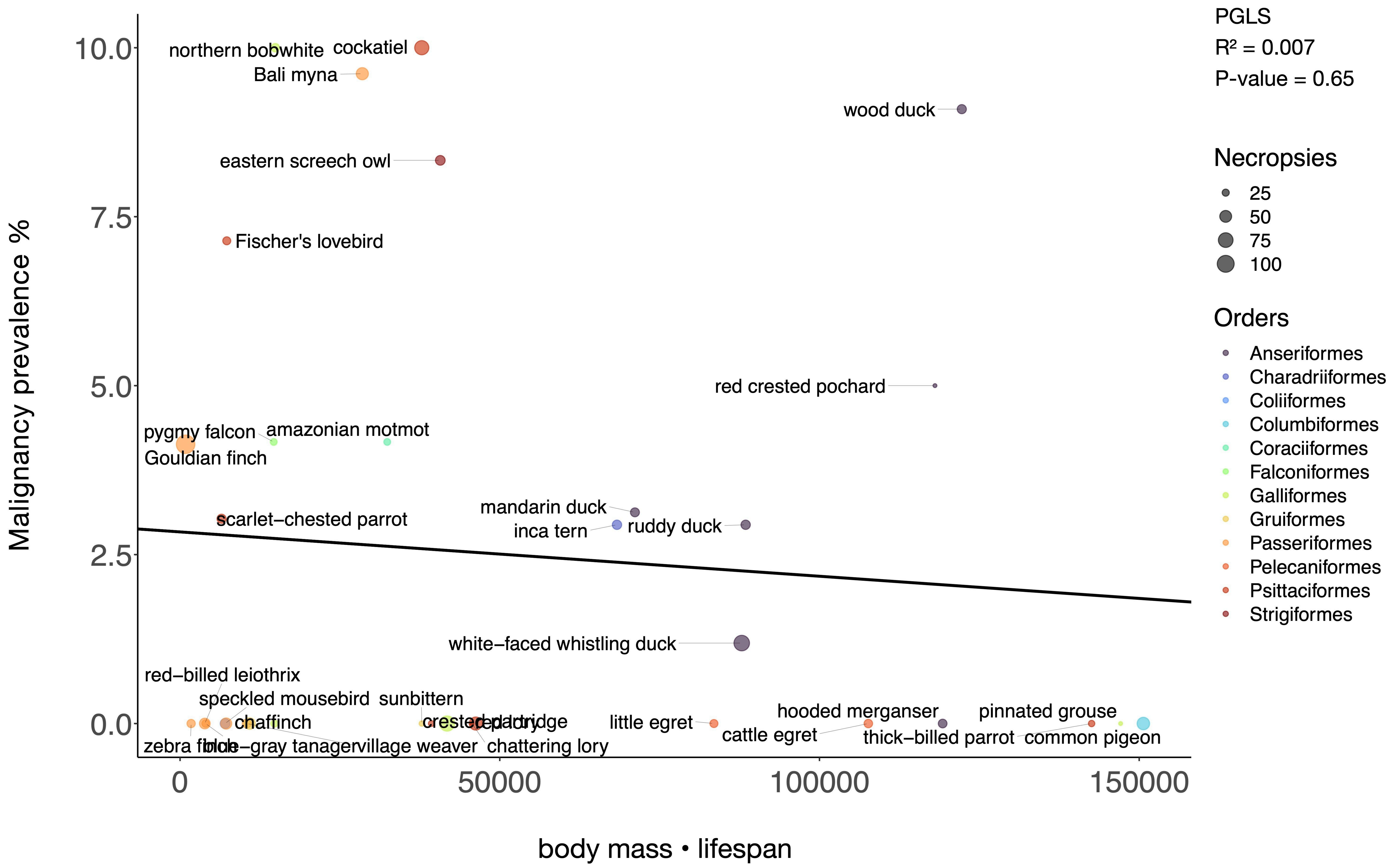
PGLS
R² = 0.007
P-value = 0.65
Necropsies
25
50
75
100
Orders
Anseriformes
Charadriiformes
Coliiformes
Columbiformes
Coraciiformes
Falconiformes
Galliformes
Gruiformes
Passeriformes
Pelecaniformes
Psittaciformes
Strigiformes
Malignancy prevalence %
body mass • lifespan
10.0
7.5
5.0
2.5
0.0
0
50000
100000
150000
northern bobwhite
cockatiel
Bali myna
wood duck
eastern screech owl
Fischer's lovebird
red crested pochard
pygmy falcon
amazonian motmot
Gouldian finch
mandarin duck
inca tern
ruddy duck
scarlet−chested parrot
white−faced whistling duck
red−billed leiothrix
speckled mousebird
sunbittern
chaffinch
little egret
hooded merganser
pinnated grouse
cattle egret
thick−billed parrot
common pigeon
village weaver
chattering lory

Supp. Fig. 4

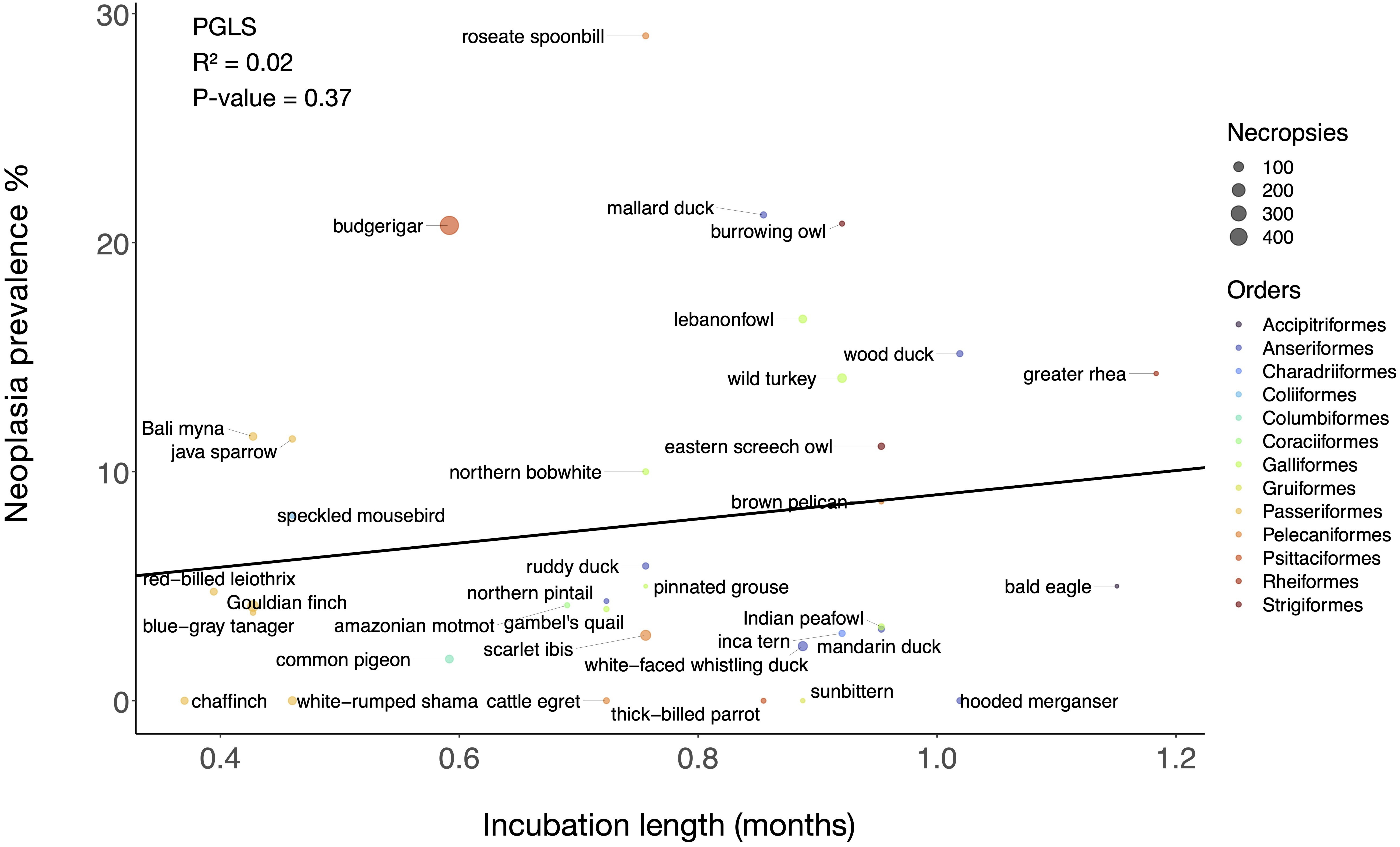
PGLS
R^2 = 0.02
P-value = 0.37
Neoplasia prevalence %
Incubation length (months)
0
10
20
30
0.4
0.6
0.8
1.0
1.2
roseate spoonbill
budgerigar
mallard duck
burrowing owl
lebanonfowl
wood duck
wild turkey
greater rhea
Bali myna
java sparrow
eastern screech owl
northern bobwhite
brown pelican
speckled mousebird
ruddy duck
red−billed leiothrix
pinnated grouse
bald eagle
northern pintail
Gouldian finch
blue−gray tanager
amazonian motmot
gambel's quail
Indian peafowl
inca tern
mandarin duck
scarlet ibis
common pigeon
white−faced whistling duck
sunbittern
chaffinch
white−rumped shama
cattle egret
thick−billed parrot
hooded merganser
Necropsies
100
200
300
400
Orders
Accipitriformes
Anseriformes
Charadriiformes
Coliiformes
Columbiformes
Coraciiformes
Galliformes
Gruiformes
Passeriformes
Pelecaniformes
Psittaciformes
Rheiformes
Strigiformes

Supp. Fig. 5

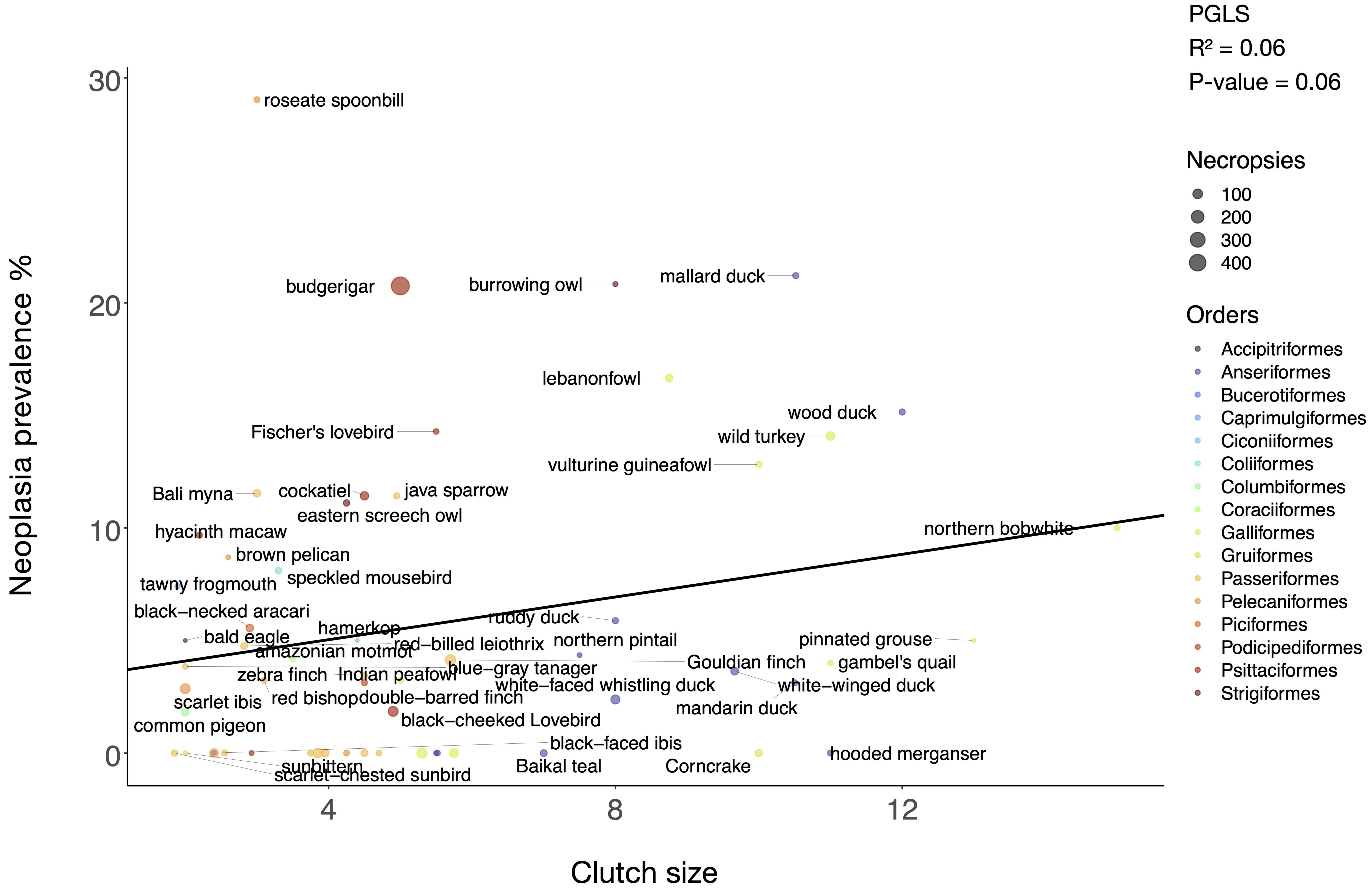

PGLS
R² = 0.06
P-value = 0.06
Necropsies
100
200
300
400
Orders
Accipitriformes
Anseriformes
Bucerotiformes
Caprimulgiformes
Ciconiiformes
Coliiformes
Columbiformes
Coraciiformes
Galliformes
Gruiformes
Passeriformes
Pelecaniformes
Piciformes
Podicipediformes
Psittaciformes
Strigiformes
Neoplasia prevalence %
Clutch size
roseate spoonbill
budgerigar
burrowing owl
mallard duck
lebanonfowl
wood duck
wild turkey
Fischer's lovebird
vulturine guineafowl
Bali myna
cockatiel
java sparrow
eastern screech owl
hyacinth macaw
brown pelican
speckled mousebird
tawny frogmouth
northern bobwhite
black–necked aracari
hamerkop
ruddy duck
bald eagle
red–billed leiothrix
northern pintail
amazonian motmot
pinnated grouse
Gouldian finch
gambel's quail
blue–gray tanager
zebra finch
Indian peafowl
white–faced whistling duck
white–winged duck
scarlet ibis
red bishop
double–barred finch
mandarin duck
common pigeon
black–cheeked Lovebird
black–faced ibis
hooded merganser
sunbittern
scarlet–chested sunbird
Baikal teal
Corncrake

Supp. Fig. 6

A

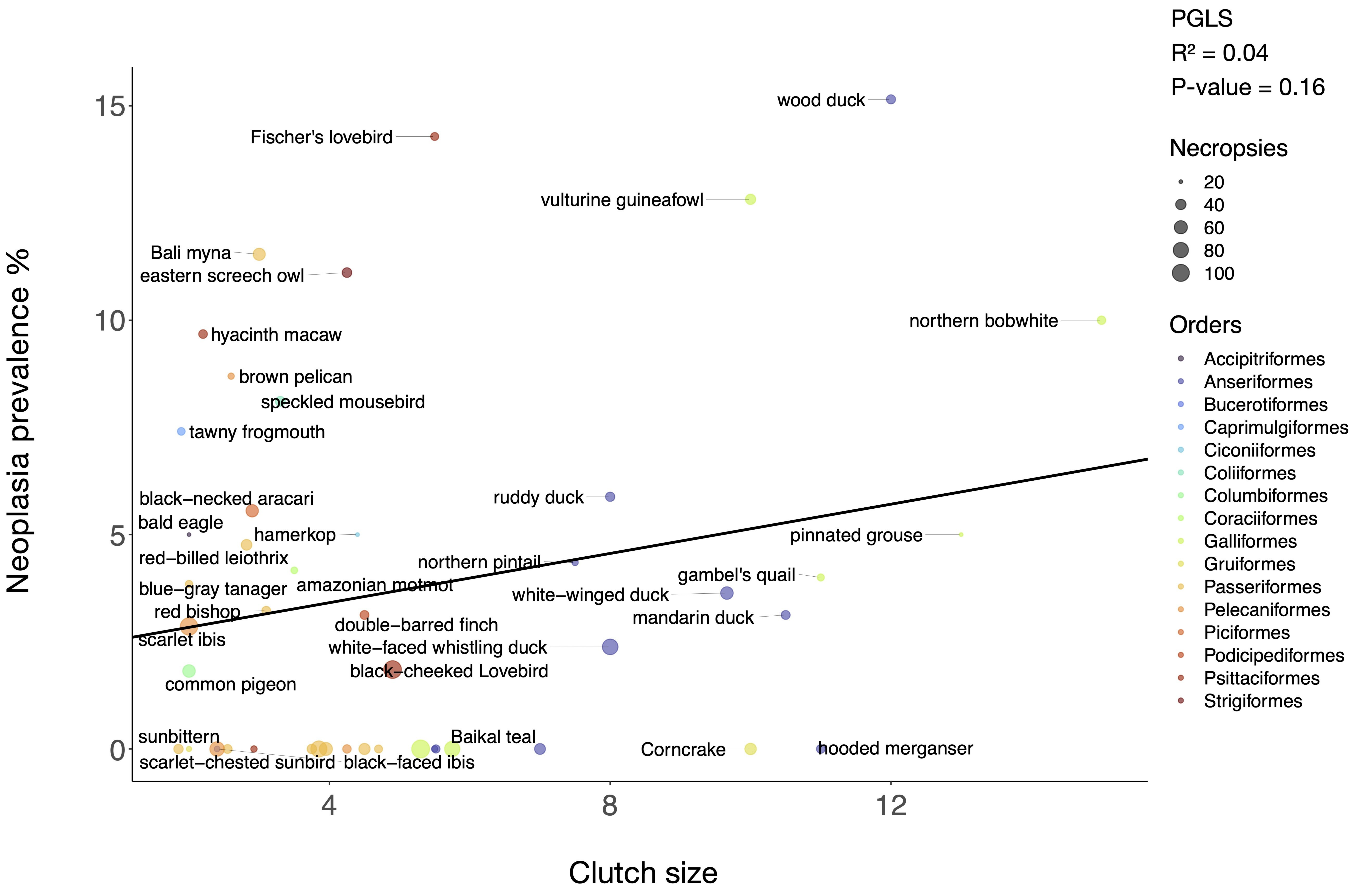
PGLS
R² = 0.04
P-value = 0.16
Necropsies
20
40
60
80
100
Orders
Accipitriformes
Anseriformes
Bucerotiformes
Caprimulgiformes
Ciconiiformes
Coliiformes
Columbiformes
Coraciiformes
Galliformes
Gruiformes
Passeriformes
Pelecaniformes
Piciformes
Podicipediformes
Psittaciformes
Strigiformes
Neoplasia prevalence %
Clutch size
15
10
5
0
4
8
12
wood duck
Fischer's lovebird
vulturine guineafowl
Bali myna
eastern screech owl
northern bobwhite
hyacinth macaw
brown pelican
speckled mousebird
tawny frogmouth
black−necked aracari
ruddy duck
bald eagle
hamerkop
pinnated grouse
red−billed leiothrix
northern pintail
gambel's quail
blue−gray tanager
amazonian motmot
white−winged duck
red bishop
mandarin duck
double−barred finch
scarlet ibis
white−faced whistling duck
black−cheeked Lovebird
common pigeon
sunbittern
Baikal teal
Corncrake
hooded merganser
scarlet−chested sunbird
black−faced ibis

Supp. Fig. 6

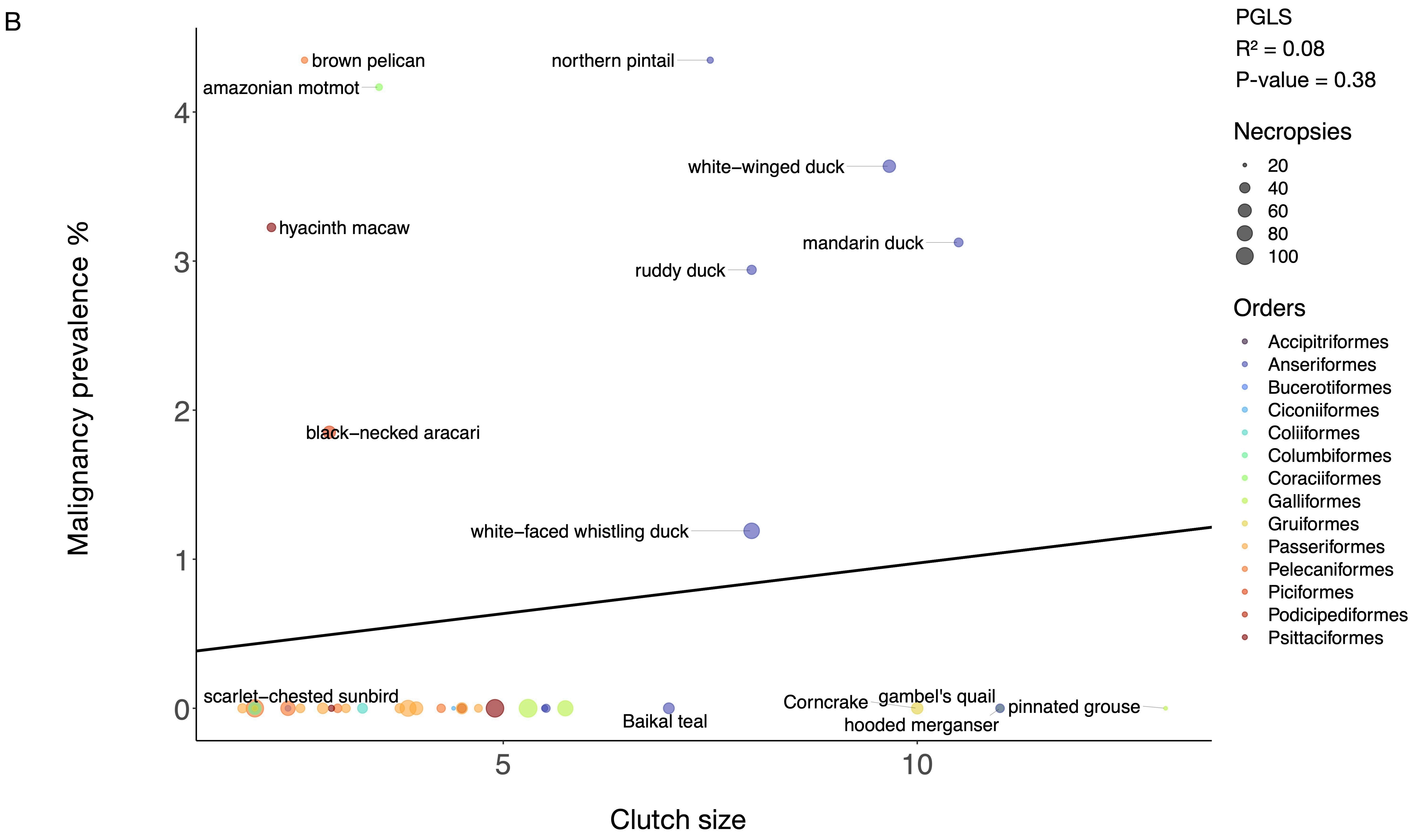
B
PGLS
R² = 0.08
P-value = 0.38
Necropsies
20
40
60
80
100
Orders
Accipitriformes
Anseriformes
Bucerotiformes
Ciconiiformes
Coliiformes
Columbiformes
Coraciiformes
Galliformes
Gruiformes
Passeriformes
Pelecaniformes
Piciformes
Podicipediformes
Psittaciformes
Malignancy prevalence %
Clutch size
brown pelican
northern pintail
amazonian motmot
white–winged duck
hyacinth macaw
mandarin duck
ruddy duck
black–necked aracari
white–faced whistling duck
scarlet–chested sunbird
Baikal teal
Corncrake
gambel's quail
pinnated grouse
hooded merganser
0
1
2
3
4
5
10

Supp. Fig. 7

A

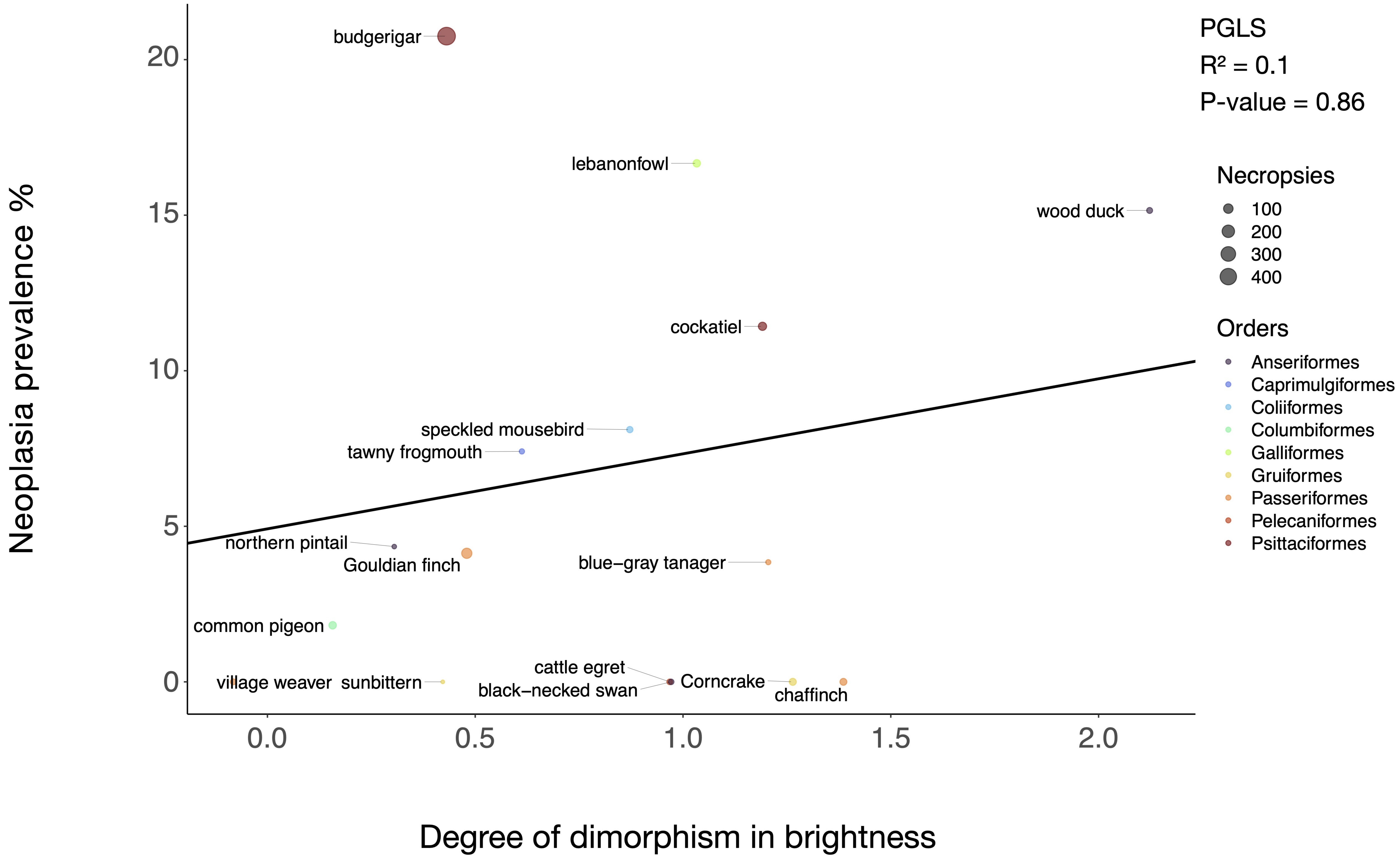

PGLS
R² = 0.1
P-value = 0.86
Neoplasia prevalence %
Degree of dimorphism in brightness
budgerigar
lebanonfowl
wood duck
cockatiel
speckled mousebird
tawny frogmouth
northern pintail
Gouldian finch
blue−gray tanager
common pigeon
cattle egret
village weaver
sunbittern
black−necked swan
Corncrake
chaffinch
Necropsies
100
200
300
400
Orders
Anseriformes
Caprimulgiformes
Coliiformes
Columbiformes
Galliformes
Gruiformes
Passeriformes
Pelecaniformes
Psittaciformes

Supp. Fig. 7

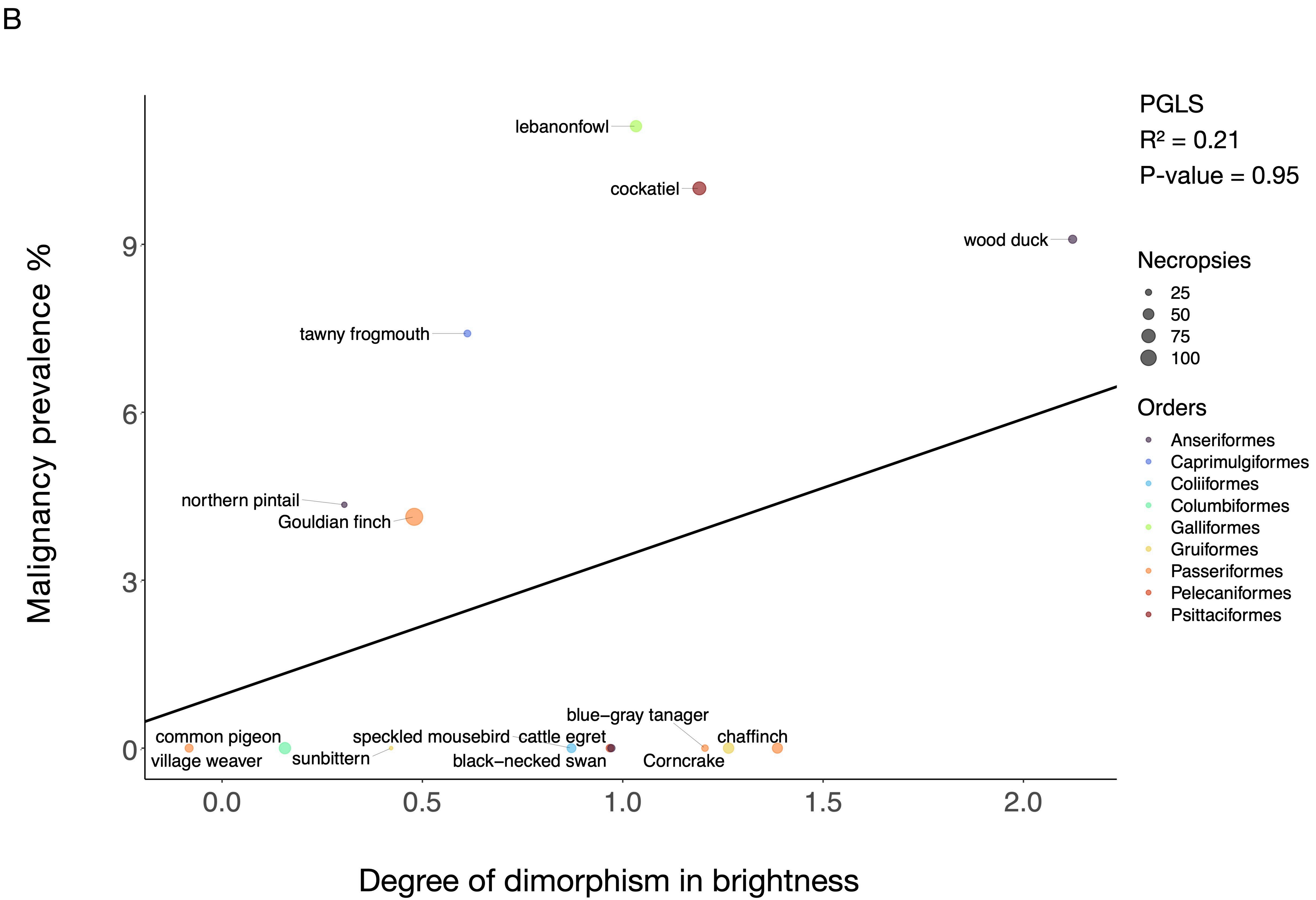
B
PGLS
R² = 0.21
P-value = 0.95
Necropsies
25
50
75
100
Orders
Anseriformes
Caprimulgiformes
Coliiformes
Columbiformes
Galliformes
Gruiformes
Passeriformes
Pelecaniformes
Psittaciformes
Malignancy prevalence %
Degree of dimorphism in brightness
0
3
6
9
0.0
0.5
1.0
1.5
2.0
lebanonfowl
cockatiel
wood duck
tawny frogmouth
northern pintail
Gouldian finch
blue−gray tanager
common pigeon
speckled mousebird
cattle egret
chaffinch
village weaver
sunbittern
black−necked swan
Corncrake

Supp. Fig. 7

C

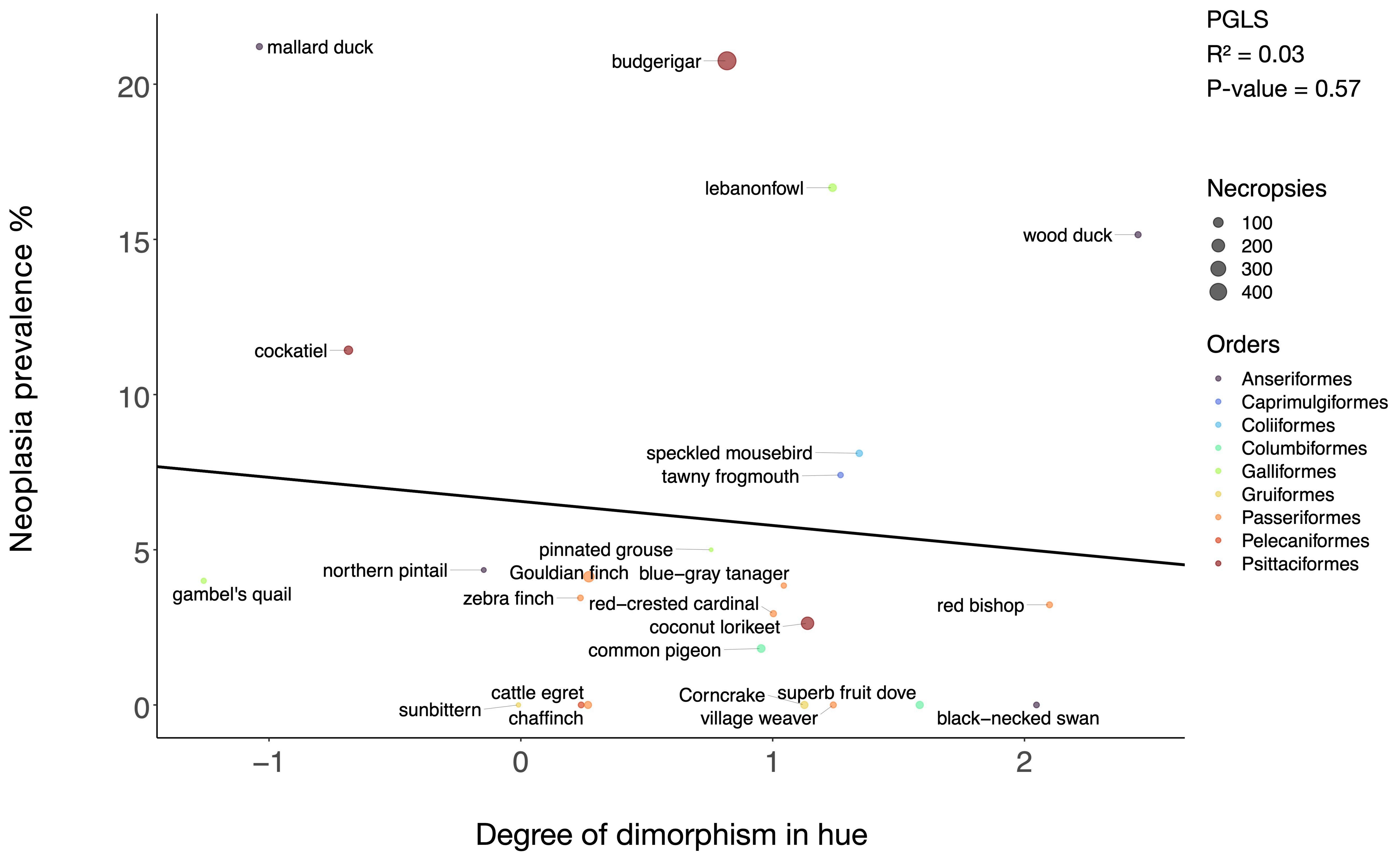

PGLS
R² = 0.03
P-value = 0.57
Necropsies
100
200
300
400
Orders
Anseriformes
Caprimulgiformes
Coliiformes
Columbiformes
Galliformes
Gruiformes
Passeriformes
Pelecaniformes
Psittaciformes
Neoplasia prevalence %
Degree of dimorphism in hue
20
15
10
5
0
−1
0
1
2
mallard duck
budgerigar
lebanonfowl
wood duck
cockatiel
speckled mousebird
tawny frogmouth
pinnated grouse
northern pintail
Gouldian finch
blue−gray tanager
gambel's quail
zebra finch
red−crested cardinal
red bishop
coconut lorikeet
common pigeon
cattle egret
sunbittern
chaffinch
Corncrake
superb fruit dove
village weaver
black−necked swan

Supp. Fig. 7

D

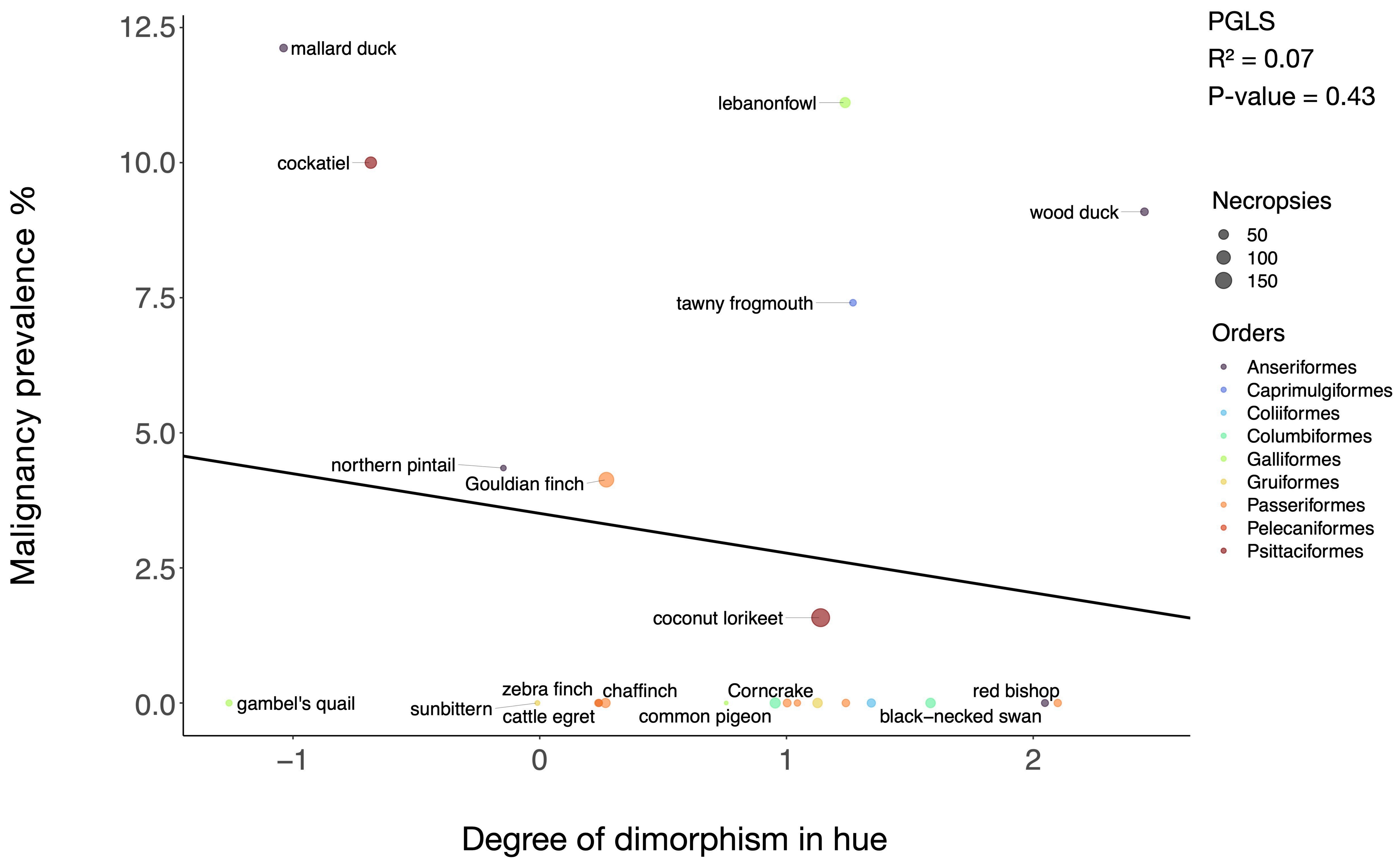

PGLS
R² = 0.07
P-value = 0.43
Necropsies
50
100
150
Orders
Anseriformes
Caprimulgiformes
Coliiformes
Columbiformes
Galliformes
Gruiformes
Passeriformes
Pelecaniformes
Psittaciformes
Malignancy prevalence %
Degree of dimorphism in hue
12.5
10.0
7.5
5.0
2.5
0.0
−1
0
1
2
mallard duck
lebanonfowl
cockatiel
wood duck
tawny frogmouth
northern pintail
Gouldian finch
coconut lorikeet
gambel's quail
zebra finch
sunbittern
cattle egret
chaffinch
common pigeon
Corncrake
red bishop
black−necked swan

Supp. Fig. 7

E

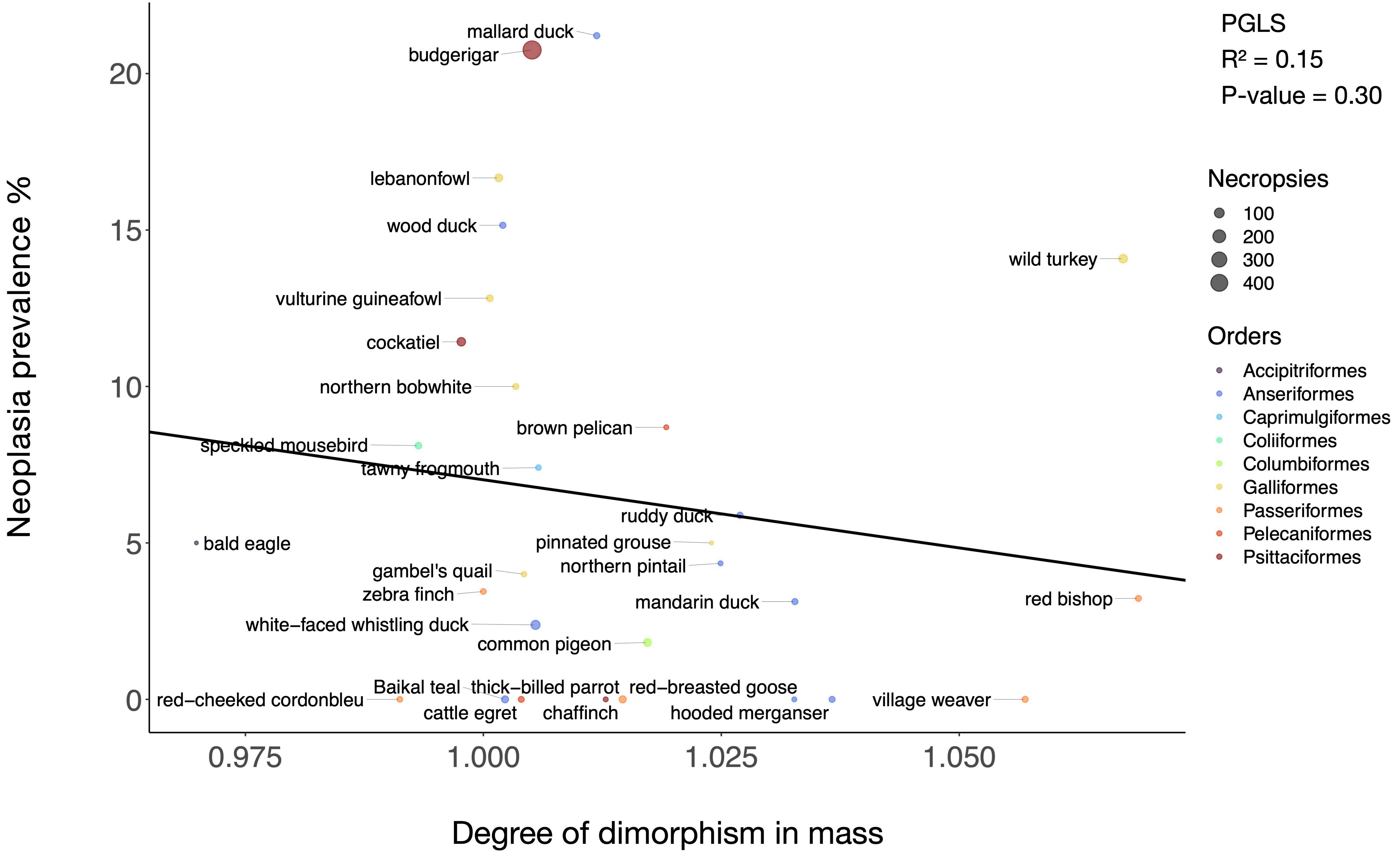

PGLS
R² = 0.15
P-value = 0.30
Necropsies
100
200
300
400
Orders
Accipitriformes
Anseriformes
Caprimulgiformes
Coliiformes
Columbiformes
Galliformes
Passeriformes
Pelecaniformes
Psittaciformes
mallard duck
budgerigar
lebanonfowl
wood duck
wild turkey
vulturine guineafowl
cockatiel
northern bobwhite
brown pelican
speckled mousebird
tawny frogmouth
ruddy duck
bald eagle
pinnated grouse
northern pintail
gambel's quail
zebra finch
mandarin duck
red bishop
white−faced whistling duck
common pigeon
Baikal teal
thick−billed parrot
red−breasted goose
red−cheeked cordonbleu
village weaver
cattle egret
chaffinch
hooded merganser
Neoplasia prevalence %
0
5
10
15
20
0.975
1.000
1.025
1.050
Degree of dimorphism in mass

Supp. Fig. 7

F

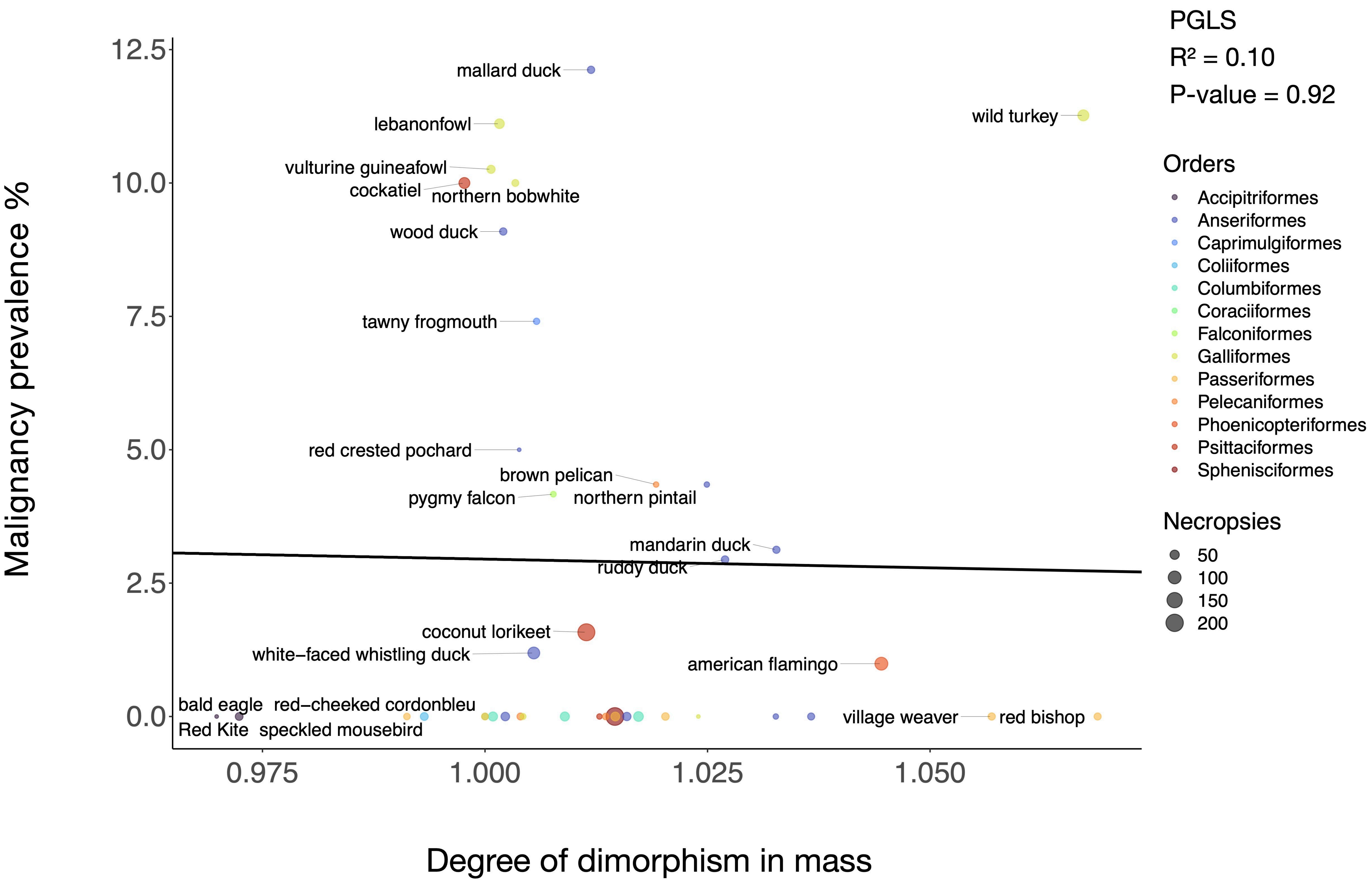

PGLS
R^2 = 0.10
P-value = 0.92
Malignancy prevalence %
Degree of dimorphism in mass
12.5
10.0
7.5
5.0
2.5
0.0
0.975
1.000
1.025
1.050
mallard duck
lebanonfowl
wild turkey
vulturine guineafowl
cockatiel
northern bobwhite
wood duck
tawny frogmouth
red crested pochard
brown pelican
pygmy falcon
northern pintail
mandarin duck
ruddy duck
coconut lorikeet
white−faced whistling duck
american flamingo
bald eagle
red−cheeked cordonbleu
Red Kite
speckled mousebird
village weaver
red bishop
Orders
Accipitriformes
Anseriformes
Caprimulgiformes
Coliiformes
Columbiformes
Coraciiformes
Falconiformes
Galliformes
Passeriformes
Pelecaniformes
Phoenicopteriformes
Psittaciformes
Sphenisciformes
Necropsies
50
100
150
200

Supp. Fig. 7

G

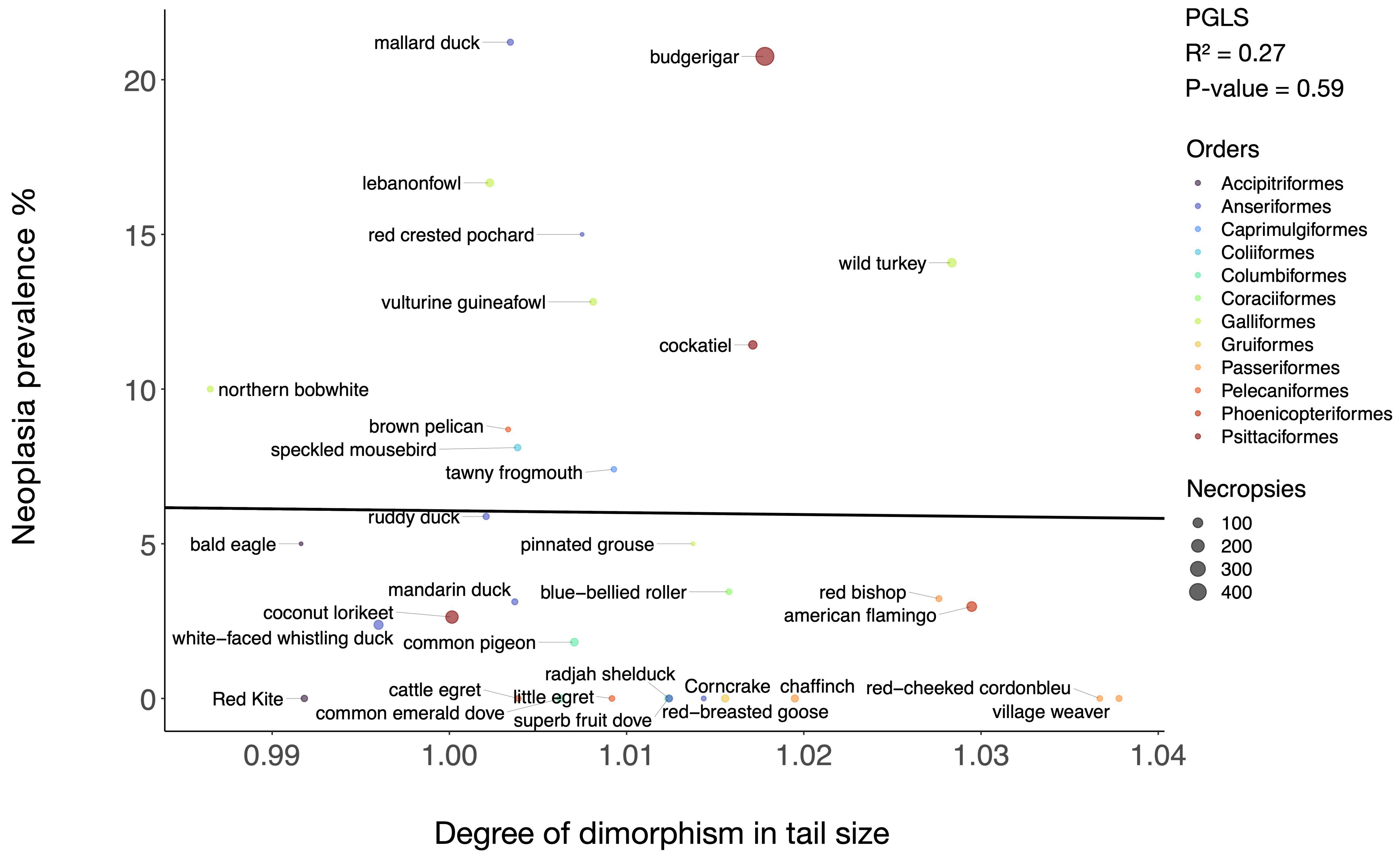
PGLS
R² = 0.27
P-value = 0.59
Orders
Accipitriformes
Anseriformes
Caprimulgiformes
Coliiformes
Columbiformes
Coraciiformes
Galliformes
Gruiformes
Passeriformes
Pelecaniformes
Phoenicopteriformes
Psittaciformes
Necropsies
100
200
300
400
Neoplasia prevalence %
Degree of dimorphism in tail size
0
5
10
15
20
0.99
1.00
1.01
1.02
1.03
1.04
mallard duck
budgerigar
lebanonfowl
red crested pochard
wild turkey
vulturine guineafowl
cockatiel
northern bobwhite
brown pelican
speckled mousebird
tawny frogmouth
ruddy duck
bald eagle
pinnated grouse
mandarin duck
blue−bellied roller
red bishop
american flamingo
coconut lorikeet
white−faced whistling duck
common pigeon
radjah shelduck
Corncrake
chaffinch
red−cheeked cordonbleu
cattle egret
little egret
Red Kite
common emerald dove
superb fruit dove
red−breasted goose
village weaver

Supp. Fig. 7

H

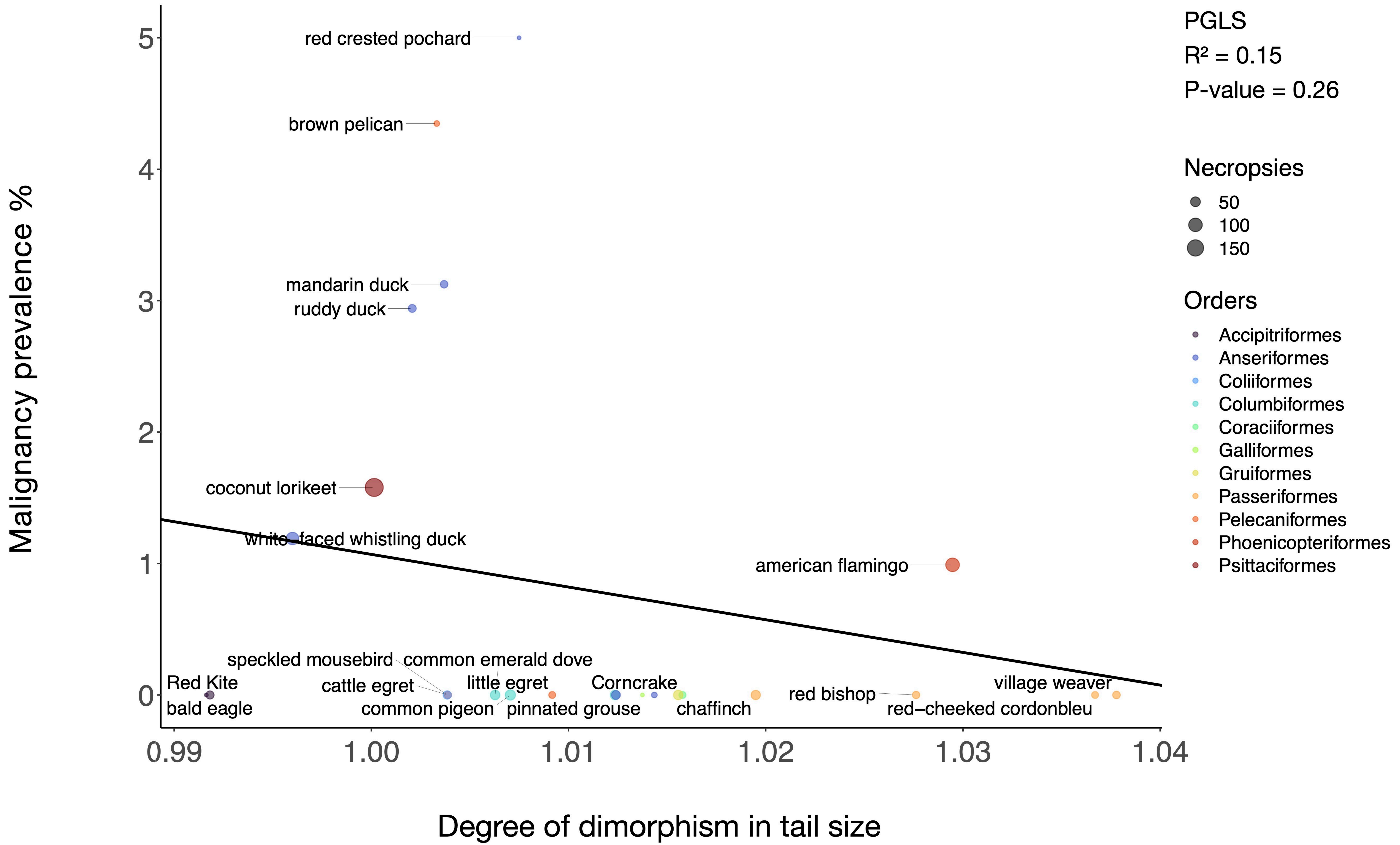

PGLS
R² = 0.15
P-value = 0.26
Necropsies
50
100
150
Orders
Accipitriformes
Anseriformes
Coliiformes
Columbiformes
Coraciiformes
Galliformes
Gruiformes
Passeriformes
Pelecaniformes
Phoenicopteriformes
Psittaciformes
Malignancy prevalence %
Degree of dimorphism in tail size
0.99
1.00
1.01
1.02
1.03
1.04
0
1
2
3
4
5
red crested pochard
brown pelican
mandarin duck
ruddy duck
coconut lorikeet
white-faced whistling duck
american flamingo
speckled mousebird
common emerald dove
Red Kite
cattle egret
little egret
Corncrake
village weaver
red bishop
bald eagle
common pigeon
pinnated grouse
chaffinch
red–cheeked cordonbleu

Supp. Fig. 8

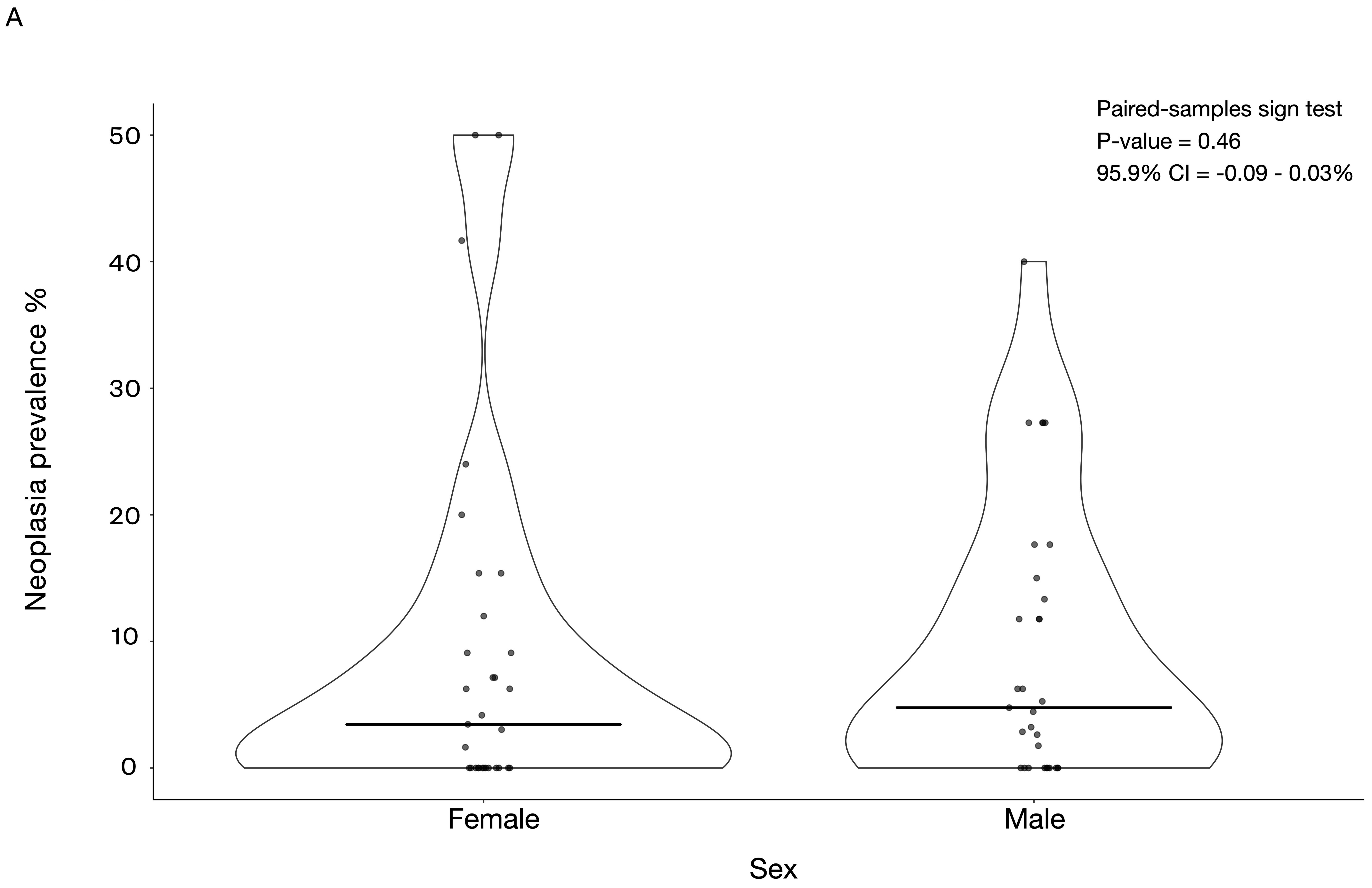
A
Paired-samples sign test
P-value = 0.46
95.9% CI = -0.09 - 0.03%
Neoplasia prevalence %
50
40
30
20
10
0
Female
Male
Sex

Supp. Fig. 8

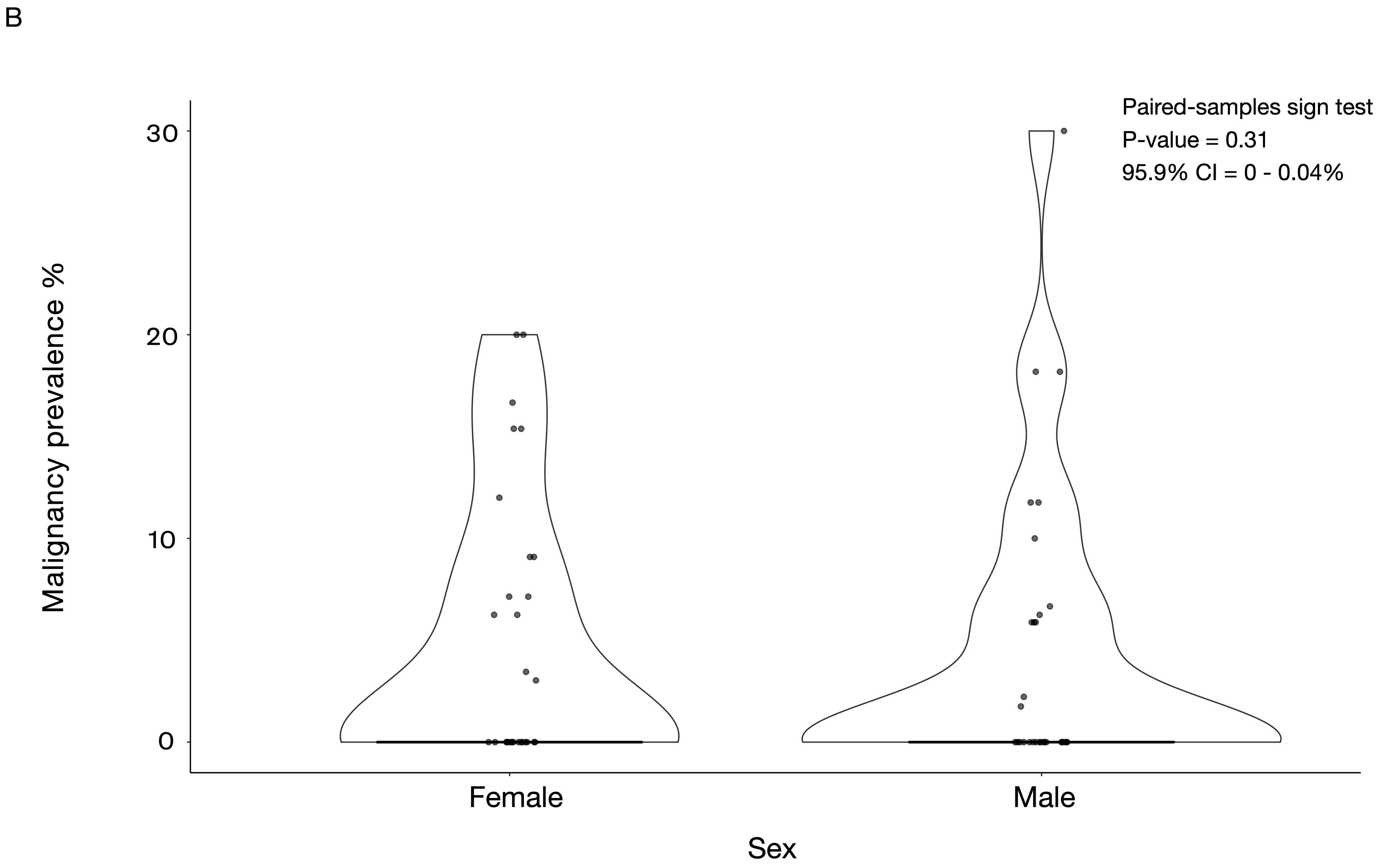
B
Paired-samples sign test
P-value = 0.31
95.9% CI = 0 - 0.04%
Malignancy prevalence %
30
20
10
0
Female
Male
Sex

Supp. Fig. 9

A

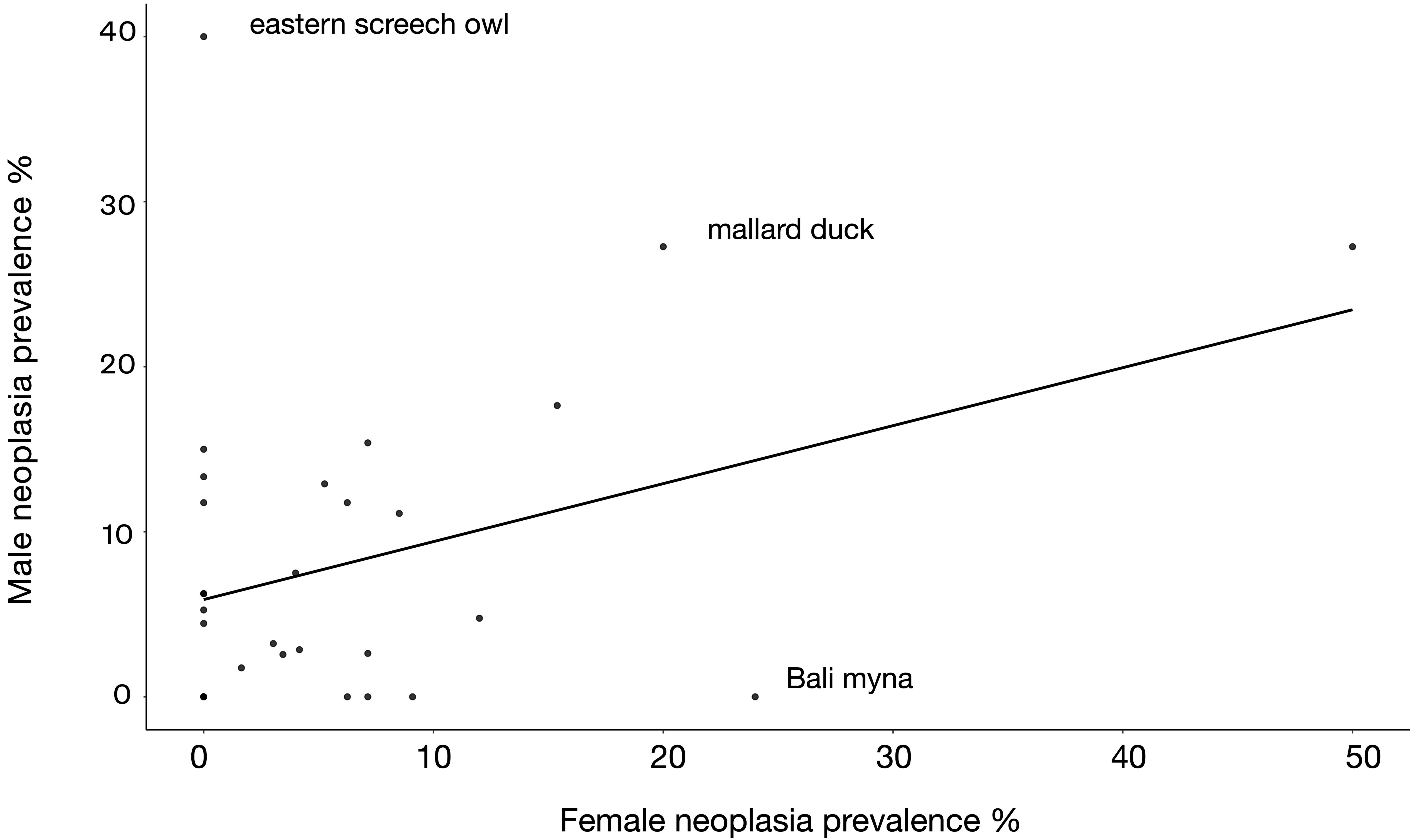

eastern screech owl
mallard duck
Bali myna
Male neoplasia prevalence %
Female neoplasia prevalence %
0
10
20
30
40
50

Supp. Fig. 9

B

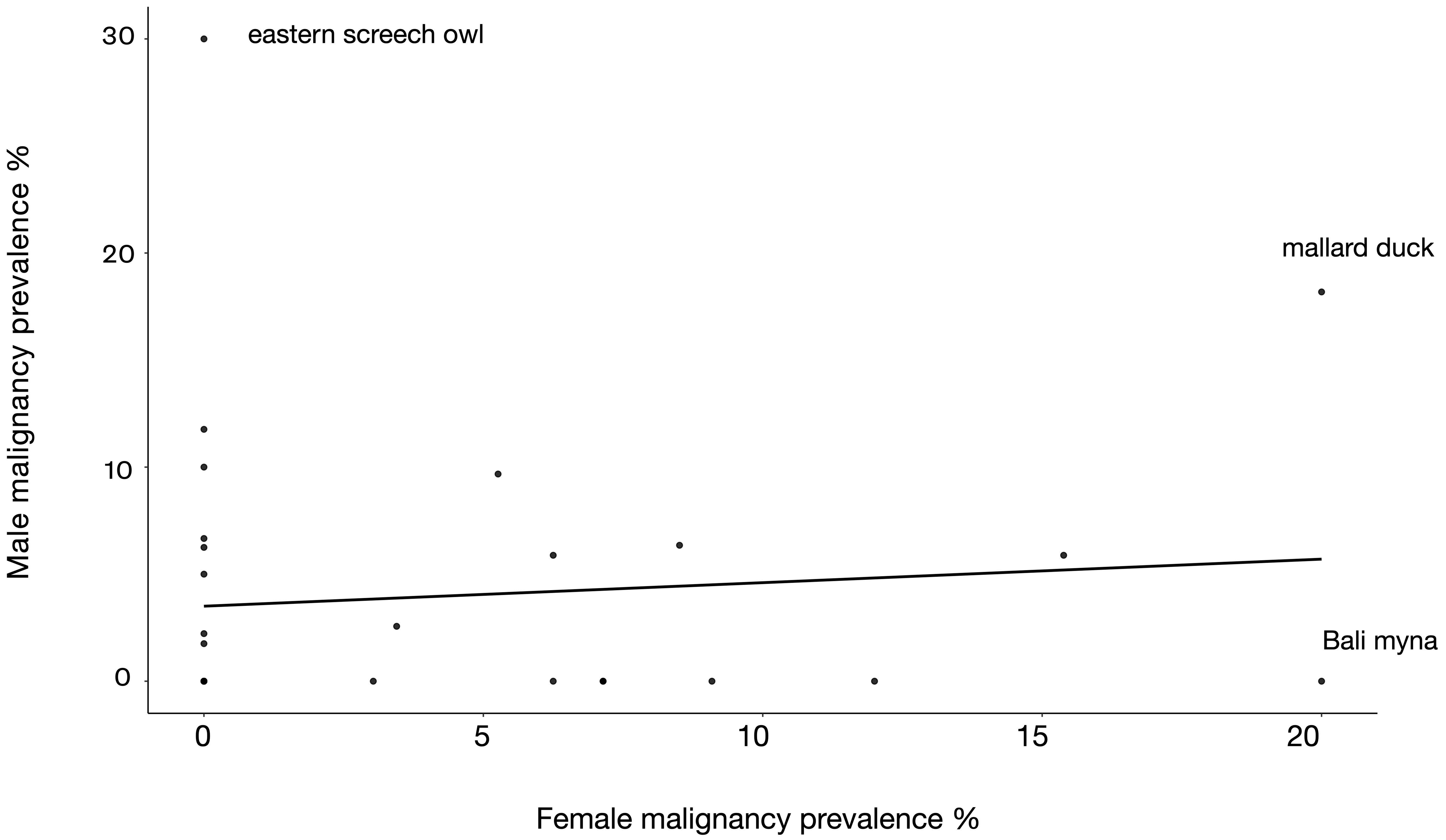

eastern screech owl
mallard duck
Bali myna
30
20
10
0
0
5
10
15
20
Male malignancy prevalence %
Female malignancy prevalence %

Supp. Fig. 10

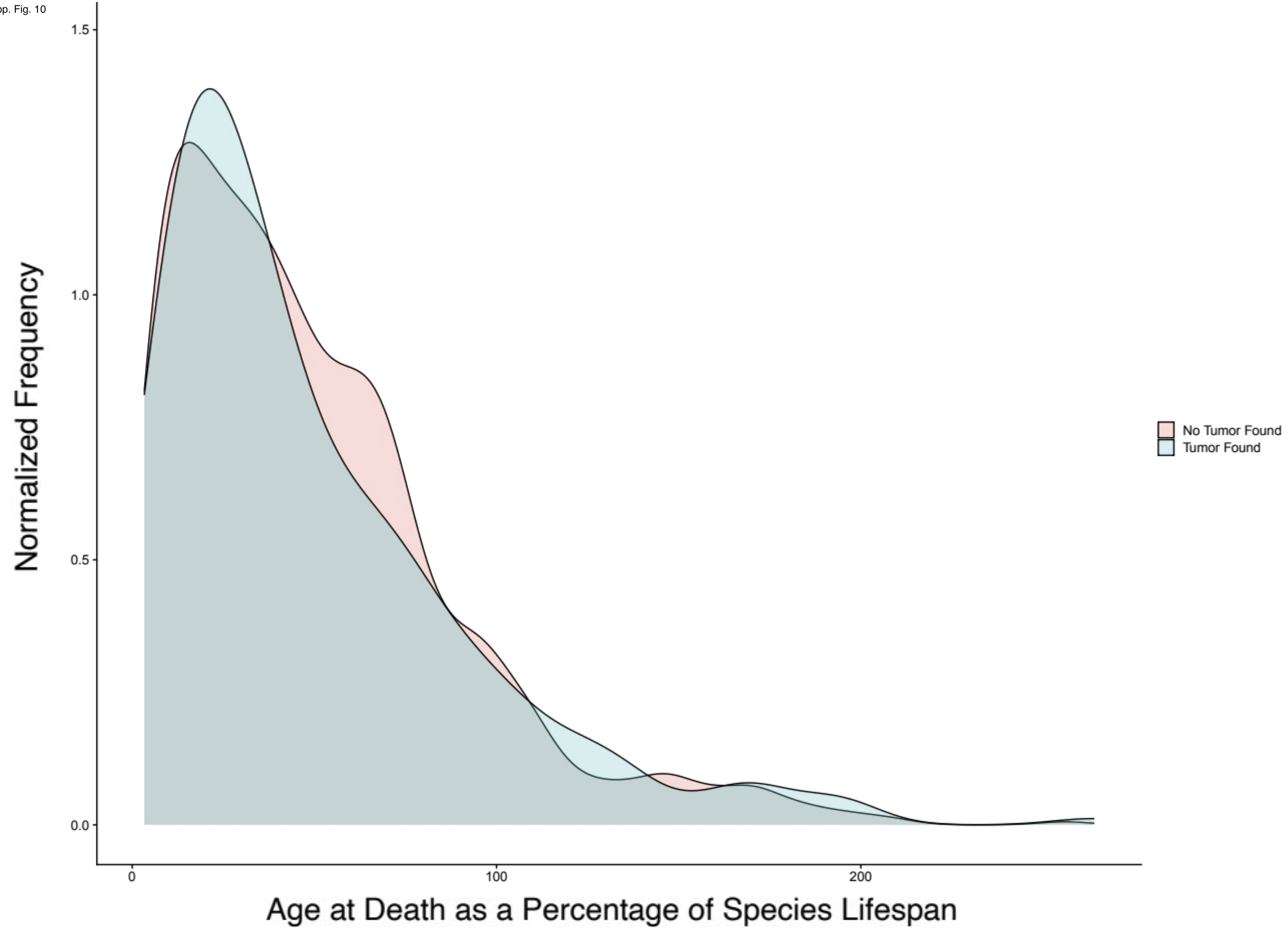

1.5
1.0
0.5
0.0
Normalized Frequency
0
100
200
Age at Death as a Percentage of Species Lifespan
No Tumor Found
Tumor Found

Supp. Fig. 11

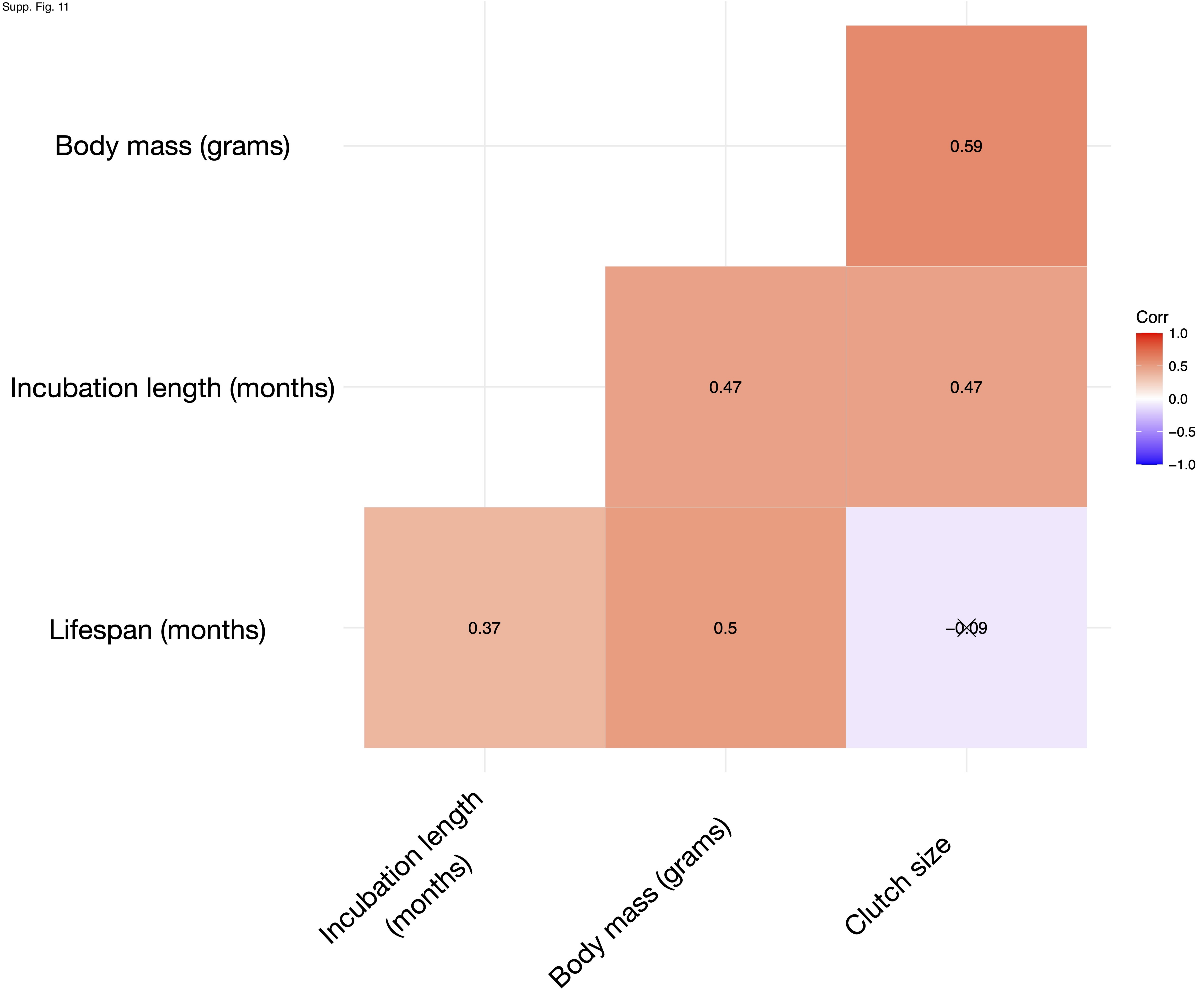
Body mass (grams)
Incubation length (months)
Lifespan (months)
0.59
0.47
0.47
0.37
0.5
−0.09
Corr
1.0
0.5
0.0
−0.5
−1.0
Incubation length (months)
Body mass (grams)
Clutch size

Supp. Fig. 12

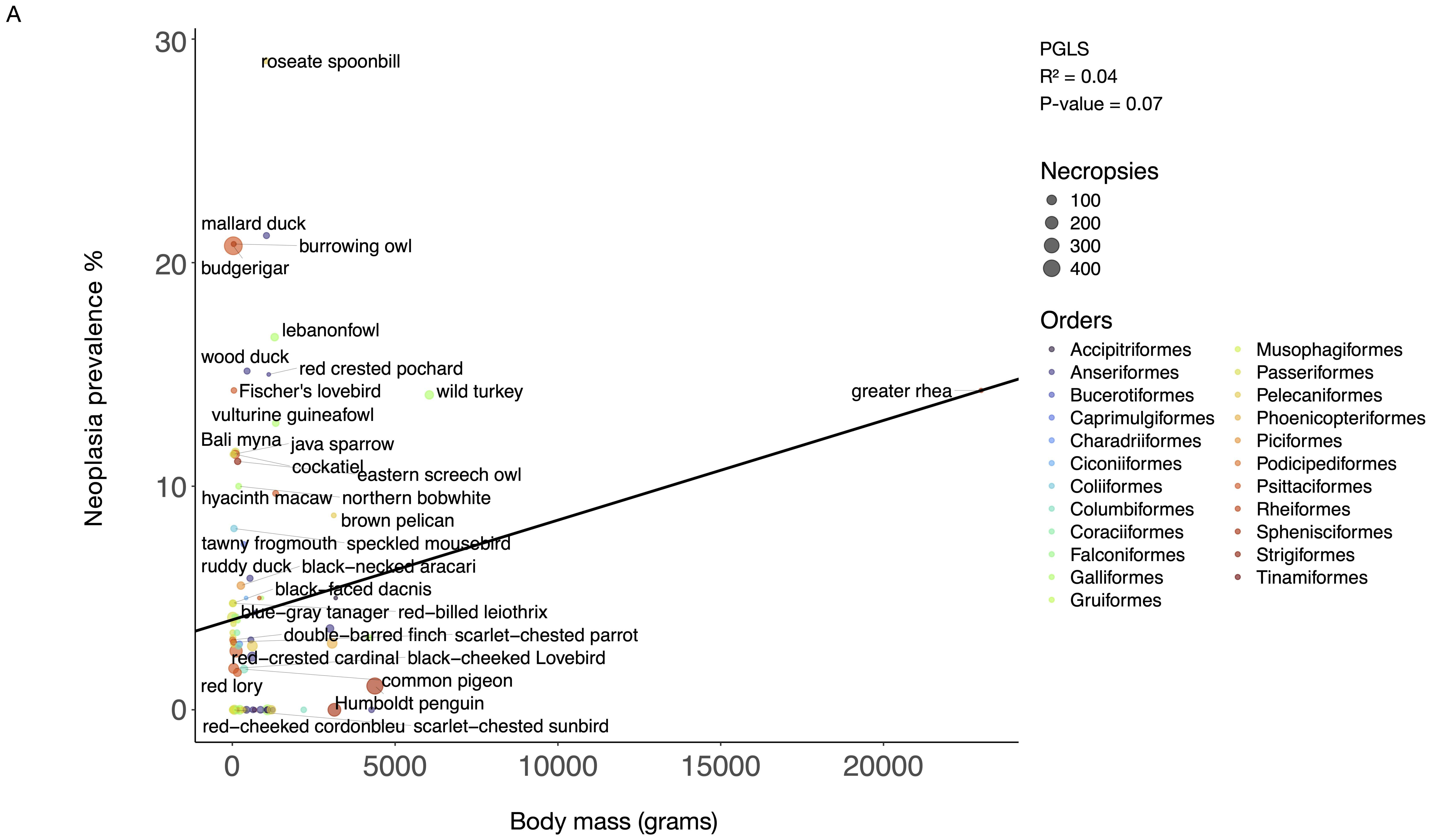
A
PGLS
R² = 0.04
P-value = 0.07
Necropsies
100
200
300
400
Orders
Accipitriformes
Anseriformes
Bucerotiformes
Caprimulgiformes
Charadriiformes
Ciconiiformes
Coliiformes
Columbiformes
Coraciiformes
Falconiformes
Galliformes
Gruiformes
Musophagiformes
Passeriformes
Pelecaniformes
Phoenicopteriformes
Piciformes
Podicipediformes
Psittaciformes
Rheiformes
Sphenisciformes
Strigiformes
Tinamiformes
Neoplasia prevalence %
Body mass (grams)
0
10
20
30
0
5000
10000
15000
20000
roseate spoonbill
mallard duck
burrowing owl
budgerigar
lebanonfowl
wood duck
red crested pochard
Fischer's lovebird
wild turkey
greater rhea
vulturine guineafowl
Bali myna
java sparrow
cockatiel
eastern screech owl
hyacinth macaw
northern bobwhite
brown pelican
tawny frogmouth
speckled mousebird
ruddy duck
black−necked aracari
black−faced dacnis
blue−gray tanager
red−billed leiothrix
double−barred finch
scarlet−chested parrot
red−crested cardinal
black−cheeked Lovebird
common pigeon
red lory
Humboldt penguin
red−cheeked cordonbleu
scarlet−chested sunbird

Supp. Fig. 12

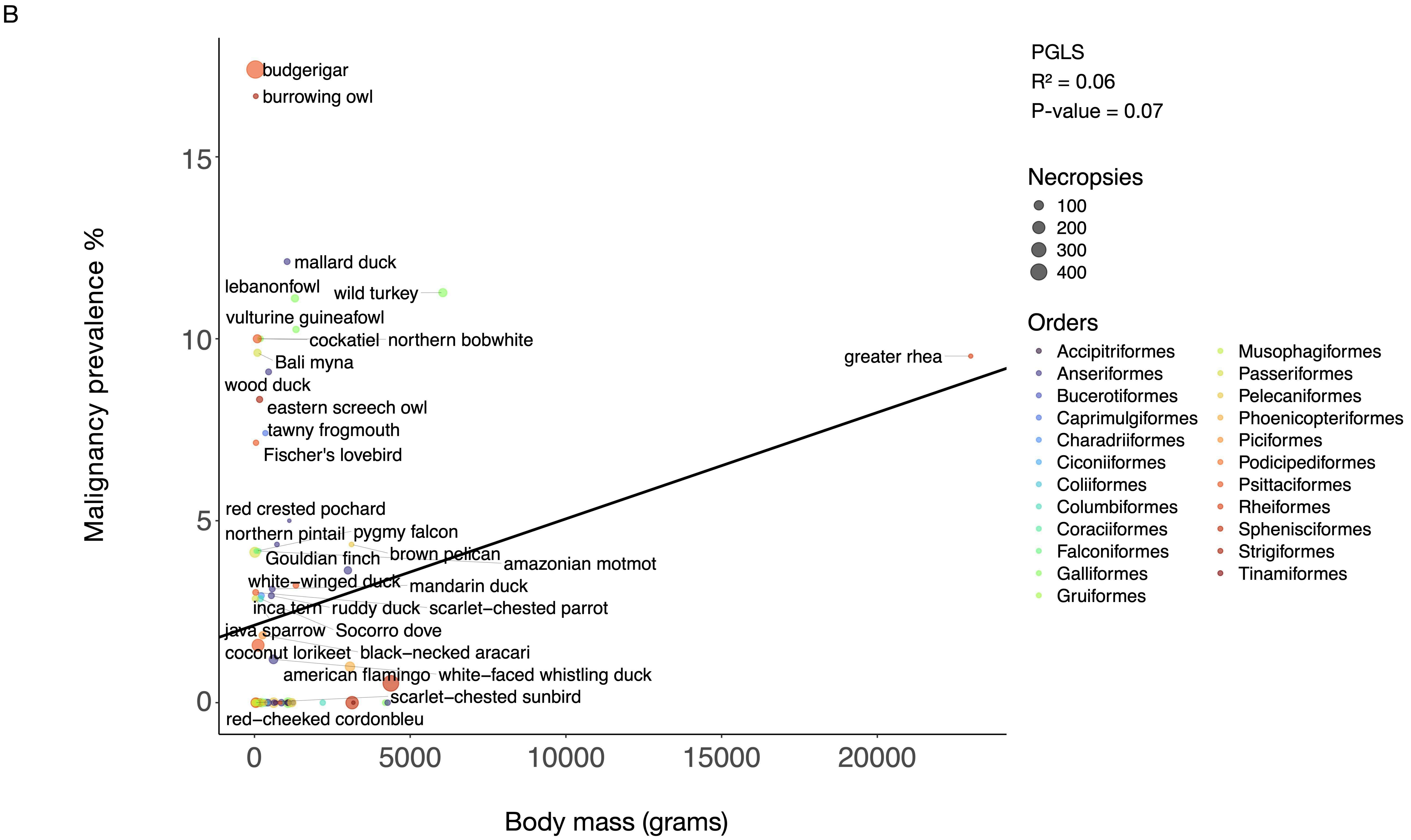

B
PGLS
R² = 0.06
P-value = 0.07
Necropsies
100
200
300
400
Orders
Accipitriformes
Anseriformes
Bucerotiformes
Caprimulgiformes
Charadriiformes
Ciconiiformes
Coliiformes
Columbiformes
Coraciiformes
Falconiformes
Galliformes
Gruiformes
Musophagiformes
Passeriformes
Pelecaniformes
Phoenicopteriformes
Piciformes
Podicipediformes
Psittaciformes
Rheiformes
Sphenisciformes
Strigiformes
Tinamiformes
Malignancy prevalence %
Body mass (grams)
0
5
10
15
0
5000
10000
15000
20000
budgerigar
burrowing owl
mallard duck
lebanonfowl
wild turkey
vulturine guineafowl
cockatiel
northern bobwhite
Bali myna
wood duck
eastern screech owl
tawny frogmouth
Fischer's lovebird
greater rhea
red crested pochard
northern pintail
pygmy falcon
Gouldian finch
brown pelican
amazonian motmot
white−winged duck
mandarin duck
inca tern
ruddy duck
scarlet−chested parrot
java sparrow
Socorro dove
coconut lorikeet
black−necked aracari
american flamingo
white−faced whistling duck
scarlet−chested sunbird
red−cheeked cordonbleu

Supp. Fig. 13

A

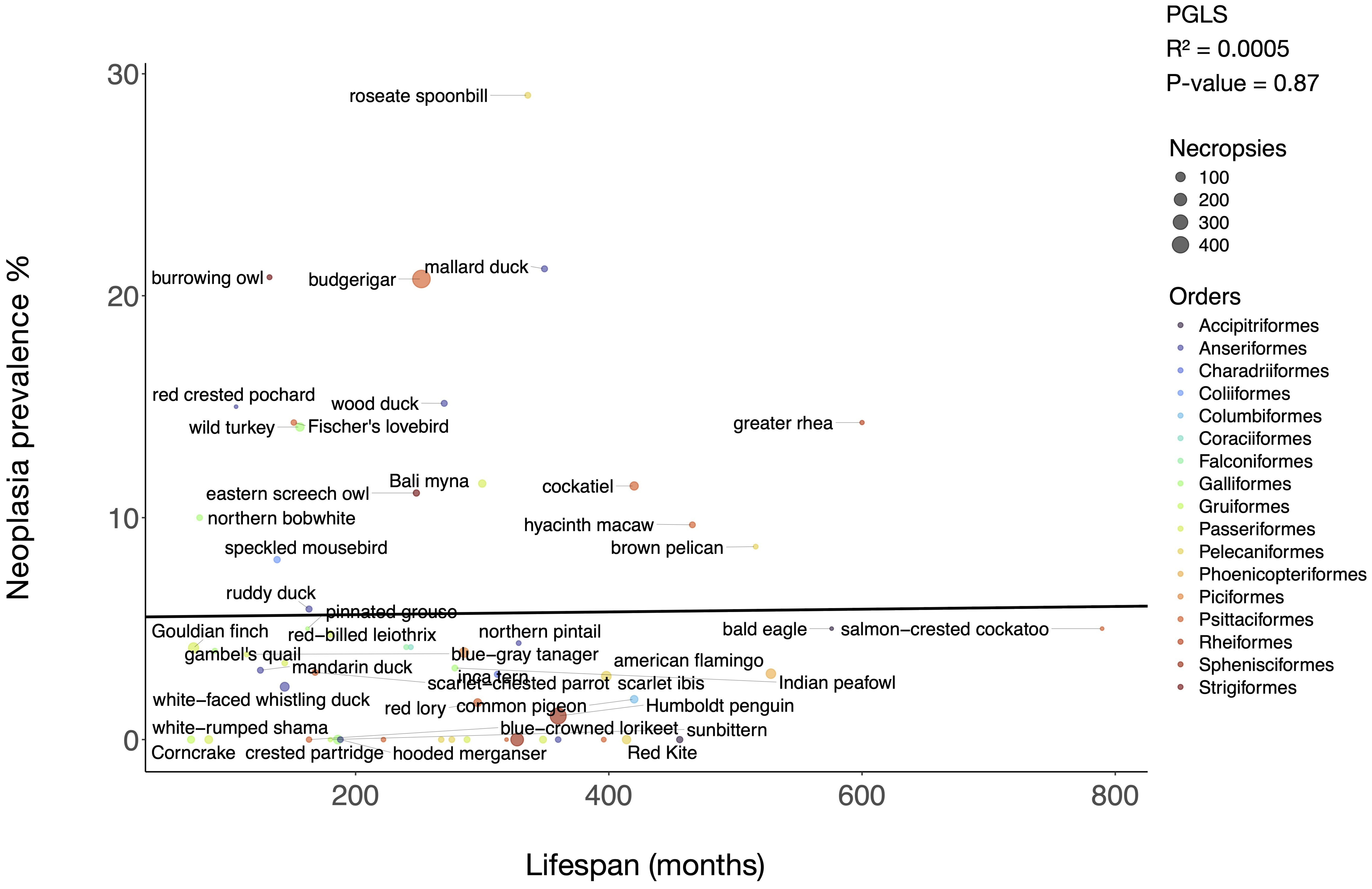

PGLS
R² = 0.0005
P-value = 0.87
Necropsies
100
200
300
400
Orders
Accipitriformes
Anseriformes
Charadriiformes
Coliiformes
Columbiformes
Coraciiformes
Falconiformes
Galliformes
Gruiformes
Passeriformes
Pelecaniformes
Phoenicopteriformes
Piciformes
Psittaciformes
Rheiformes
Sphenisciformes
Strigiformes
Neoplasia prevalence %
Lifespan (months)
0
10
20
30
200
400
600
800
roseate spoonbill
burrowing owl
budgerigar
mallard duck
red crested pochard
wood duck
wild turkey
Fischer's lovebird
greater rhea
eastern screech owl
Bali myna
cockatiel
northern bobwhite
hyacinth macaw
speckled mousebird
brown pelican
ruddy duck
pinnated grouse
Gouldian finch
red–billed leiothrix
northern pintail
bald eagle
salmon–crested cockatoo
gambel's quail
blue–gray tanager
american flamingo
mandarin duck
inca tern
scarlet–chested parrot
scarlet ibis
Indian peafowl
white–faced whistling duck
red lory
common pigeon
Humboldt penguin
white–rumped shama
blue–crowned lorikeet
sunbittern
Corncrake
crested partridge
hooded merganser
Red Kite

B

Supp. Fig. 13

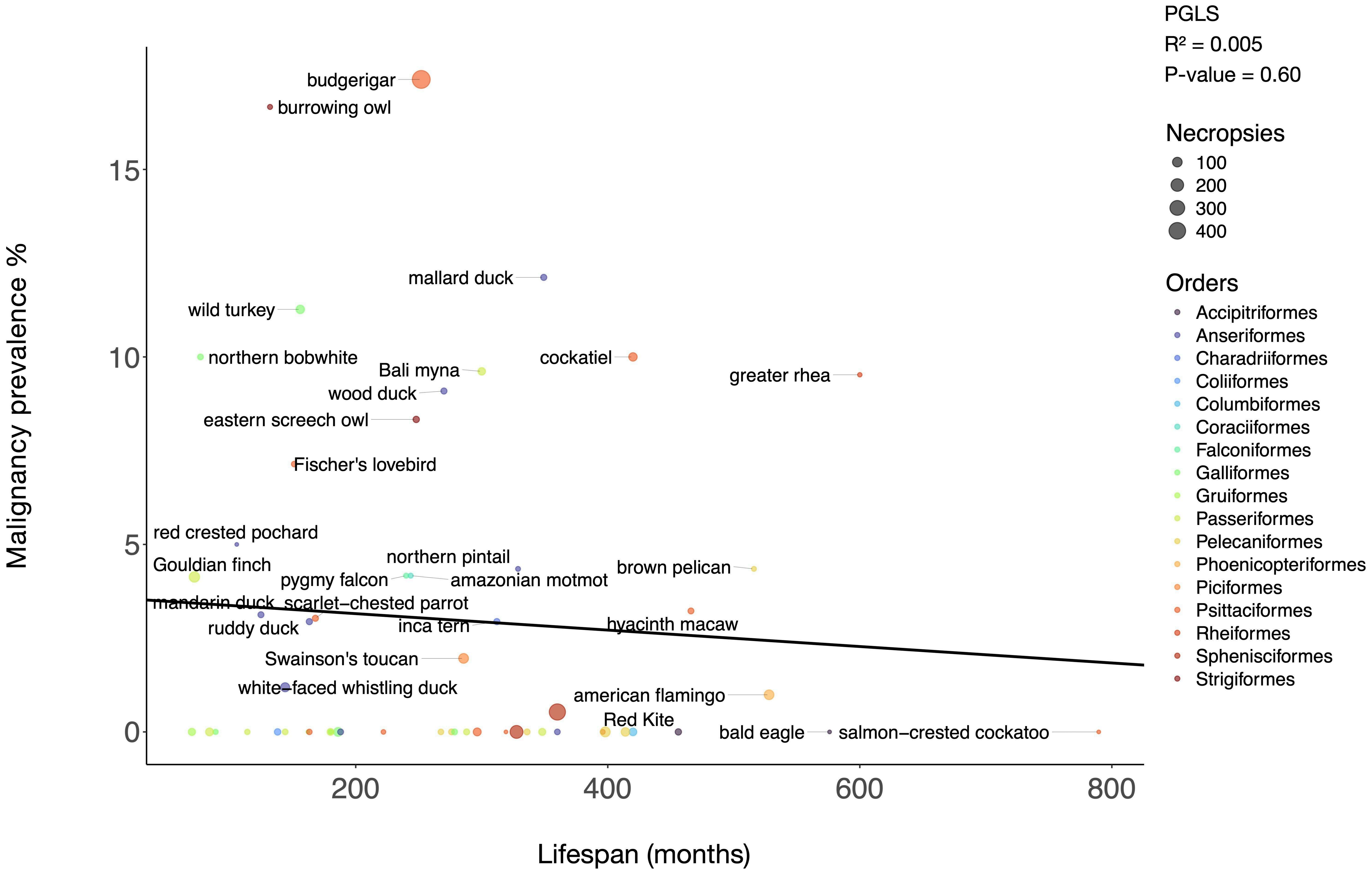

PGLS
R² = 0.005
P-value = 0.60
Necropsies
100
200
300
400
Orders
Accipitriformes
Anseriformes
Charadriiformes
Coliiformes
Columbiformes
Coraciiformes
Falconiformes
Galliformes
Gruiformes
Passeriformes
Pelecaniformes
Phoenicopteriformes
Piciformes
Psittaciformes
Rheiformes
Sphenisciformes
Strigiformes
Malignancy prevalence %
Lifespan (months)
0
5
10
15
200
400
600
800
budgerigar
burrowing owl
mallard duck
wild turkey
northern bobwhite
cockatiel
Bali myna
greater rhea
wood duck
eastern screech owl
Fischer's lovebird
red crested pochard
Gouldian finch
northern pintail
pygmy falcon
amazonian motmot
brown pelican
mandarin duck
scarlet−chested parrot
ruddy duck
inca tern
hyacinth macaw
Swainson's toucan
white−faced whistling duck
american flamingo
Red Kite
bald eagle
salmon−crested cockatoo

Supp. Fig. 14

A

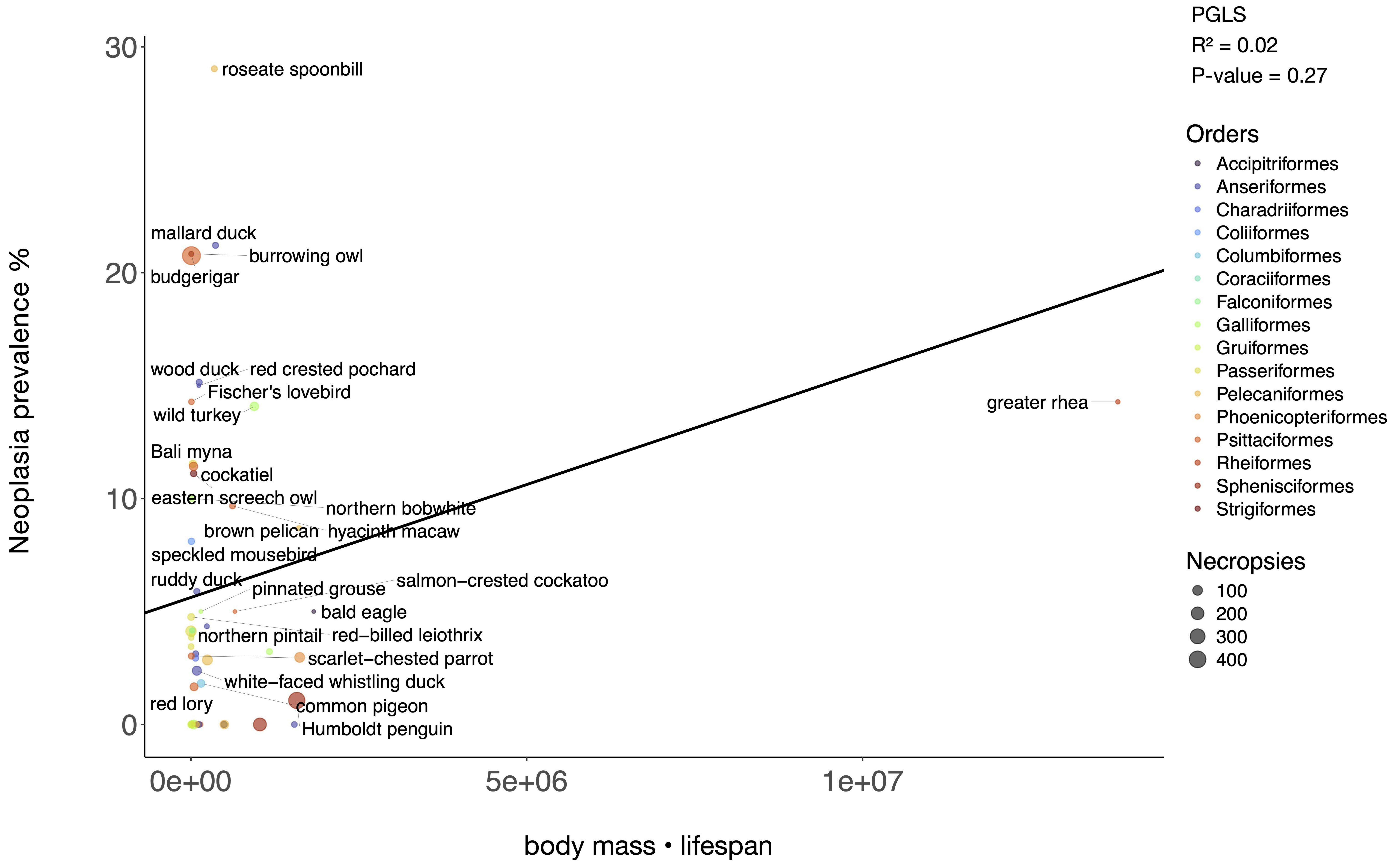

PGLS
R² = 0.02
P-value = 0.27
Orders
Accipitriformes
Anseriformes
Charadriiformes
Coliiformes
Columbiformes
Coraciiformes
Falconiformes
Galliformes
Gruiformes
Passeriformes
Pelecaniformes
Phoenicopteriformes
Psittaciformes
Rheiformes
Sphenisciformes
Strigiformes
Necropsies
100
200
300
400
Neoplasia prevalence %
body mass • lifespan
0e+00
5e+06
1e+07
0
10
20
30
roseate spoonbill
mallard duck
burrowing owl
budgerigar
wood duck
red crested pochard
Fischer's lovebird
wild turkey
greater rhea
Bali myna
cockatiel
eastern screech owl
northern bobwhite
brown pelican
hyacinth macaw
speckled mousebird
ruddy duck
pinnated grouse
salmon–crested cockatoo
bald eagle
northern pintail
red–billed leiothrix
scarlet–chested parrot
white–faced whistling duck
red lory
common pigeon
Humboldt penguin

Supp. Fig. 14

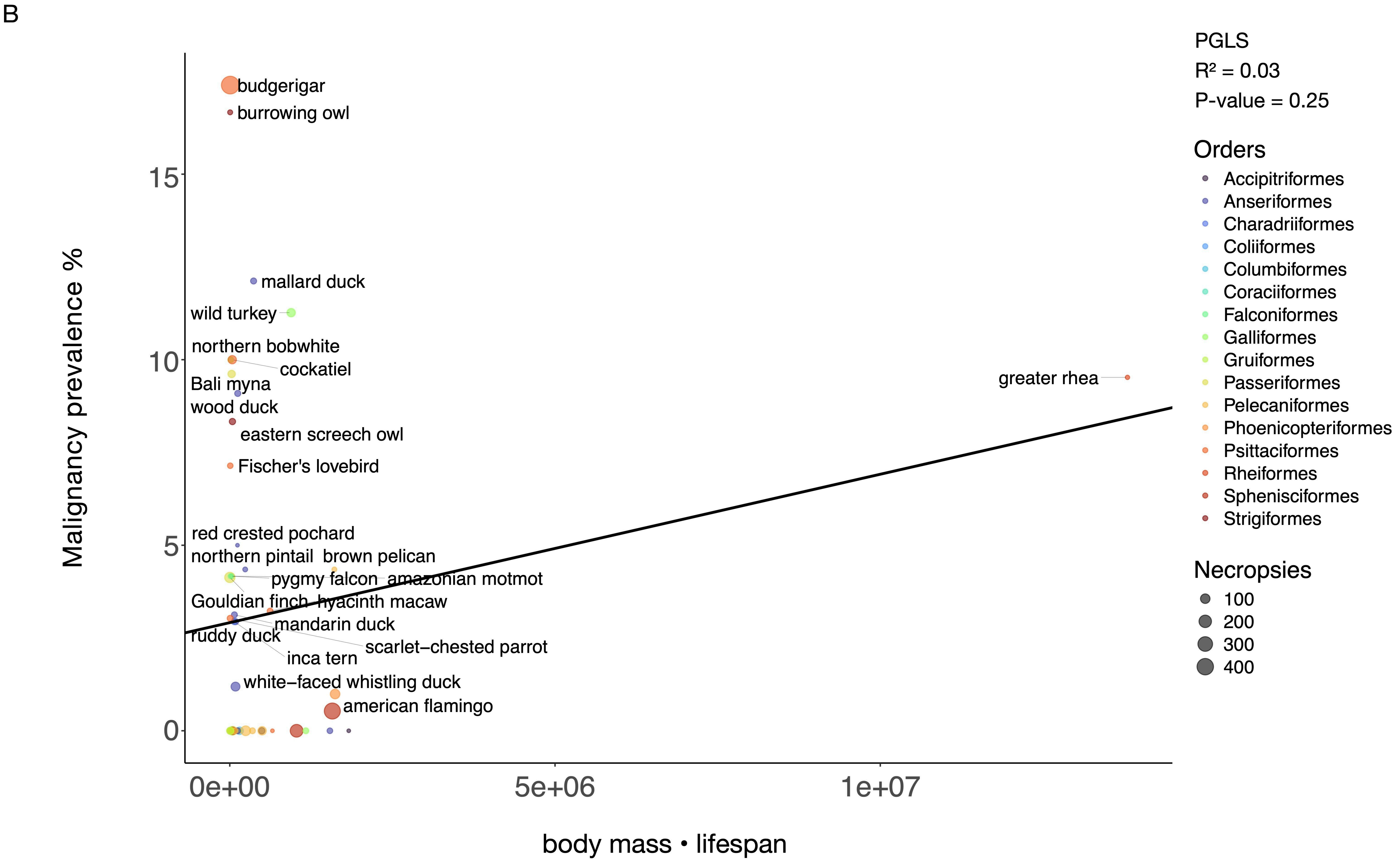
B
PGLS
R² = 0.03
P-value = 0.25
Orders
Accipitriformes
Anseriformes
Charadriiformes
Coliiformes
Columbiformes
Coraciiformes
Falconiformes
Galliformes
Gruiformes
Passeriformes
Pelecaniformes
Phoenicopteriformes
Psittaciformes
Rheiformes
Sphenisciformes
Strigiformes
Necropsies
100
200
300
400
Malignancy prevalence %
body mass • lifespan
0e+00
5e+06
1e+07
0
5
10
15
budgerigar
burrowing owl
mallard duck
wild turkey
northern bobwhite
cockatiel
Bali myna
wood duck
eastern screech owl
Fischer's lovebird
greater rhea
red crested pochard
northern pintail
brown pelican
pygmy falcon
amazonian motmot
Gouldian finch
hyacinth macaw
mandarin duck
ruddy duck
scarlet−chested parrot
inca tern
white−faced whistling duck
american flamingo

Supp. Fig. 15

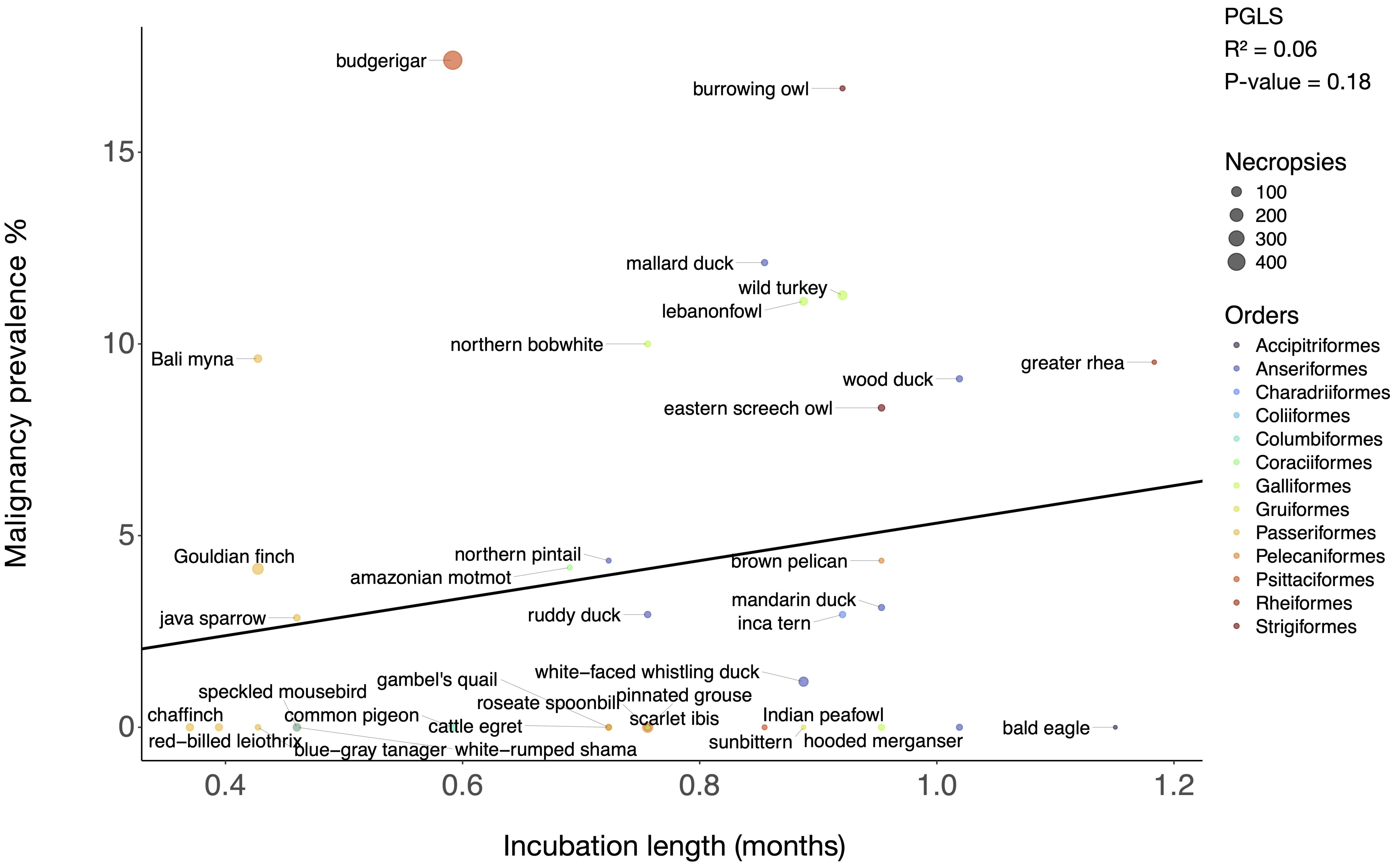

PGLS
R² = 0.06
P-value = 0.18
Necropsies
100
200
300
400
Orders
Accipitriformes
Anseriformes
Charadriiformes
Coliiformes
Columbiformes
Coraciiformes
Galliformes
Gruiformes
Passeriformes
Pelecaniformes
Psittaciformes
Rheiformes
Strigiformes
Malignancy prevalence %
Incubation length (months)
0
5
10
15
0.4
0.6
0.8
1.0
1.2
budgerigar
burrowing owl
mallard duck
wild turkey
lebanonfowl
northern bobwhite
Bali myna
wood duck
greater rhea
eastern screech owl
Gouldian finch
northern pintail
amazonian motmot
brown pelican
mandarin duck
java sparrow
ruddy duck
inca tern
white−faced whistling duck
gambel's quail
speckled mousebird
pinnated grouse
roseate spoonbill
scarlet ibis
chaffinch
common pigeon
Indian peafowl
cattle egret
bald eagle
red−billed leiothrix
sunbittern
hooded merganser
blue−gray tanager
white−rumped shama

Supp. Fig. 16

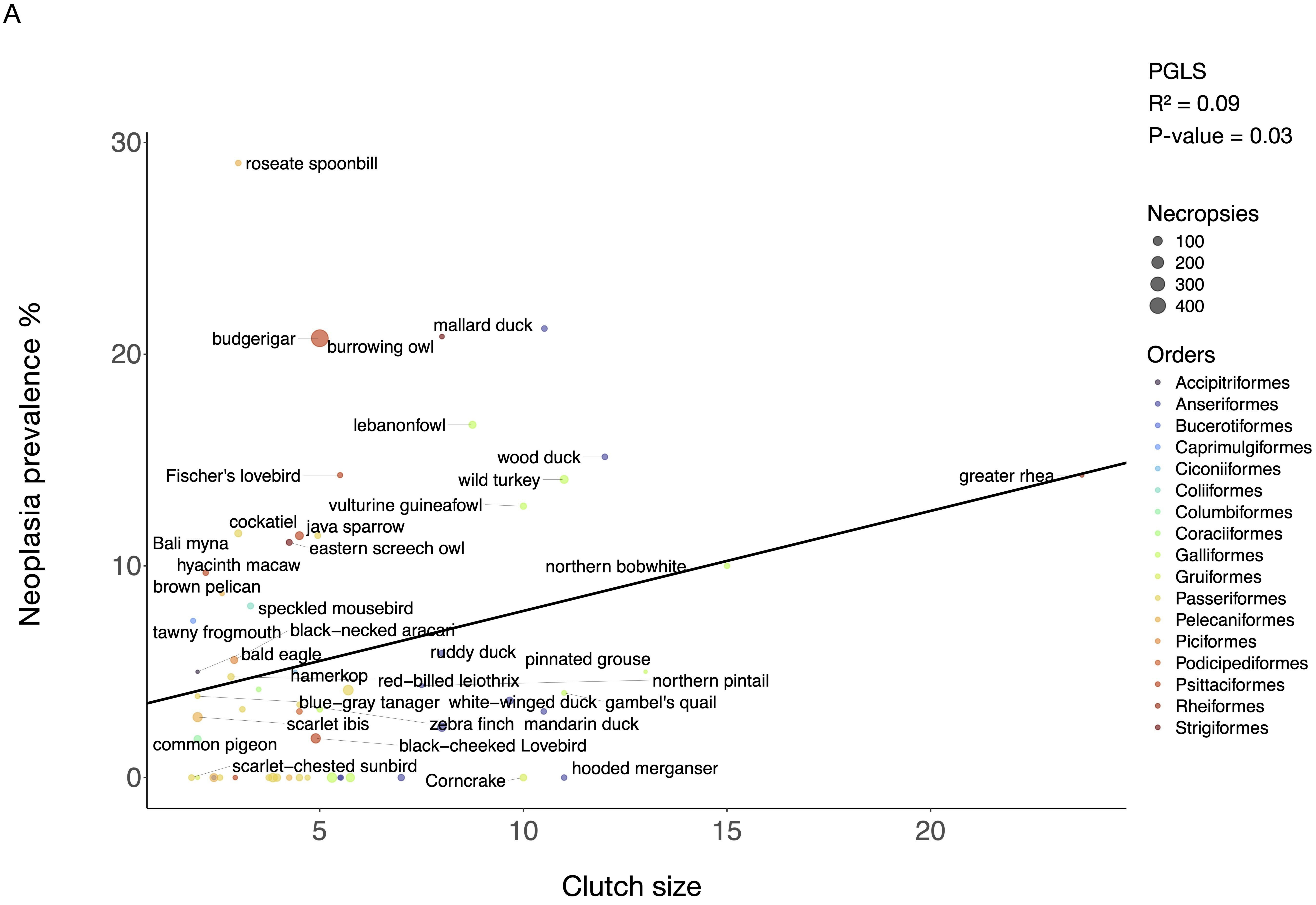
A
PGLS
R² = 0.09
P-value = 0.03
Necropsies
100
200
300
400
Orders
Accipitriformes
Anseriformes
Bucerotiformes
Caprimulgiformes
Ciconiiformes
Coliiformes
Columbiformes
Coraciiformes
Galliformes
Gruiformes
Passeriformes
Pelecaniformes
Piciformes
Podicipediformes
Psittaciformes
Rheiformes
Strigiformes
Neoplasia prevalence %
Clutch size
0
10
20
30
5
10
15
20
roseate spoonbill
budgerigar
burrowing owl
mallard duck
lebanonfowl
wood duck
Fischer's lovebird
wild turkey
greater rhea
vulturine guineafowl
cockatiel
java sparrow
Bali myna
eastern screech owl
hyacinth macaw
northern bobwhite
brown pelican
speckled mousebird
tawny frogmouth
black−necked aracari
bald eagle
ruddy duck
pinnated grouse
hamerkop
red−billed leiothrix
northern pintail
blue−gray tanager
white−winged duck
gambel's quail
scarlet ibis
zebra finch
mandarin duck
common pigeon
black−cheeked Lovebird
scarlet−chested sunbird
hooded merganser
Corncrake

B

Supp. Fig. 16

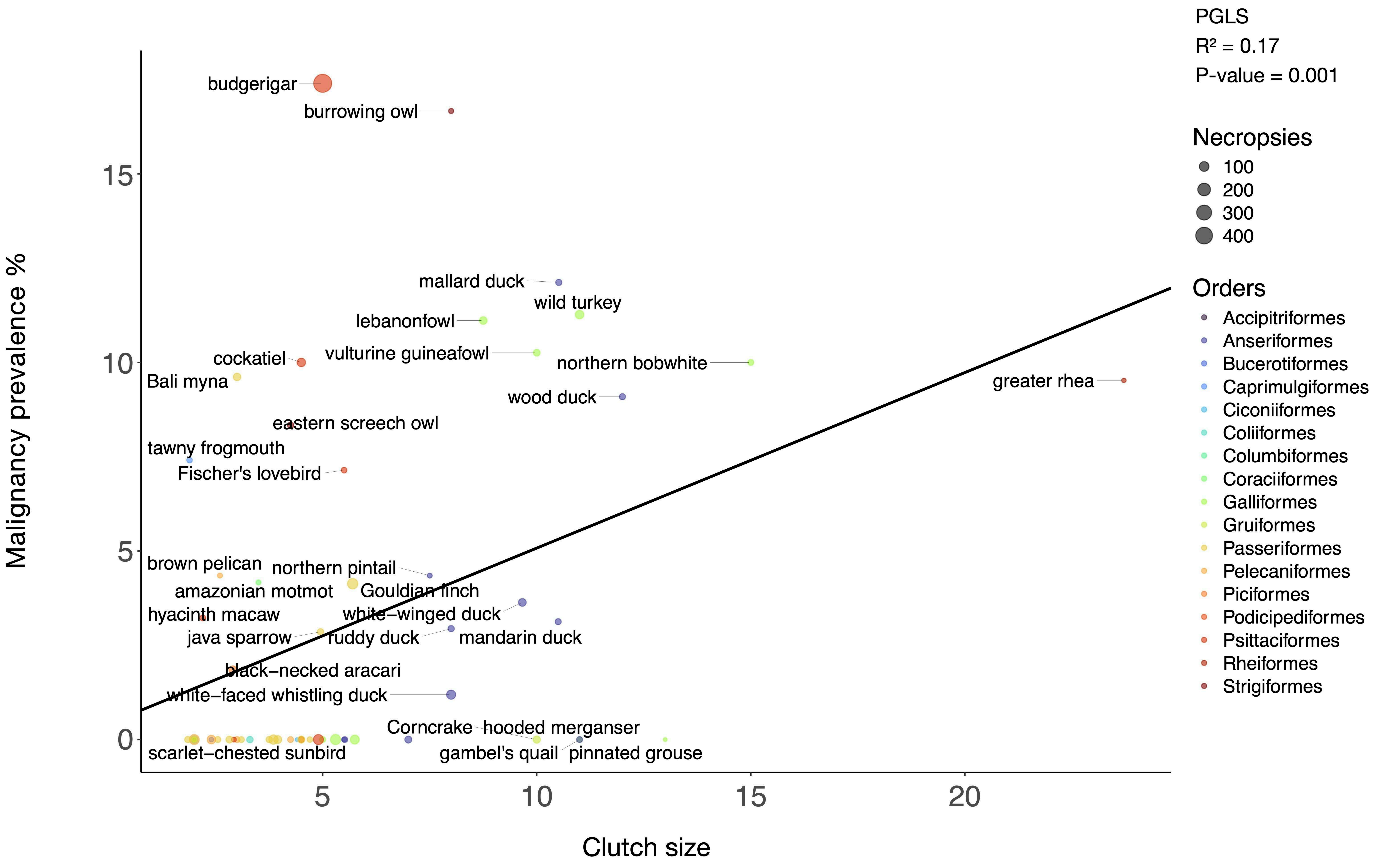

PGLS
R² = 0.17
P-value = 0.001
Necropsies
100
200
300
400
Orders
Accipitriformes
Anseriformes
Bucerotiformes
Caprimulgiformes
Ciconiiformes
Coliiformes
Columbiformes
Coraciiformes
Galliformes
Gruiformes
Passeriformes
Pelecaniformes
Piciformes
Podicipediformes
Psittaciformes
Rheiformes
Strigiformes
Malignancy prevalence %
Clutch size
0
5
10
15
20
budgerigar
burrowing owl
mallard duck
wild turkey
lebanonfowl
vulturine guineafowl
northern bobwhite
cockatiel
Bali myna
greater rhea
wood duck
eastern screech owl
tawny frogmouth
Fischer's lovebird
brown pelican
northern pintail
amazonian motmot
Gouldian finch
hyacinth macaw
white−winged duck
java sparrow
ruddy duck
mandarin duck
black−necked aracari
white−faced whistling duck
Corncrake
hooded merganser
scarlet−chested sunbird
gambel's quail
pinnated grouse

A

Supp. Fig. 17

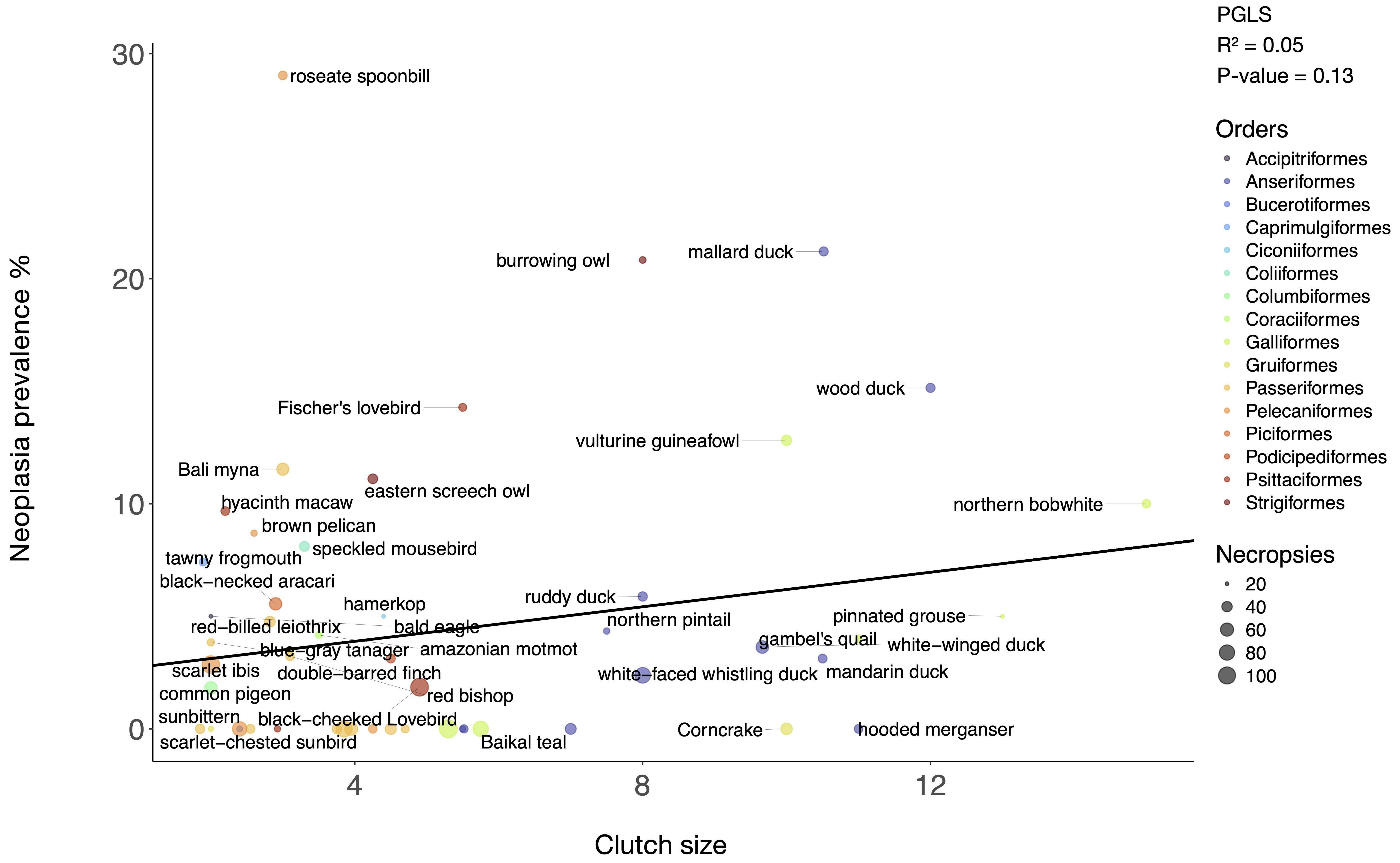

PGLS
R² = 0.05
P-value = 0.13
Orders
Accipitriformes
Anseriformes
Bucerotiformes
Caprimulgiformes
Ciconiiformes
Coliiformes
Columbiformes
Coraciiformes
Galliformes
Gruiformes
Passeriformes
Pelecaniformes
Piciformes
Podicipediformes
Psittaciformes
Strigiformes
Necropsies
20
40
60
80
100
Neoplasia prevalence %
Clutch size
30
20
10
0
4
8
12
roseate spoonbill
burrowing owl
mallard duck
wood duck
Fischer's lovebird
vulturine guineafowl
Bali myna
eastern screech owl
hyacinth macaw
northern bobwhite
brown pelican
speckled mousebird
tawny frogmouth
black–necked aracari
ruddy duck
hamerkop
pinnated grouse
red–billed leiothrix
bald eagle
northern pintail
gambel's quail
white–winged duck
blue–gray tanager
amazonian motmot
scarlet ibis
double–barred finch
white–faced whistling duck
mandarin duck
common pigeon
red bishop
sunbittern
black–cheeked Lovebird
Corncrake
hooded merganser
scarlet–chested sunbird
Baikal teal

B

Supp. Fig. 17

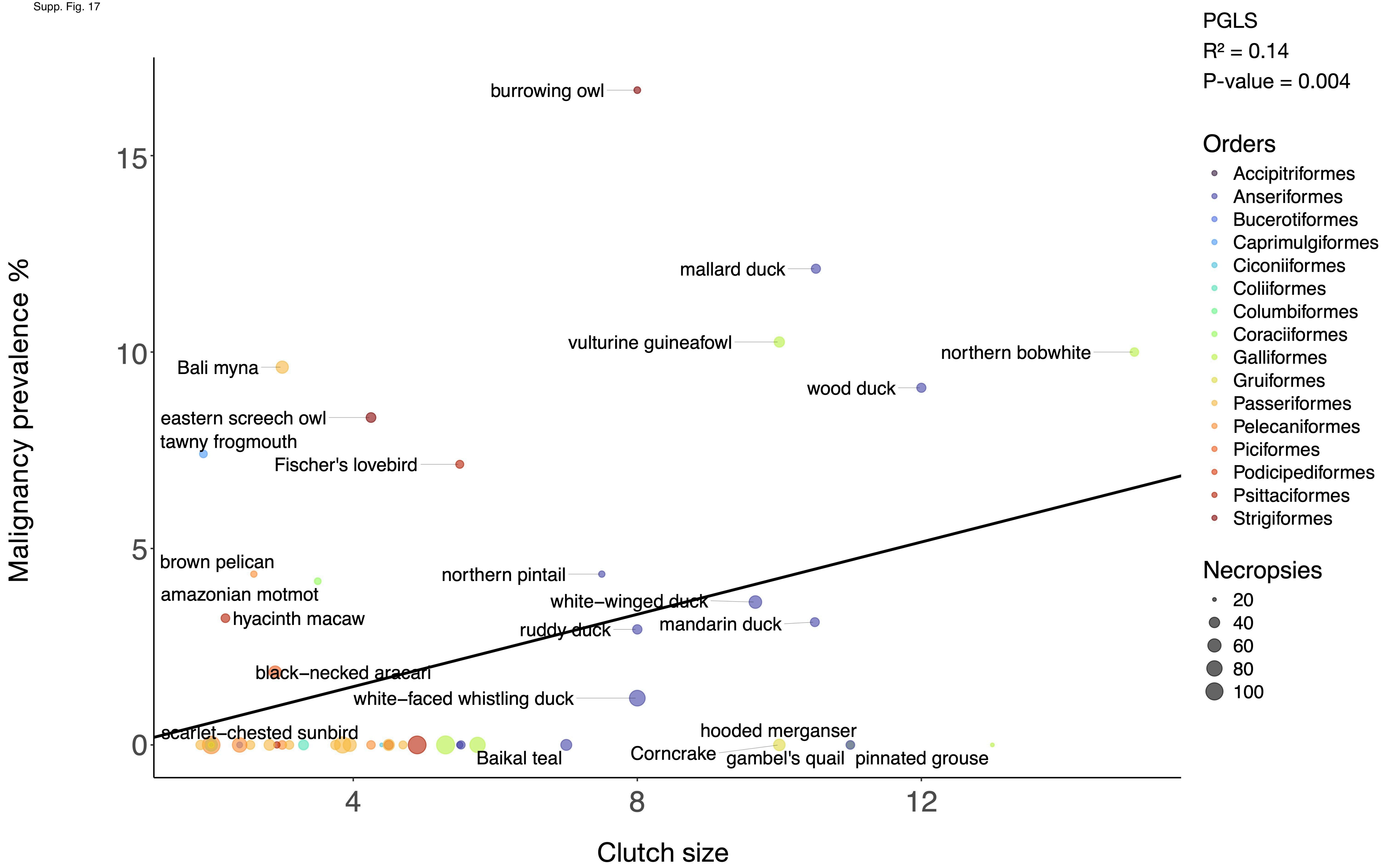
PGLS
R² = 0.14
P-value = 0.004
Orders
Accipitriformes
Anseriformes
Bucerotiformes
Caprimulgiformes
Ciconiiformes
Coliiformes
Columbiformes
Coraciiformes
Galliformes
Gruiformes
Passeriformes
Pelecaniformes
Piciformes
Podicipediformes
Psittaciformes
Strigiformes
Necropsies
20
40
60
80
100
Malignancy prevalence %
Clutch size
0
5
10
15
4
8
12
burrowing owl
mallard duck
vulturine guineafowl
northern bobwhite
Bali myna
wood duck
eastern screech owl
tawny frogmouth
Fischer's lovebird
brown pelican
northern pintail
amazonian motmot
white–winged duck
hyacinth macaw
mandarin duck
ruddy duck
black–necked aracari
white–faced whistling duck
hooded merganser
scarlet–chested sunbird
Corncrake
Baikal teal
gambel's quail
pinnated grouse

Supp. Fig. 18

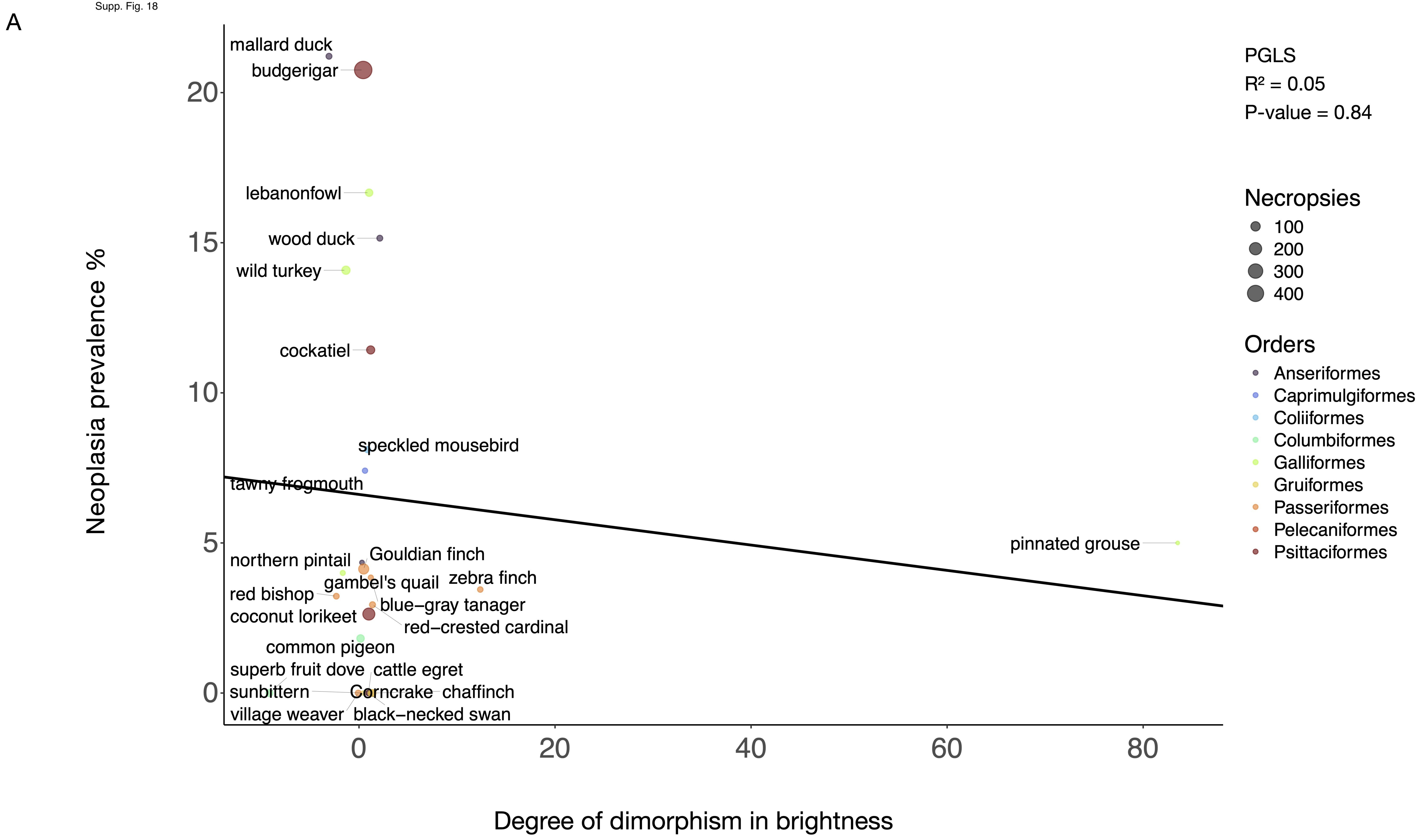
A
mallard duck
budgerigar
lebanonfowl
wood duck
wild turkey
cockatiel
speckled mousebird
tawny frogmouth
pinnated grouse
northern pintail
Gouldian finch
gambel's quail
zebra finch
red bishop
blue–gray tanager
coconut lorikeet
red–crested cardinal
common pigeon
superb fruit dove
cattle egret
sunbittern
Corncrake
chaffinch
village weaver
black–necked swan
Neoplasia prevalence %
0
5
10
15
20
0
20
40
60
80
Degree of dimorphism in brightness
PGLS
R² = 0.05
P-value = 0.84
Necropsies
100
200
300
400
Orders
Anseriformes
Caprimulgiformes
Coliiformes
Columbiformes
Galliformes
Gruiformes
Passeriformes
Pelecaniformes
Psittaciformes

B

Supp. Fig. 18

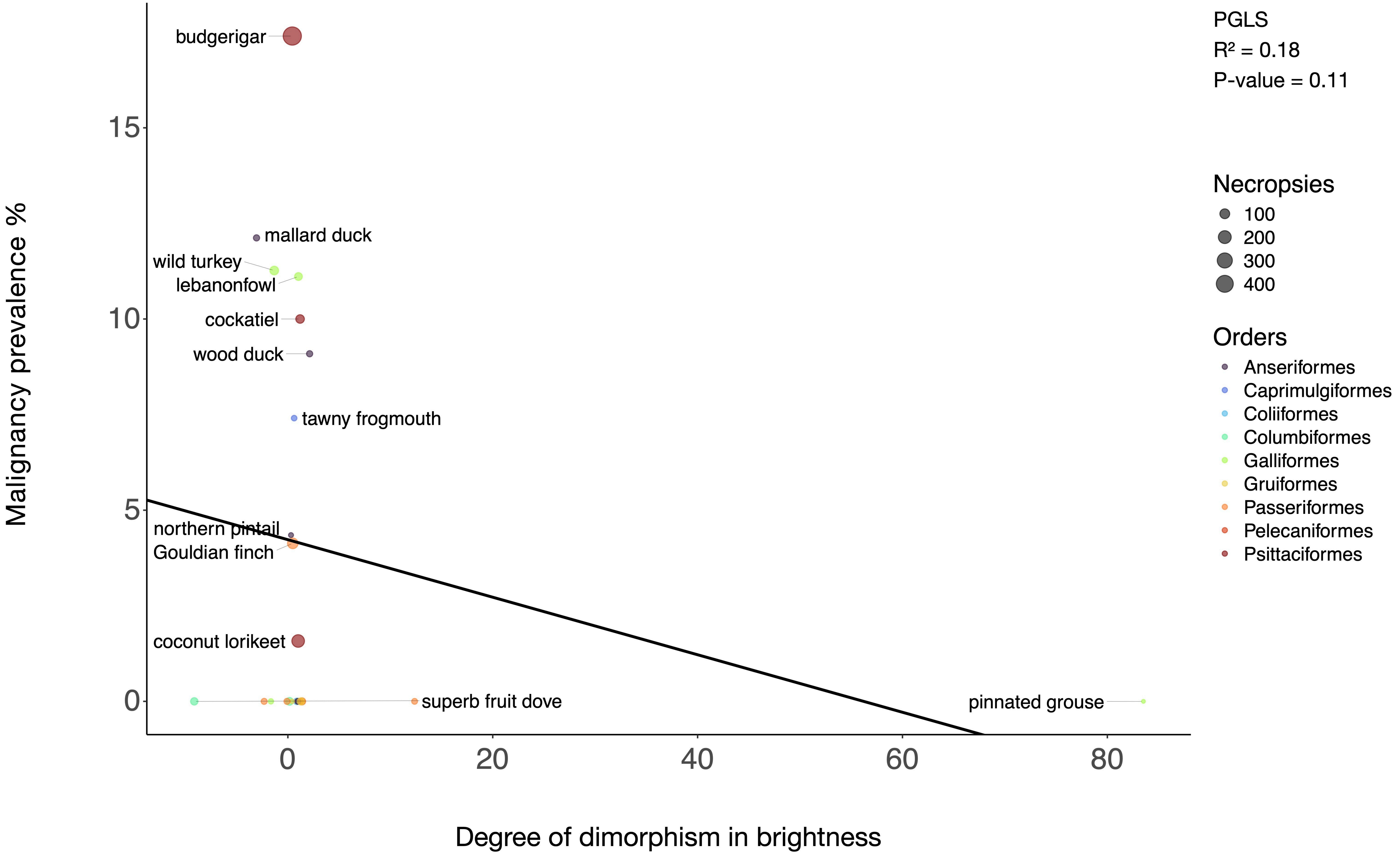

PGLS
R² = 0.18
P-value = 0.11
Necropsies
100
200
300
400
Orders
Anseriformes
Caprimulgiformes
Coliiformes
Columbiformes
Galliformes
Gruiformes
Passeriformes
Pelecaniformes
Psittaciformes
budgerigar
mallard duck
wild turkey
lebanonfowl
cockatiel
wood duck
tawny frogmouth
northern pintail
Gouldian finch
coconut lorikeet
superb fruit dove
pinnated grouse
Malignancy prevalence %
0
5
10
15
Degree of dimorphism in brightness
0
20
40
60
80

Supp. Fig. 18

C

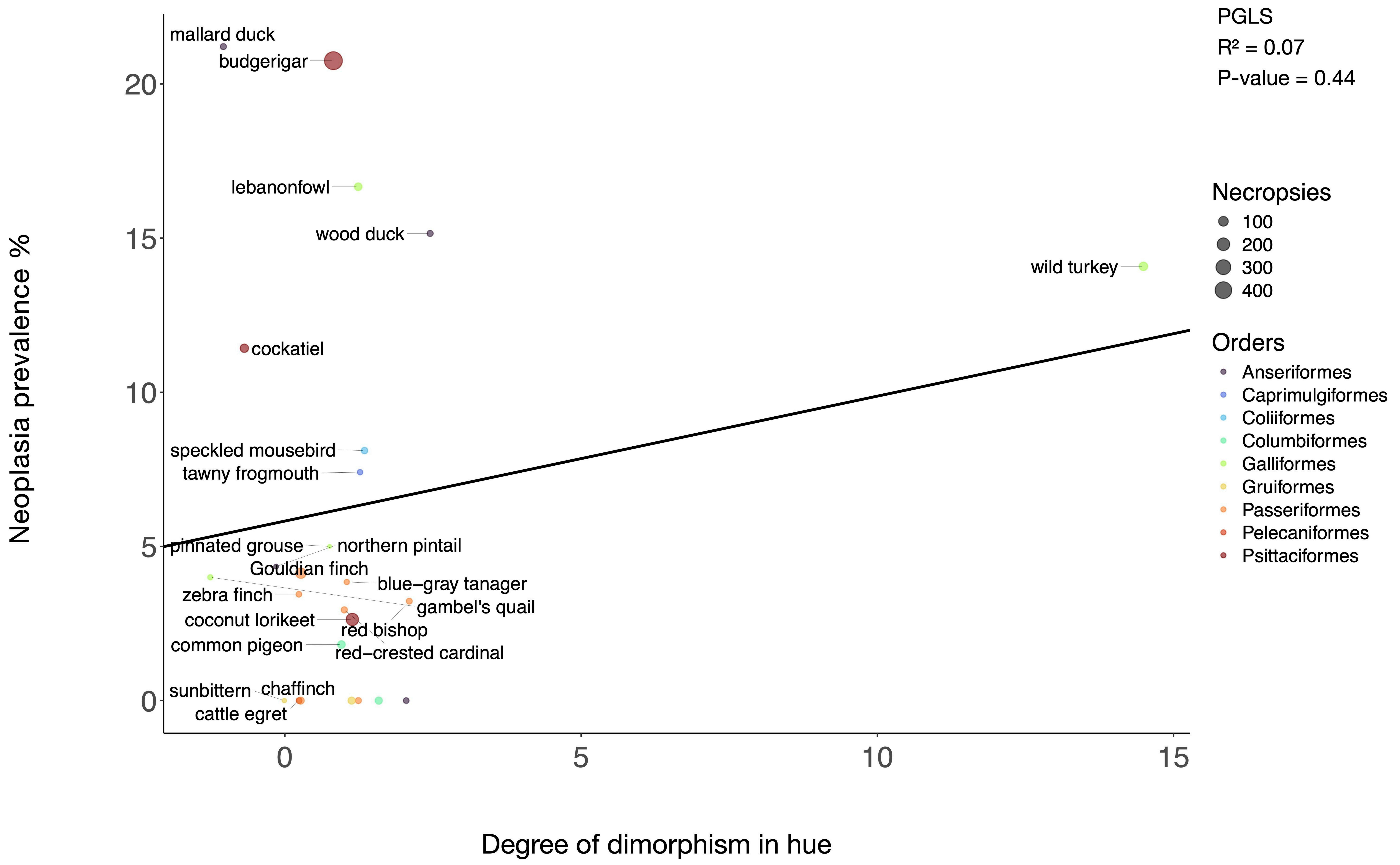

PGLS
R² = 0.07
P-value = 0.44
Neoplasia prevalence %
Degree of dimorphism in hue
0
5
10
15
20
mallard duck
budgerigar
lebanonfowl
wood duck
wild turkey
cockatiel
speckled mousebird
tawny frogmouth
pinnated grouse
northern pintail
Gouldian finch
blue−gray tanager
zebra finch
gambel's quail
coconut lorikeet
red bishop
common pigeon
red−crested cardinal
sunbittern
chaffinch
cattle egret
Necropsies
100
200
300
400
Orders
Anseriformes
Caprimulgiformes
Coliiformes
Columbiformes
Galliformes
Gruiformes
Passeriformes
Pelecaniformes
Psittaciformes

Supp. Fig. 18

D

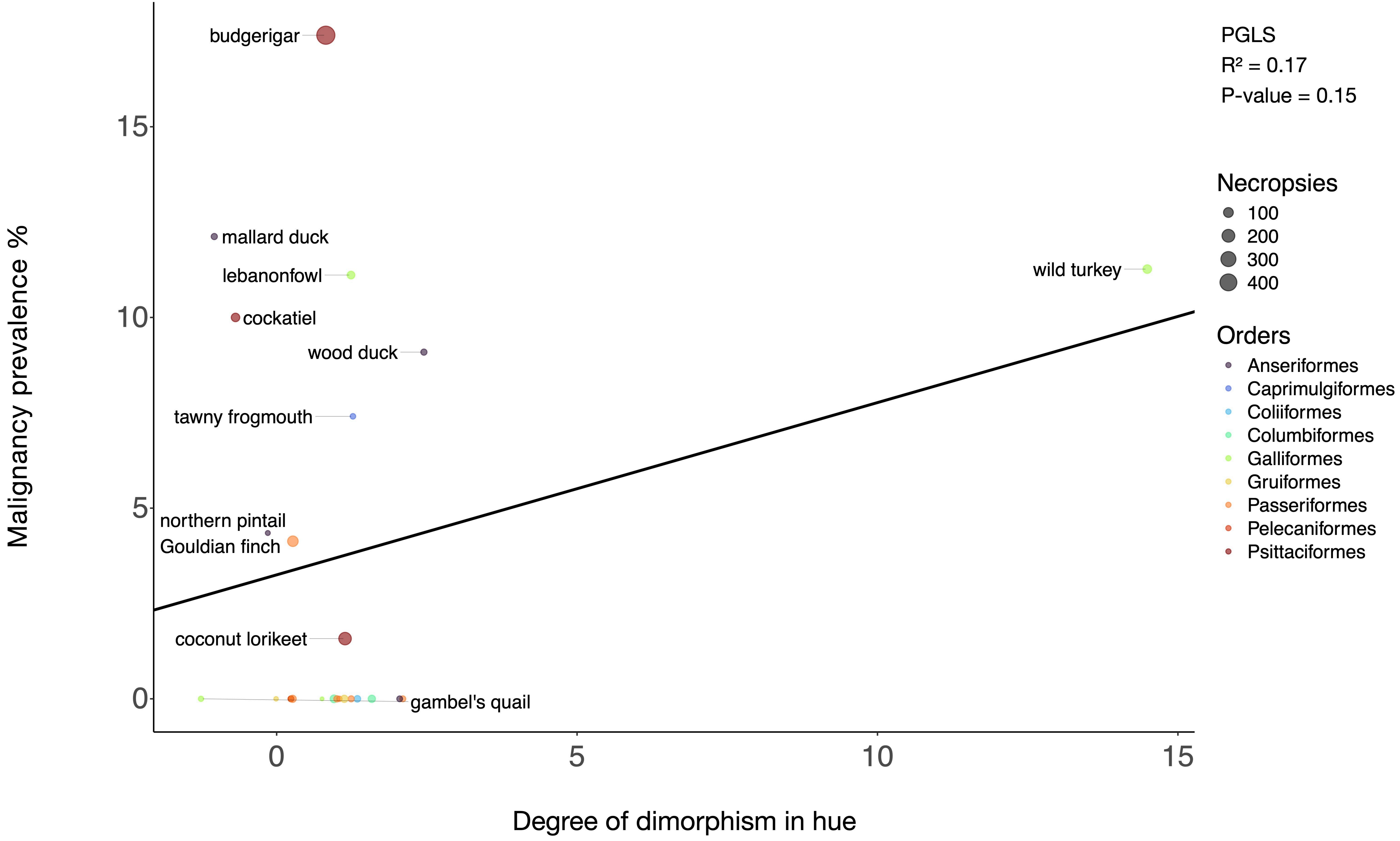

budgerigar
mallard duck
lebanonfowl
cockatiel
wood duck
tawny frogmouth
northern pintail
Gouldian finch
coconut lorikeet
gambel's quail
wild turkey
PGLS
R² = 0.17
P-value = 0.15
Necropsies
100
200
300
400
Orders
Anseriformes
Caprimulgiformes
Coliiformes
Columbiformes
Galliformes
Gruiformes
Passeriformes
Pelecaniformes
Psittaciformes
Malignancy prevalence %
Degree of dimorphism in hue
0
5
10
15

E

Supp. Fig. 18

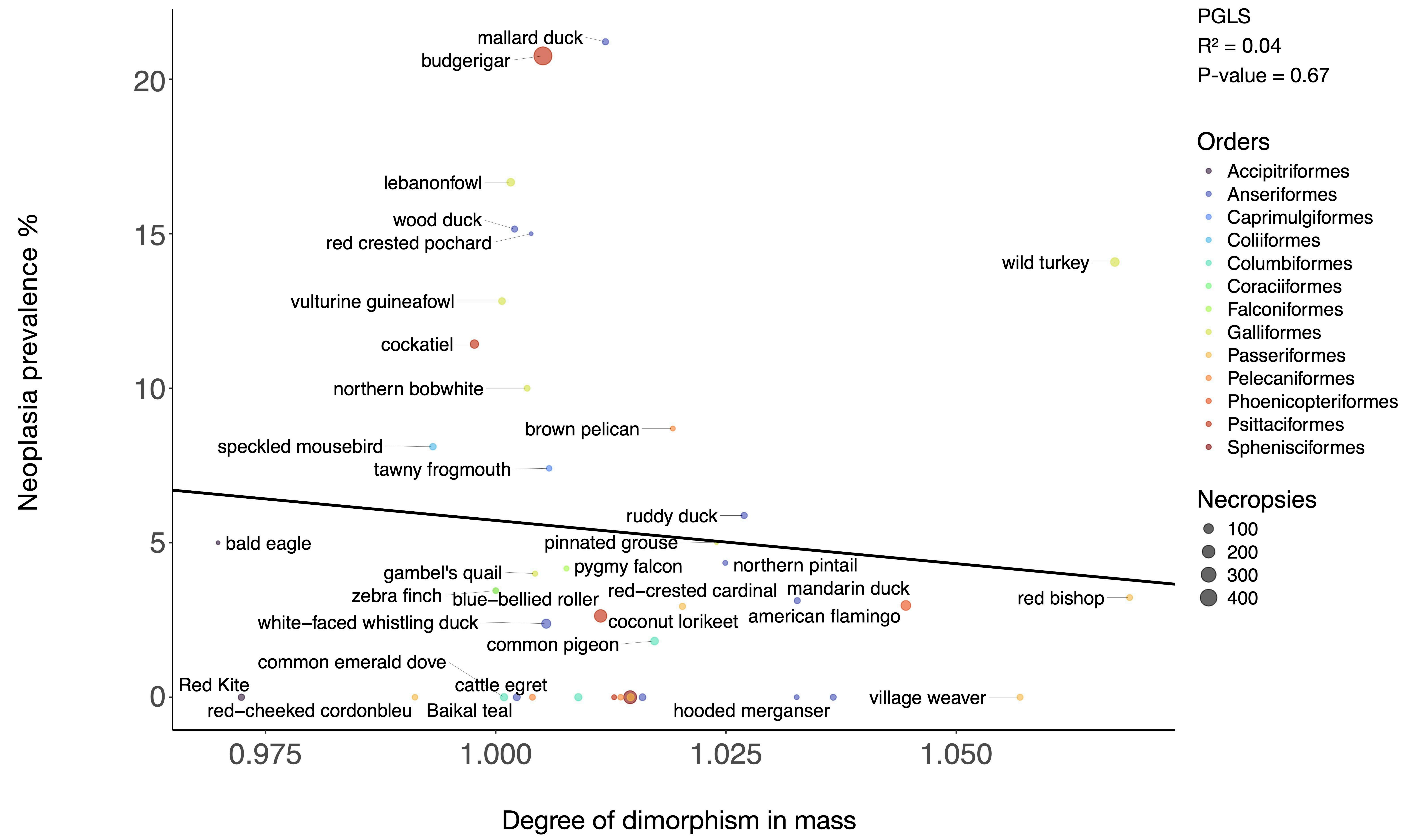

PGLS
R² = 0.04
P-value = 0.67
Orders
Accipitriformes
Anseriformes
Caprimulgiformes
Coliiformes
Columbiformes
Coraciiformes
Falconiformes
Galliformes
Passeriformes
Pelecaniformes
Phoenicopteriformes
Psittaciformes
Sphenisciformes
Necropsies
100
200
300
400
Neoplasia prevalence %
Degree of dimorphism in mass
0
5
10
15
20
0.975
1.000
1.025
1.050
mallard duck
budgerigar
lebanonfowl
wood duck
red crested pochard
wild turkey
vulturine guineafowl
cockatiel
northern bobwhite
brown pelican
speckled mousebird
tawny frogmouth
ruddy duck
bald eagle
pinnated grouse
gambel's quail
pygmy falcon
northern pintail
zebra finch
blue−bellied roller
red−crested cardinal
mandarin duck
red bishop
white−faced whistling duck
coconut lorikeet
american flamingo
common pigeon
common emerald dove
Red Kite
cattle egret
red−cheeked cordonbleu
Baikal teal
hooded merganser
village weaver

Supp. Fig. 18

F

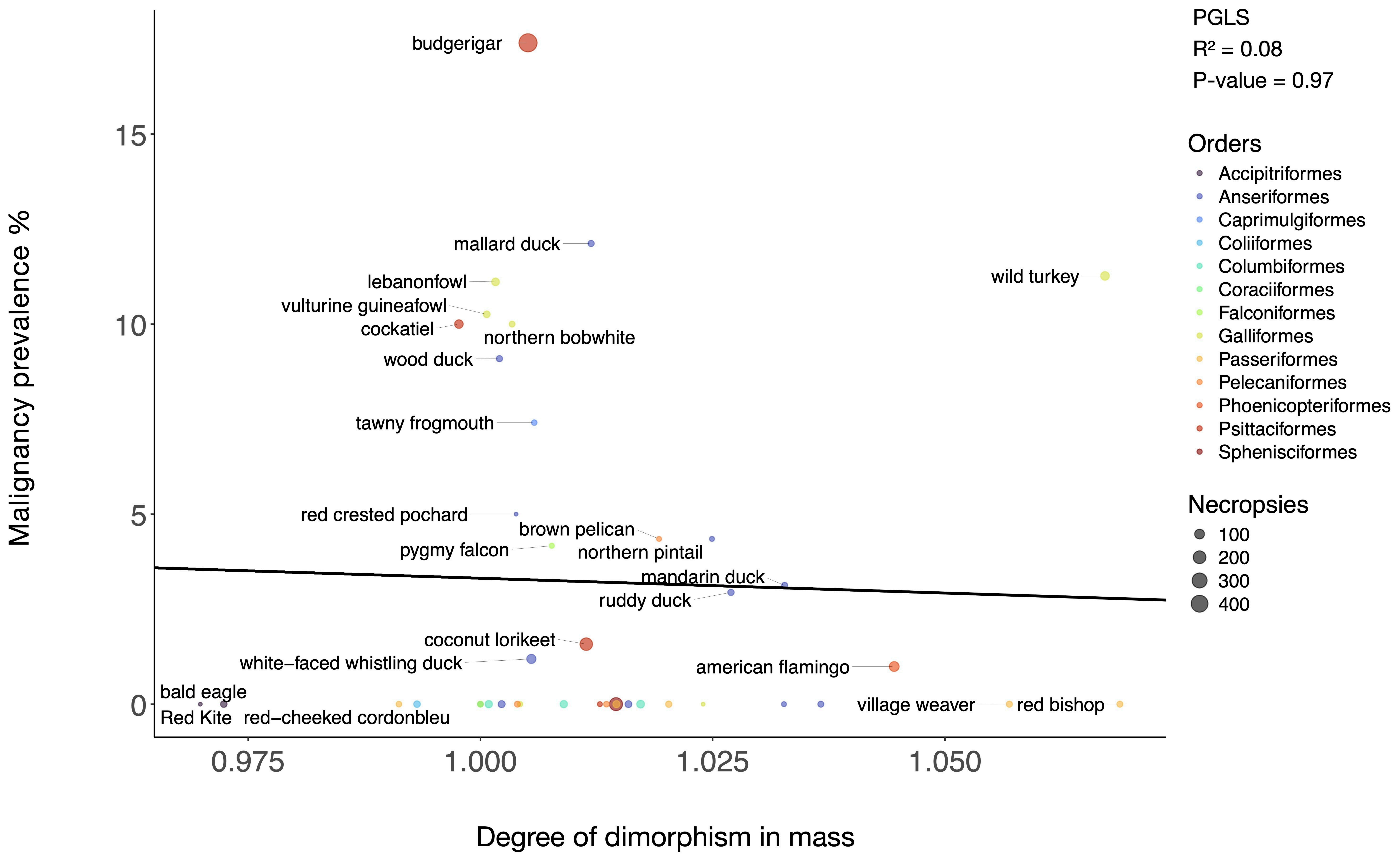
PGLS
R² = 0.08
P-value = 0.97
Orders
Accipitriformes
Anseriformes
Caprimulgiformes
Coliiformes
Columbiformes
Coraciiformes
Falconiformes
Galliformes
Passeriformes
Pelecaniformes
Phoenicopteriformes
Psittaciformes
Sphenisciformes
Necropsies
100
200
300
400
Malignancy prevalence %
Degree of dimorphism in mass
0
5
10
15
0.975
1.000
1.025
1.050
budgerigar
mallard duck
lebanonfowl
wild turkey
vulturine guineafowl
cockatiel
northern bobwhite
wood duck
tawny frogmouth
red crested pochard
brown pelican
pygmy falcon
northern pintail
mandarin duck
ruddy duck
coconut lorikeet
white–faced whistling duck
american flamingo
bald eagle
Red Kite
red–cheeked cordonbleu
village weaver
red bishop

G

Supp. Fig. 18

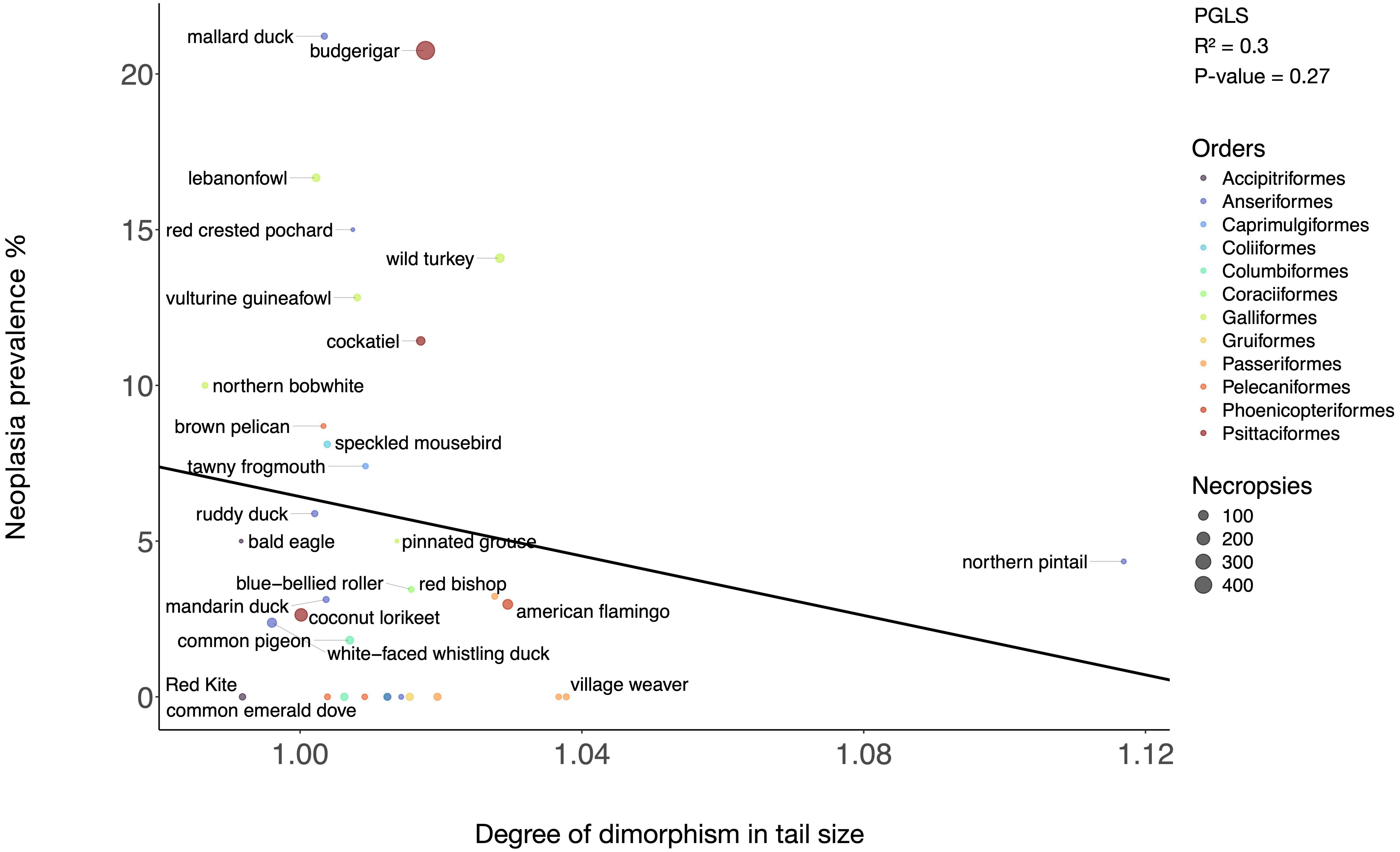
PGLS
R² = 0.3
P-value = 0.27
Orders
Accipitriformes
Anseriformes
Caprimulgiformes
Coliiformes
Columbiformes
Coraciiformes
Galliformes
Gruiformes
Passeriformes
Pelecaniformes
Phoenicopteriformes
Psittaciformes
Necropsies
100
200
300
400
Neoplasia prevalence %
Degree of dimorphism in tail size
0
5
10
15
20
1.00
1.04
1.08
1.12
mallard duck
budgerigar
lebanonfowl
red crested pochard
wild turkey
vulturine guineafowl
cockatiel
northern bobwhite
brown pelican
speckled mousebird
tawny frogmouth
ruddy duck
bald eagle
pinnated grouse
northern pintail
blue−bellied roller
red bishop
mandarin duck
coconut lorikeet
american flamingo
common pigeon
white−faced whistling duck
Red Kite
village weaver
common emerald dove

H

Supp. Fig. 18

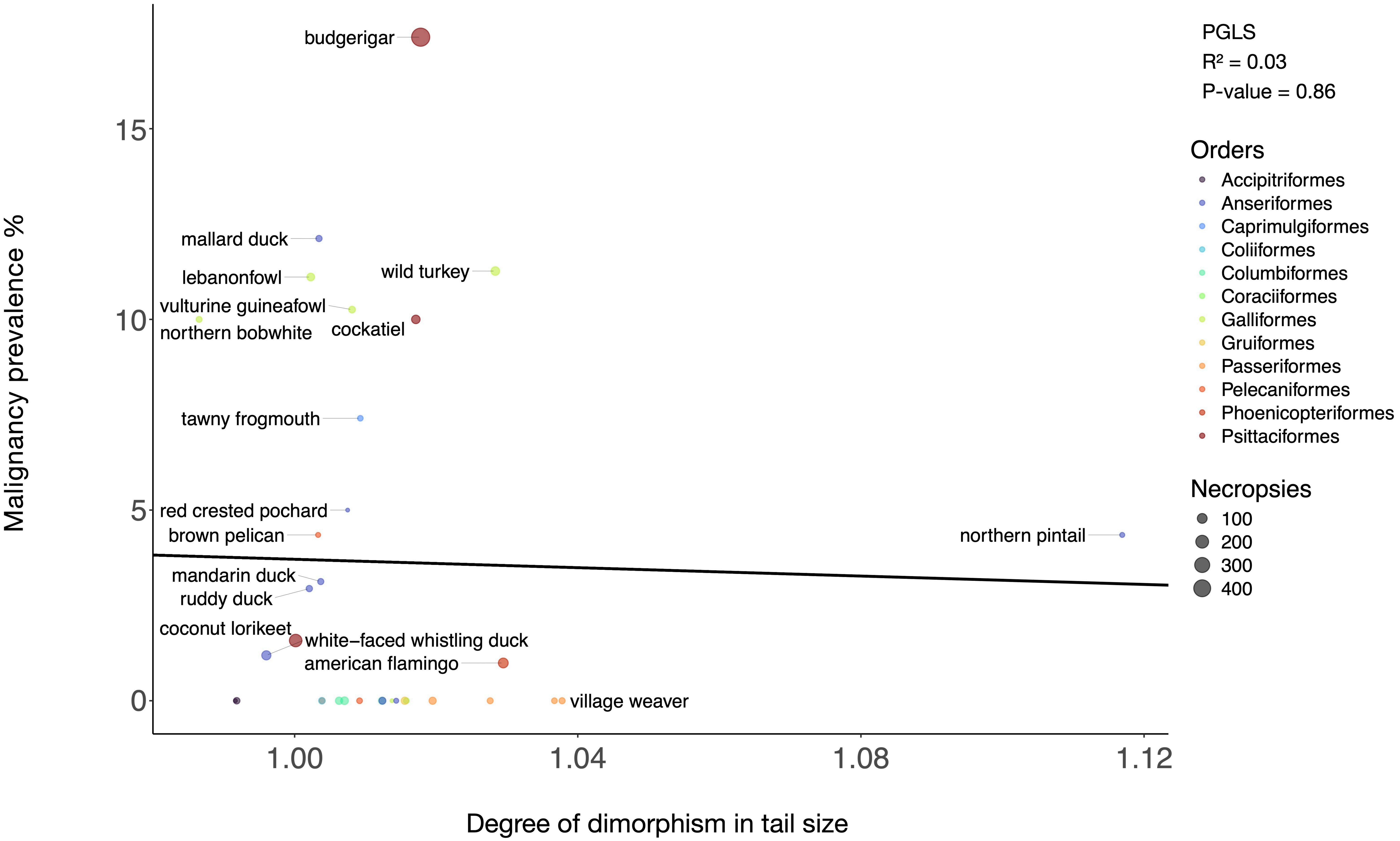
PGLS
R² = 0.03
P-value = 0.86
Orders
Accipitriformes
Anseriformes
Caprimulgiformes
Coliiformes
Columbiformes
Coraciiformes
Galliformes
Gruiformes
Passeriformes
Pelecaniformes
Phoenicopteriformes
Psittaciformes
Necropsies
100
200
300
400
budgerigar
mallard duck
lebanonfowl
wild turkey
vulturine guineafowl
northern bobwhite
cockatiel
tawny frogmouth
red crested pochard
brown pelican
northern pintail
mandarin duck
ruddy duck
coconut lorikeet
white–faced whistling duck
american flamingo
village weaver
Malignancy prevalence %
0
5
10
15
1.00
1.04
1.08
1.12
Degree of dimorphism in tail size
